# Hydrogen Solubility in Metal Membranes: Critical Review and Re-Elaboration of Literature Data

**DOI:** 10.3390/membranes15090273

**Published:** 2025-09-09

**Authors:** Giuseppe Prenesti, Alessia Anoja, Pierfrancesco Perri, Abdulrahman Yaqoub Alraeesi, Shigeki Hara, Alessio Caravella

**Affiliations:** 1 Department of Computer Engineering, Modelling, Electronics and Systems Engineering (DIMES), University of Calabria (UNICAL), Via P. Bucci, Cubo 42C, 87036 Rende, CS, Italy; njalss01e50c352d@studenti.unical.it (A.A.); alessio.caravella@unical.it (A.C.); 2 Institute on Membrane Technology, National Research Council (CNR-ITM), Via P. Bucci, Cubo 17C, 87036 Rende, CS, Italy; 3 Department of Civil, Chemical, Environmental and Materials Engineering (DICAM), Alma Mater Studiorum University of Bologna, Via Zamboni 33, 40126 Bologna, BO, Italy; pierfrancesco.perri@studio.unibo.it; 4 Chemical and Petroleum Engineering Department, College of Engineering, United Arab Emirates University, Al Ain P.O. Box 15551, United Arab Emirates; a.alraeesi@uaeu.ac.ae; 5 Nanomaterials Research Institute, National Institute of Advanced Industrial Science and Technology (AIST), Higashi 1-1-1, Tsukuba 305-8565, Japan; s.hara@aist.go.jp

**Keywords:** metal membranes, hydrogen storage, solubility, alloys embrittlement, sorption

## Abstract

This study undertakes a thorough examination of hydrogen solubility within various metal-alloy membranes, including those based on palladium (Pd), vanadium (V), niobium (Nb), tantalum (Ta), amorphous alloys and liquid gallium (Ga). The analysis aims to outline the strengths and weaknesses of each material in terms of solubility and permeability performance. The investigation began by acknowledging the dual definitions of solubility found in literature: the “*secant method*”, which calculates solubility based on the hydrogen pressure corresponding to a specific sorbed hydrogen loading, and the “*tangent method*”, which evaluates solubility as the derivative (differential solubility) of the sorption isotherm at various square root values of hydrogen partial pressure. These distinct methodologies yield notably different outcomes. Subsequently, a compilation of experimental data for each membrane type is gathered, and these data are re-analysed to assess both solubility definitions. This enabled a clearer comparison and a deeper analysis of membrane behaviour across different conditions of temperature, pressure, and composition in terms of hydrogen solubility in the metal matrix. The re-evaluation presented in this study serves to identify the most suitable membranes for hydrogen separation or storage, as well as to pinpoint the threshold of embrittlement resulting from hydrogen accumulation within the metal lattice. Lastly, recent research has indicated that particularly promising membranes are those fashioned as “sandwich” structures using liquid gallium. These membranes demonstrate resistance to embrittlement while exhibiting superior performance characteristics.

## 1. Introduction

Frequent climate changes and the near petroleum disappearance have pushed the international scientific community to look for a new energy source that can, simultaneously, provide for the future lack of crude oil and not destroy our planet [1,2]. Hydrogen has been identified as a strong candidate because it is highly versatile, as it can be used either as a clean energy carrier or directly as a fuel [3,4].

However, since hydrogen does not naturally exist in its pure form, it has to be produced and eventually separated. One of the main issues of hydrogen production is its separation from other gases, in fact its purity has fulfilled the needs of its application [5,6,7]. Among all the different membranes for hydrogen purification, the dense metal ones are the most interesting due to their hydrogen selectivity and the low operating costs, especially if integrated with other types of devices like adsorption modules [8,9]. Nowadays, novel fabrication techniques has allowed the performance of supported metal membranes to increase considerably with respect to self-supported ones keeping an adequate mechanical resistance [10,11,12,13]. Such performances are usually evaluated in terms of three parameters: solubility, diffusivity and permeability.

In general, these three parameters are not independent of each other, as one can be calculated from the knowledge of the other two. In particular, solubility is related to the ease of a gas to get dissolved into the metal, diffusivity is a measure of how easily gas can move through the metal and permeability is the overall parameter taking into account the ability for a gas to pass through a membrane.

Among these parameters, solubility, which should be evaluated from equilibrium data, is the one that induces more confusion, as it is found to be defined in different ways in the literature. Specifically, in some contexts of science and technology, solubility is referred to as the amount of gas absorbed in a dense/microporous lattice per amount of gas in the fluid phase, measured in several possible combinations of convenient units.

Differently, according to the solution-diffusion model (see Equation (18) reported in the next section), given an equilibrium curve reporting the absorbed amount of gas—expressed, for example, in terms of concentration in the dissolved phase—versus the corresponding gas-phase amount—expressed, for example, in terms of partial pressure –, solubility is defined as the tangent of such a curve in each point, for whose reason it changes, in general, with the gas content in the lattice [14].

It is our opinion that this confusion could be overcome if the former solubility were referred to as simply “*solubility*”, whereas the latter were referred to as “*differential solubility*”, in analogy with the field of electrical engineering, where it is distinguished between “*resistance*”, which is the simple ratio of voltage *V* over current *I* (*V/I*), and “*differential resistance*”, which is the local derivative *dV/dI* [15].

In the particular case of hydrogen—and, in general, of all the gases behaving in the same way when interacting with a certain surface –, the content in the gas phase is quantified using the square root of its partial pressure, which arises from the dissociative adsorption onto the metal surface [16].

In the current study, hydrogen solubility in different solid matrices is calculated using both the tangent and secant methods to identify any potential differences. Considering different metal matrices is important because palladium—one of the most commonly used metals for membranes—is very expensive and can present issues under certain operating conditions. Therefore, other metals or alloys should be used as substitutes [17].

## 2. Permeation Mechanism

The hydrogen purification membrane systems have almost the same structure. We can recognize three different parts:

- the feed side
- the membrane
- the permeate side

**Figure 1:**
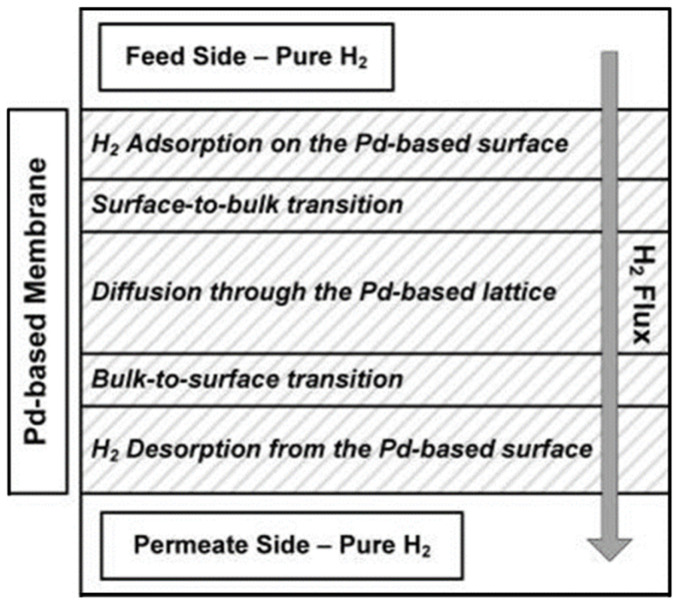
Step-by-step breakdown of hydrogen permeation across a self-supported Pd membrane, reproduced from [16] with permission of Elsevier.

In the feed side there is a mixture of different gases from which hydrogen has to be separated, while in the permeate side there is pure hydrogen. Instead, some hydrogen profiles are established through the membrane because of the resistances to the flux due to the different steps composing the overall permeation process [18] (Figure 1).

**Figure 2:**
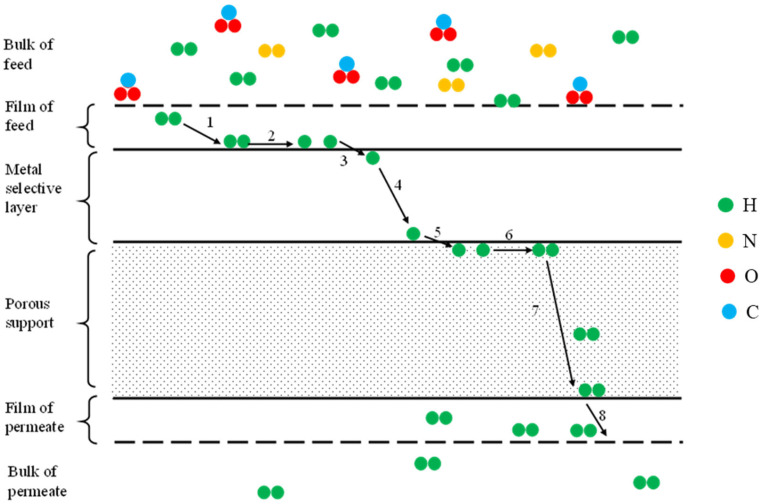
Mechanism of hydrogen permeation through a supported metal membrane (solution-diffusion mechanism).

The mechanism for hydrogen purification consists of seven steps (Figure 2):

1. mass transfer in the fluid phase of the film just near the interface in the feed side
2. dissociative adsorption
3. absorption
4. diffusion
5. re-associative desorption
6. mass transfer in the fluid phase of the multilayered porous support
7. mass transfer in the fluid phase of the film just near the interface in the permeate side.

Molecular hydrogen and nitrogen (or other gases) are put above the metal surface (1), hydrogen dissociates and get adsorbed (2) and, under the driving force of a pressure, concentration, or electrical potential gradients, pass into the metal bulk and diffusion starts (3–4), after the membrane the reverse process starts (5) [18].

We can have unsupported and supported membranes. The first type can be prepared, for example, from thick metal foils, which are cold-rolled and welded, in order to obtain a certain thickness, usually between 50–150 μm [19].

In supported membranes, the very thin metal layer is placed on a porous material, such as alumina, stainless steel and glasses, to ensure it handles or resists the high mechanical stress during the process, and without it the thin layer loses its mechanical stability.

It is essential that ultrathin membranes are free of defects; otherwise, substances other than hydrogen could also pass through, and hence it affects the good functioning of the module resulting in impure hydrogen at the permeate side [20].

## 3. Hydrogen Sorption Mechanism in Metal Membranes

Hydrogen sorption in metal membrane can be divided in two steps, the first of which involves a dissociative chemical adsorption:

- adsorption on to the metal surface

(1) $$r_{1}:H_{2}+2\bar{X}↔2H\bar{X}\;\to \text{(adsorption)}$$

- absorption into the metal bulk

(2) $$r_{2}:H\bar{X}+\overset{̿}{X}↔H\overset{̿}{X}+\bar{X}\to \text{(absorption)}$$

The overall *r*_1_–*r*_2_ transition represents the transition from gas to dissolved phase and takes the name of *sorption* (Equation (3)):

(3) $$r_{ov}:H_{2}+\overset{̿}{X}↔2H\overset{̿}{X}\to \text{(sorption)}$$

where *H*_2_ is hydrogen in gas phase, *H* is the dissociated hydrogen, $\bar{X}$ is the adsorption site, $H\bar{X}$ is the adsorbed hydrogen, $\overset{̿}{X}$ is the absorption site and $H\overset{̿}{X}$ is the absorbed hydrogen.

The equilibrium constant of Equation (1) expressed in terms of activity of species is:

(4) $$K_{1}\;=\;\frac{{\bar{a}}_{H}^{2}}{a_{H_{2}}{\bar{a}}_{v}^{2}}$$

where *a_i_* are the activity of the species in the gas phase and ${\bar{\alpha }}_{i}$ are the activity of the species in the adsorbed phase.

The hydrogen activity in each phase is related to the respective composition through the activity coefficients, which are complex functions of composition based on the particular thermodynamic model describing the system. As for the gas phase, it is convenient to work directly with fugacity and related fugacity coefficient. Therefore, the complete expression for *K*_1_ is:

(5) $$K_{1}\;=\;\frac{{\left({\bar{\gamma }}_{H}{\bar{\theta }}_{H}\right)}^{2}}{\frac{f_{H_{2}}}{P^{0}}{\left({\bar{\gamma }}_{v}{\bar{\theta }}_{v}\right)}^{2}}\;=\;\frac{{\left({\bar{\gamma }}_{H}{\bar{\theta }}_{H}\right)}^{2}}{\frac{\phi_{H_{2}}P_{H_{2}}}{P^{0}}{\left({\bar{\gamma }}_{v}{\bar{\theta }}_{v}\right)}^{2}}\;=\;\frac{1}{\phi_{H_{2}}}{\left(\frac{{\bar{\gamma }}_{H}}{{\bar{\gamma }}_{v}}\right)}^{2}\frac{{\left({\bar{\theta }}_{H}\right)}^{2}}{\frac{P_{H_{2}}}{P^{0}}{\left({\bar{\theta }}_{v}\right)}^{2}}$$

where *θ_H_* is the surface coverage, which is a measure of the surface concentration, and *ϕ_H_*_2_ is the fugacity coefficient in the gas phase, *γ_i_* are the activity coefficient of species *i*, *P_H_*_2_ is the hydrogen partial pressure and *P*_0_ is the reference pressure. Under Langmuir’s hypotheses, we have:

(6) $$\frac{1}{\phi_{H_{2}}}{\left(\frac{{\bar{\gamma }}_{H}}{{\bar{\gamma }}_{v}}\right)}^{2}\approx 1$$

${\bar{\theta }}_{H}\;$ can be expressed from Equation (4):

(7) $${\bar{\theta }}_{H}\;=\;{\bar{\theta }}_{v}\sqrt{K_{1}\frac{P_{H_{2}}}{P^{0}}}$$

The population balance on the surface will be:

(8) $$1\;=\;{\bar{\theta }}_{H}\;+\;{\bar{\theta }}_{v}\;=\;{\bar{\theta }}_{v}\sqrt{K_{1}\frac{P_{H_{2}}}{P^{0}}}\;+{\;\bar{\theta }}_{v}$$

From Equation (8), ${\bar{\theta }}_{v}$ is obtained as:

(9) $${\bar{\theta }}_{v}\;=\;\frac{1}{1\;+\;\sqrt{K_{1}\frac{P_{H_{2}}}{P^{0}}}}$$

Combining Equations (4) and (9) and rearranging the result is:

(10) $${\bar{\theta }}_{H}\;=\;\frac{{\bar{C}}_{H}}{{\bar{C}}_{m}}\;=\;\frac{\sqrt{K_{1}\frac{P_{H_{2}}}{P^{0}}}}{1\;+\;\sqrt{K_{1}\frac{P_{H_{2}}}{P^{0}}}}\;=\;\frac{\sqrt{K_{1}^{*}P_{H_{2}}}}{1\;+\;\sqrt{K_{1}^{*}P_{H_{2}}}}$$

(11) $$K_{1}^{*}\;=\;\frac{K_{1}}{P^{0}}$$

Above around 100 °C, the reaction in Equation (2) can be considered at equilibrium [21]:

(12) $$r_{2}\;=\;k_{f_{2}}{\bar{\theta }}_{H}\left(1\;-\;{\overset{̿}{\xi }}_{H}\right)\;-{\;k}_{r_{2}}\left(1\;-\;{\bar{\theta }}_{H}\right){\overset{̿}{\xi }}_{H}\;=\;0$$

where *k_f_*_2_ and *k_r_*_2_ are respectively the forward and reverse kinetic constant.

From Equation (12), ${\overset{̿}{\xi }}_{H}$, that is the amount of hydrogen is the absorbed phase, can be written as:

(13) $${\overset{̿}{\xi }}_{H}\;=\;\frac{{\overset{̿}{C}}_{H}}{{\overset{̿}{C}}_{m}}\;=\;\frac{K_{2}{\bar{\theta }}_{H}}{K_{2}{\bar{\theta }}_{H\;}+\;\left(1\;-\;{\bar{\theta }}_{H}\right)}$$

Combining Equations (10) and (13), the following expression is obtained:

(14) $${\overset{̿}{\xi }}_{H}\;=\;\frac{K_{2}\frac{\sqrt{K_{1}^{*}P_{H_{2}}}}{1\;+\;\sqrt{K_{1}^{*}P_{H_{2}}}}}{K_{2}\frac{\sqrt{K_{1}^{*}P_{H_{2}}}}{1\;+\;\sqrt{K_{1}^{*}P_{H_{2}}}}\;+\;\left(1\;-\;\frac{\sqrt{K_{1}^{*}P_{H_{2}}}}{1\;+\;\sqrt{K_{1}^{*}P_{H_{2}}}}\right)}$$

Under Henry’s hypotheses (infinite dilution), Equation (14) becomes Sieverts’ law (Equation (15)):

(15) $${\overset{̿}{\xi }}_{H}\;=\;{\overset{̿}{H}}_{H}\sqrt{P_{H_{2}}}$$

where ${\overset{̿}{H}}_{H}$ is Henry’s constant for hydrogen.

## 4. Solubility Definition Based on the Solution-Diffusion Model

The expressions reported in the previous section represent the kinetic and thermodynamic relationships describing the system. On the other hand, once hydrogen is dissolved in the metal bulk, a mass flux is generated under a certain difference of chemical potential, promoted by the difference of partial pressure in case of membrane. These steps—sorption and diffusion—occur in sequence, and this is the reason why the mathematical description of permeation in these conditions is called “*solution-diffusion*” model, based on which the flux through membrane can be expressed in terms of solubility and diffusivity [22]. For this purpose, the starting point is Fick’s law (Equation (16)):

(16) $$J_{H}\;=\;-D_{H}\frac{\partial {\bar{\bar{C}}}_{H}}{\partial x}\;=\;-D_{H}^{T}\frac{\partial \ln{\bar{\bar{a}}}_{H}}{\partial \ln{\bar{\bar{C}}}_{H}}\frac{\partial {\bar{\bar{C}}}_{H}}{\partial x}\;=\;-D_{H}^{T}\Gamma_{H}\frac{\partial {\bar{\bar{C}}}_{H}}{\partial x}$$

where *D_H_* is the hydrogen diffusion coefficient in the metal matrix, ${\overset{̿}{C}}_{H}\;$ is the hydrogen concentration in the metal bulk, ${\overset{̿}{a}}_{H}\;$ is the hydrogen activity in the metal bulk and *Γ_H_* is a thermodynamics factor.

At a certain temperature, the diffusion coefficient (diffusivity) in non-ideal conditions (i.e., far from the infinite dilution) is a function of concentration, whose correction with respect to the ideal conditions is given by the thermodynamic factor *Γ_H_*.

The concentration gradient can be expressed as a function of the gas-phase partial pressure according to Equation (17), where the square root arises from Equation (15):

(17) $$\frac{\partial {\bar{\bar{C}}}_{H}}{\partial x}\;=\;\frac{\partial {\bar{\bar{C}}}_{H}}{\partial \sqrt{P_{H_{2}}}}\frac{\partial \sqrt{P_{H_{2}}}}{\partial x}$$

By doing that, diffusivity and solubility terms are made explicit (Equation 18):

(18) $$J_{H}\;=\;-\underset{\mathrm{Diffusivity}}{\underset{︸}{D_{H}^{T}\frac{\partial \ln{\bar{\bar{a}}}_{H}}{\partial \ln{\bar{\bar{C}}}_{H}}}}\cdot \underset{\mathrm{Solubility}}{\underset{︸}{\frac{\partial {\bar{\bar{C}}}_{H}}{\partial \sqrt{P_{H_{2}}}}}}\cdot \frac{\partial \sqrt{P_{H_{2}}}}{\partial x}$$

where the meaning of the parameters is reported in Equations (19) and (20):

(19) $$\begin{array}{l} D_{H}^{T}\equiv \mathrm{Intrinsic}\;(\mathrm{zero-loading})\;\mathrm{Diffusivity}\\ \frac{\partial \ln{\bar{\bar{a}}}_{H}}{\partial \ln{\bar{\bar{C}}}_{H}}\equiv \mathrm{Thermodynamic}\;\mathrm{Factor}\;\Gamma_{H}\\ D_{H}^{T}\frac{\partial \ln{\bar{\bar{a}}}_{H}}{\partial \ln{\bar{\bar{C}}}_{H}}\;=\;\mathrm{Diffusivity}\;D_{H} \end{array}$$

(20) $$\frac{\partial {\bar{\bar{C}}}_{H}}{\partial \sqrt{P_{H_{2}}}}\equiv \mathrm{Solubility}\;S_{H}$$

Combining the previous expressions, we obtain the final form for permeating flux in terms of permeability (Equation (21)):

(21) $$\begin{array}{l} J_{H}\;=\;-D_{H}^{T}\Gamma_{H}S_{H}\cdot \frac{\partial \sqrt{P_{H_{2}}}}{\partial x}\;==\;-D_{H}S_{H}\cdot \frac{\partial \sqrt{P_{H_{2}}}}{\partial x}\;=\;-\phi_{H}\frac{\partial \sqrt{P_{H_{2}}}}{\partial x}\\ \phi_{H}\;=\;\mathrm{Permeability} \end{array}$$

## 5. Re-Elaboration and Analysis of Solubility Data

According to the expressions reported in the previous section, solubility of hydrogen in metals is defined as the derivative of the concentration in the metal bulk with respect to the square root of the hydrogen partial pressure in the gas phase. More generally, given an isotherm in the form {*adsorbate concentration* vs. *fugacity*}, the solubility plot can be derived by taking the derivative of the variable in the ordinate with respect to that in the abscissa.

However, many studies of the literature (inappropriately) denote solubility as the hydrogen content in solution (the metal bulk, in the present work), which creates certain confusion even in the specialised scholars. On the other hand, such an inappropriate definition of solubility is so common that nowadays has become a sort of state of the art.

**Figure 3:**
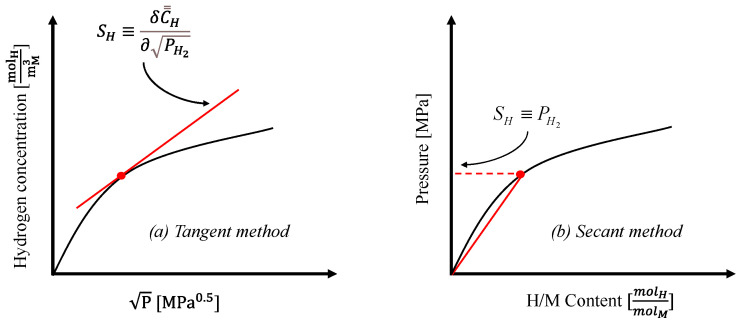
Solubility defined and calculated by: (**a**) *Tangent method*, and (**b**) *Secant method*.

Therefore, in the data analysis reported in the present work, we use two ways of representation of solubility: one based on the definition reported in Equation (20), and the other one based on the content of the species in solution. These two approaches are denoted here according to what shown in Figure 3, where the former is referred to as “*tangent method*” (derivative), whereas the latter is referred to as “*secant method*” (hydrogen content).

The two methods can be resumed as follows:

- Tangent Method: solubility is calculated as the pointwise derivative of the hydrogen concentration in the metal matrix with respect to the square root of the applied pressure. This method provides a local measure of the tendency of the material to be able to absorb further hydrogen at each pressure value.
- Secant Method: solubility is calculated as the slope of the secant line drawn from the origin (zero pressure, zero hydrogen content) to each data point, considering hydrogen concentration in the metal matrix versus pressure. This method provides the hydrogen specific amount accepted in the material at each pressure.

The meaning of the former is clear (Figure 3a), whilst that of the latter is related to the fact that the concentration in the metal bulk is calculated by the intersection of a straight line starting from zero (secant). It should be specified that in the literature there are even different ways to represent a sorption isotherm using different quantities and/or switching abscissa and ordinate.

### Calculation Methodology

In this work, for uniformity of representation, all data are represented in a {*concentration* vs. *square root of hydrogen partial pressure*} for the solubility calculated by the tangent method, whereas the data are represented in a {*partial pressure* vs. *composition in metal*} for the other method. Such a difference implies that the two types of solubility have different units: the former in mol m^−3^ Pa^−0.5^, whereas the latter in MPa.

As for the physical meaning of the two different definitions of solubility, whilst the one calculated by the secant method is obvious, that calculated by the tangent one represents the tendency of the material to accept more hydrogen. Based on this concept, in the proximity of a plateau of a sorption isotherm, such a solubility tends to zero, as all the sorption sites are almost completely occupied.

It is remarked that the solubility data calculated by the tangent method are necessary to evaluate the diffusion coefficient from the knowledge of the corresponding permeability, as clearly shown in Hara et al. [23]. In fact, the direct calculation of the diffusion coefficient in non-ideal conditions (i.e., far from infinite-dilution conditions) from experimental data is not straightforward.

Therefore, two types of independent experimental campaigns have to be conducted to determine solubility and permeability, for the former of which there is a lack of literature data owing to an insufficient elaboration of the experimental isotherms. The data shown in the following sections are also reported in some of the tables in the Supplementary Materials along with other additional data.

## 6. Results and Discussion

### 6.1. Pd-Based Membranes

In this section, data on hydrogen solubility in Pd alloys are re-elaborated to show its behaviour as a function of the square root of the hydrogen pressure for different values of temperature. It is specified that the reason for using the hydrogen partial pressure square root lies in the dissociative adsorption of molecular hydrogen on the metal surface (Equation (1)).

**Figure 4:**
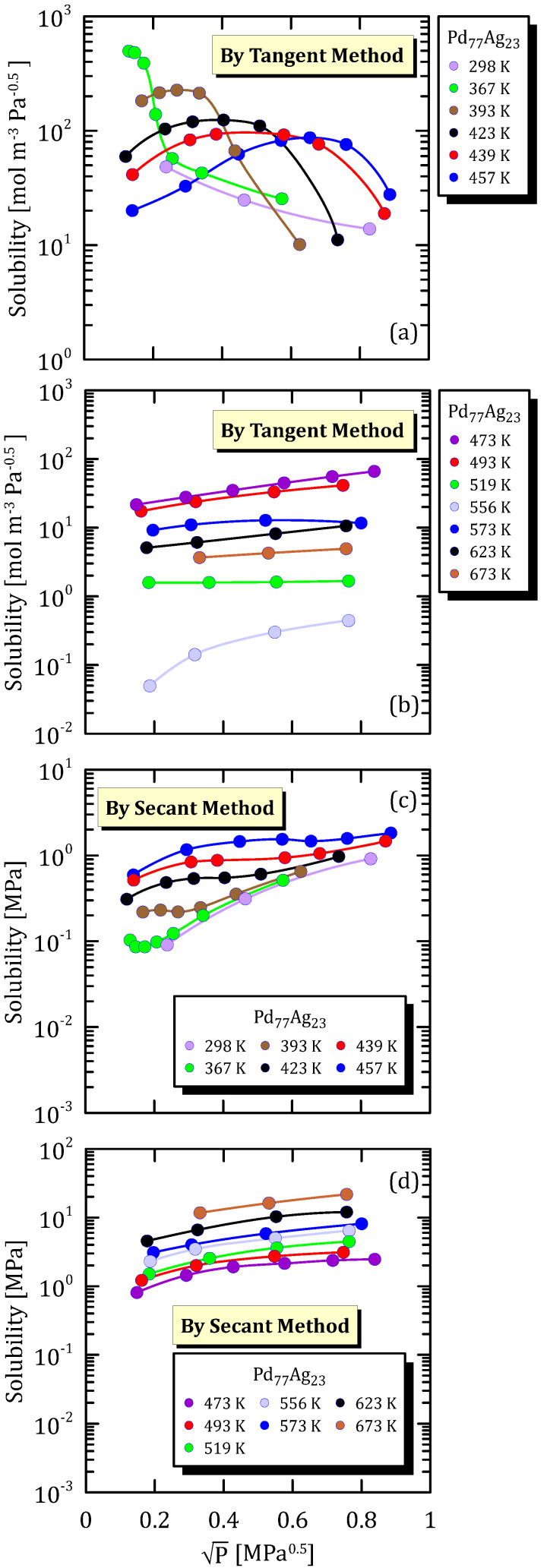
Solubility vs P^0.5^ of Pd_77_Ag_23_ at different temperatures [24] (see Table S1). The various plots—(**a**,**c**): low temperature, and (**b**,**d**) high temperature—are split just for a higher clarity.

As mentioned above, the solubility values calculated by the tangent method is obtained by evaluating the derivative at each point of the corresponding sorption isotherms. Figure 4a,b show the same alloy at different values of temperature, which are shown in two different plots just for a matter of clarity. In particular, it can be observed that, at sufficiently low temperature and pressure, the hydrogen solubility calculated by the tangent method shows a non-monotonic behaviour, with the presence of a maximum with increasing hydrogen pressure.

This behaviour can be explained by considering that, as pressure increases in a sufficiently low pressure range, the tendency for hydrogen to be sorbed in the metal lattice is strong, as the majority of sorption sites are available, this corresponding to an isotherm that is more than linear at low pressure.

Differently, as pressure becomes progressively higher, the sorption tendency becomes weaker and, thus, the sorption isotherm becomes convex and starts tending to a plateau, this implying a negative second derivative.

However, at sufficiently low temperature, the solubilisation tendency decreases with increasing pressure as the dissociative adsorption is not fast enough to boost hydrogen atoms in the metal lattice. In other words, the adsorption of the molecular hydrogen is more physical than chemical.

An interesting aspect to notice is that, as the temperature increases, the position of the maximum shift to the right (higher pressure values). This is again explaining by considering that temperature boosts the dissociative adsorption, whose kinetic constant follows an Arrhenius-type functionality.

Figure 4c,d shows that hydrogen solubility into the metal lattice calculated by the secant—i.e., the hydrogen content in it—method increases with increasing pressure and temperature, at least in the conditions ranges considered in the present study. The effect of pressure is quite obvious, as the higher the hydrogen pressure, the higher the content in the metal lattice. As for the temperature effect, since the dissociative adsorption is favoured by increasing temperature, more hydrogen can pass from the adsorbed state to the dissolved one in the metal lattice.

**Figure 5:**
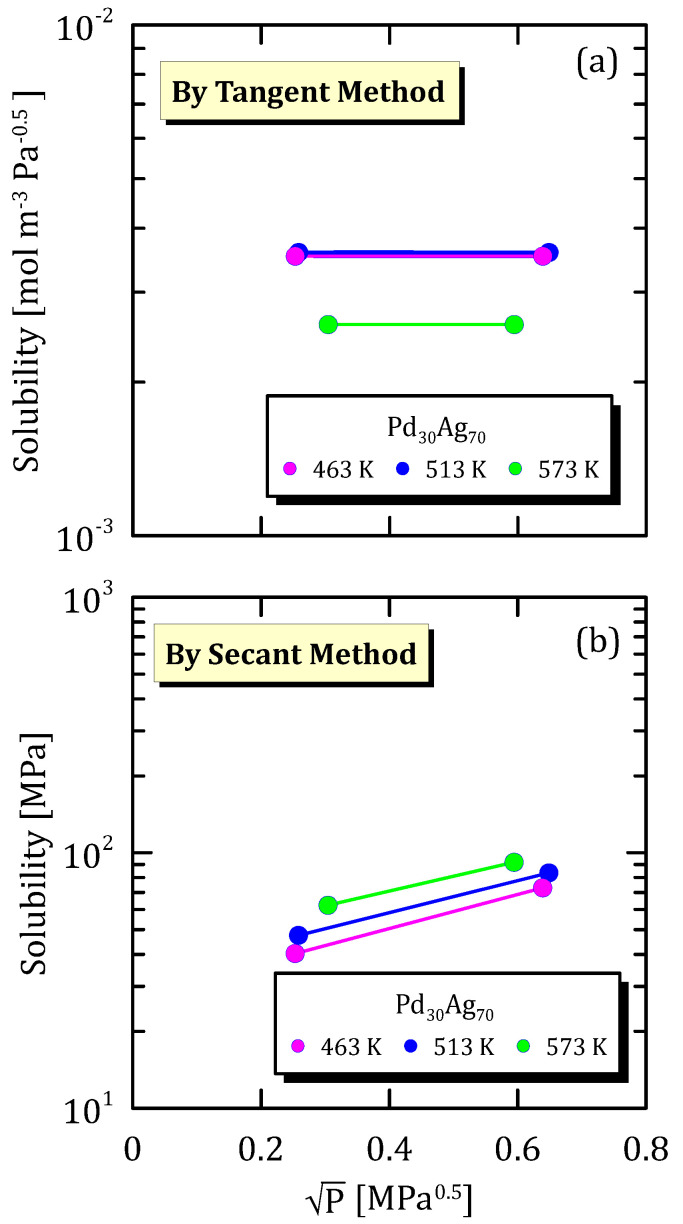
Solubility vs P^0.5^ of Pd_30_Ag_70_ at different temperatures [24] (see Table S2).

Figure 5a shows the hydrogen solubility in Pd_30_Ag_70_ alloy calculated by tangent method, which looks constant with pressure in the considered pressure range. This behaviour corresponds to a linear part of the sorption isotherm, which is depicted in Figure 5b.

### 6.2. V-Based Membranes

The following figures show the solubility behaviour for vanadium alloys with the square root pressure at different temperatures. Some data of deuterium solubility in V alloys are reported as well [25].

**Figure 6:**
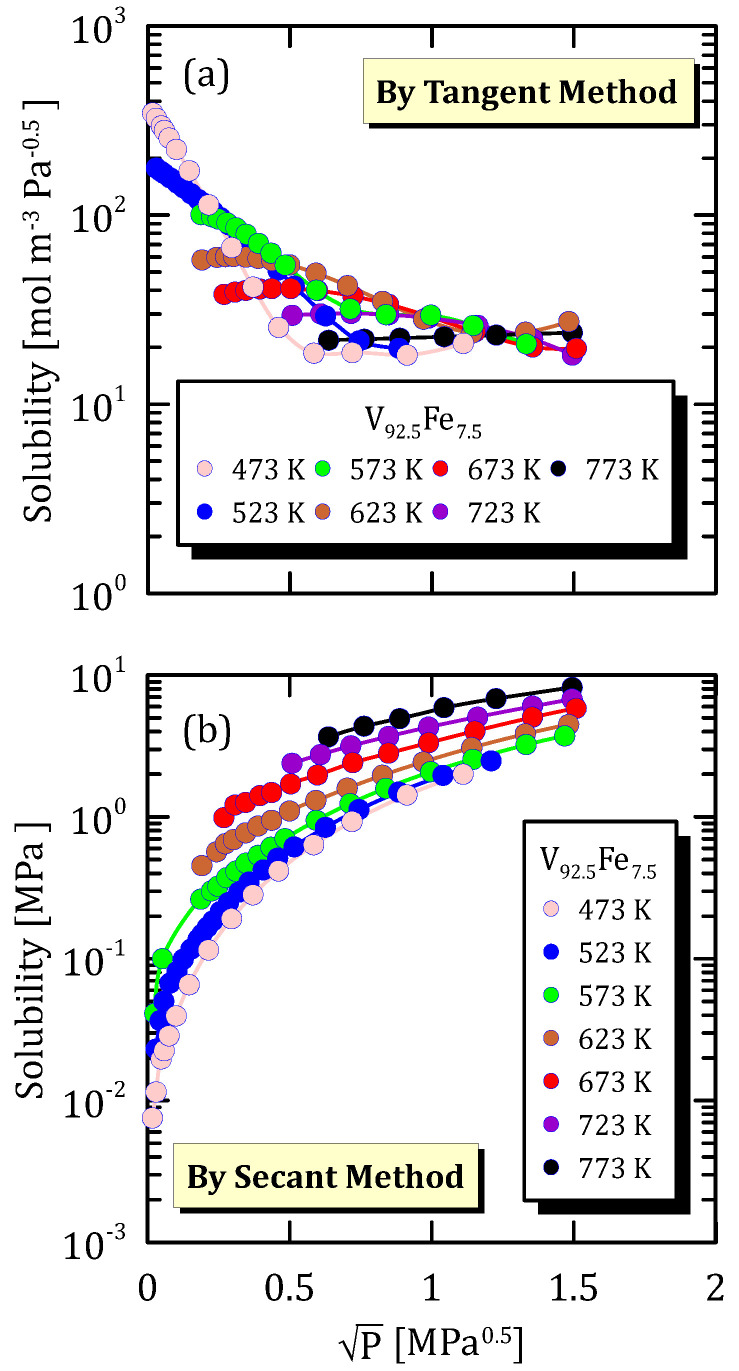
Solubility vs P^0.5^ of V_92.5_Fe_7.5_ at different temperatures [25] (see Table S4).

Figure 6b shows that, if solubility is calculated by secant method, it increases as the temperature gets higher, whereas the behaviour is opposite if solubility is calculated through the tangent method (Figure 6a). This is in line with the fact that the solubility calculated by the tangent method is proportional to the derivative of the solubility evaluated by the secant method.

**Figure 7:**
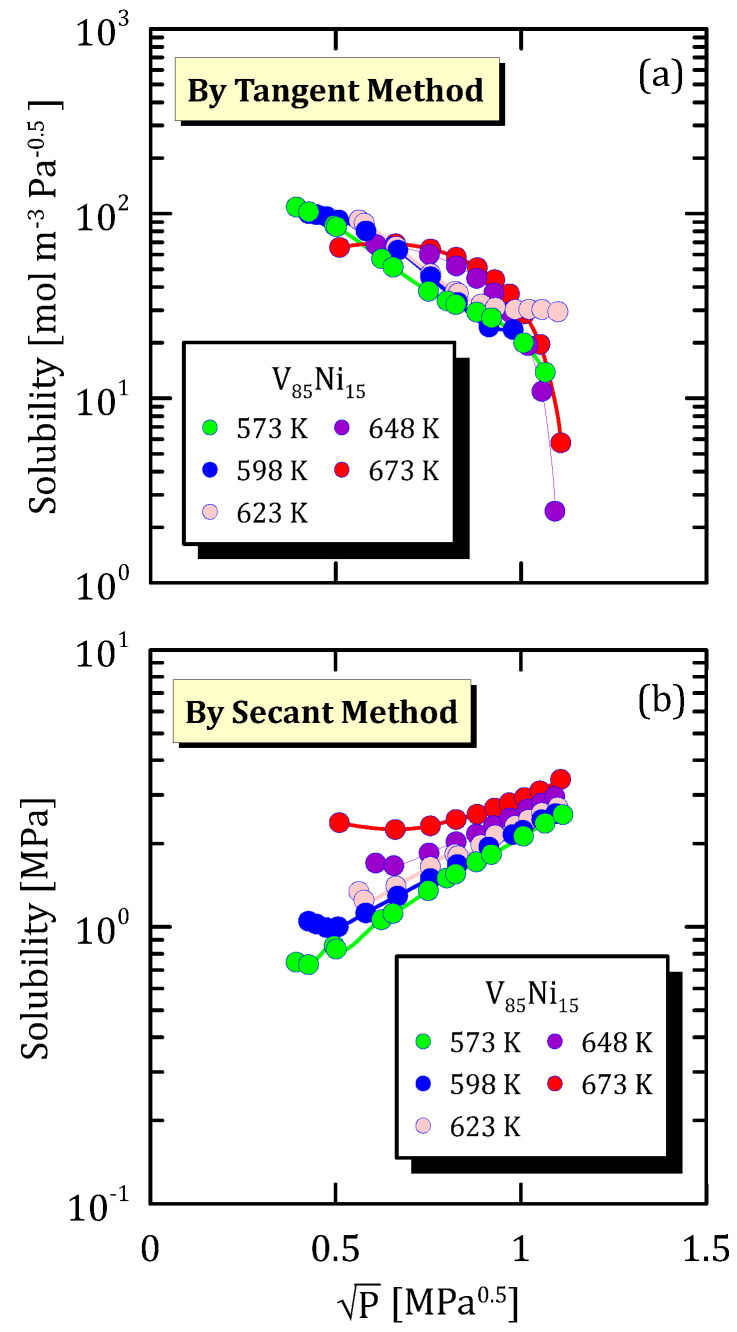
Solubility vs P^0.5^ of V_85_Ni_15_ at different temperatures [26] (see Table S6).

Figure 7b highlights that, also for this V_85_Ni_15_ alloys, if solubility is calculated by secant method, it increases as the temperature gets higher. Moreover, a high pressure discourages solubility. At low pressure, solubility calculated by secant the method shows a sort of plateau, this indicating a possible structural change of hydrogen sorption sites. The behaviour is different if solubility is calculated through the tangent method (Figure 7a), in fact, solubility decreases with pressure.

**Figure 8:**
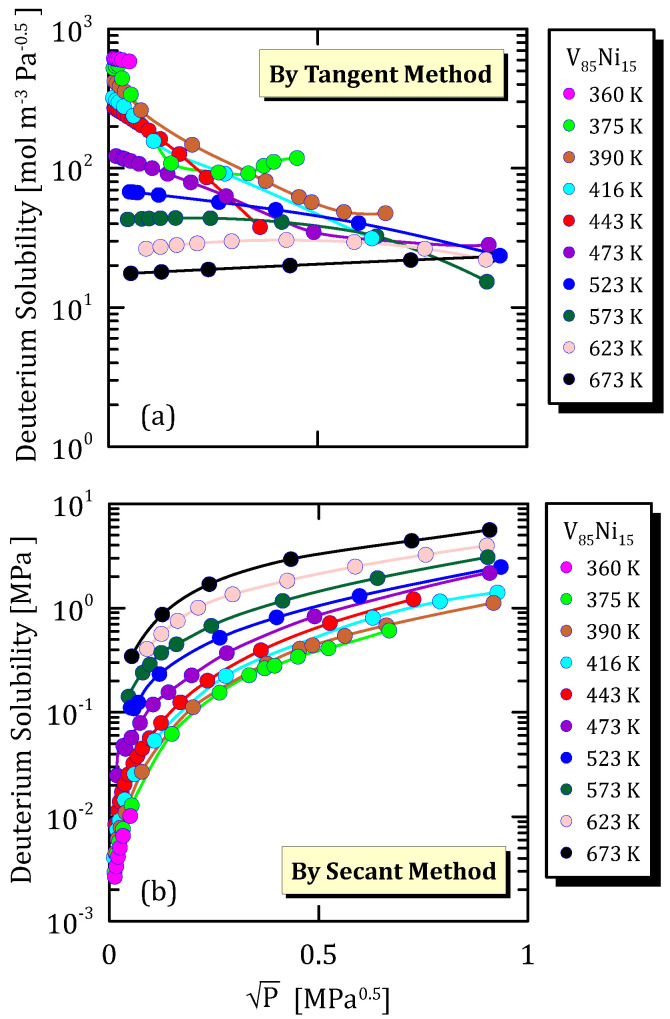
Solubility vs P^0.5^ of V_85_Ni_15_ at different temperatures (deuterium) [27] (see Table S9).

Figure 8a,b points out how the behaviour of deuterium solubility in V_85_Ni_15_ alloys varies with temperature and, once again, if deuterium solubility is calculated by secant method, it increases with temperature. Moreover, deuterium solubility is observed to be slightly higher than the protium one in the same alloy at the same temperature.

**Figure 9:**
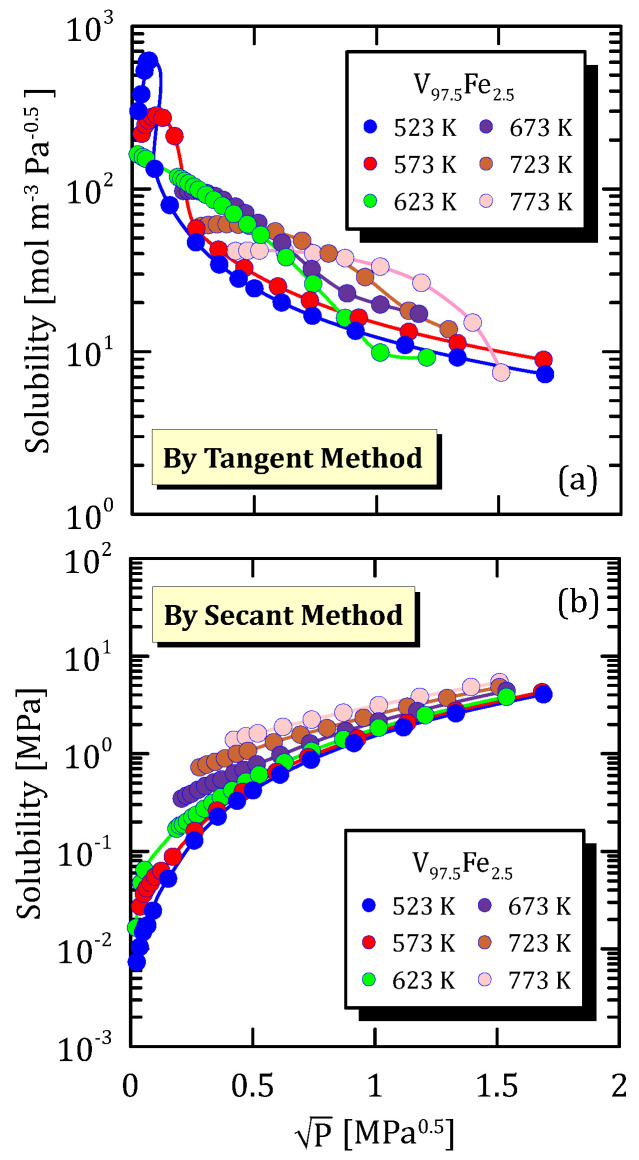
Solubility vs P^0.5^ of V_97.5_Fe_2.5_ at different temperatures [25] (see Table S3).

**Figure 10:**
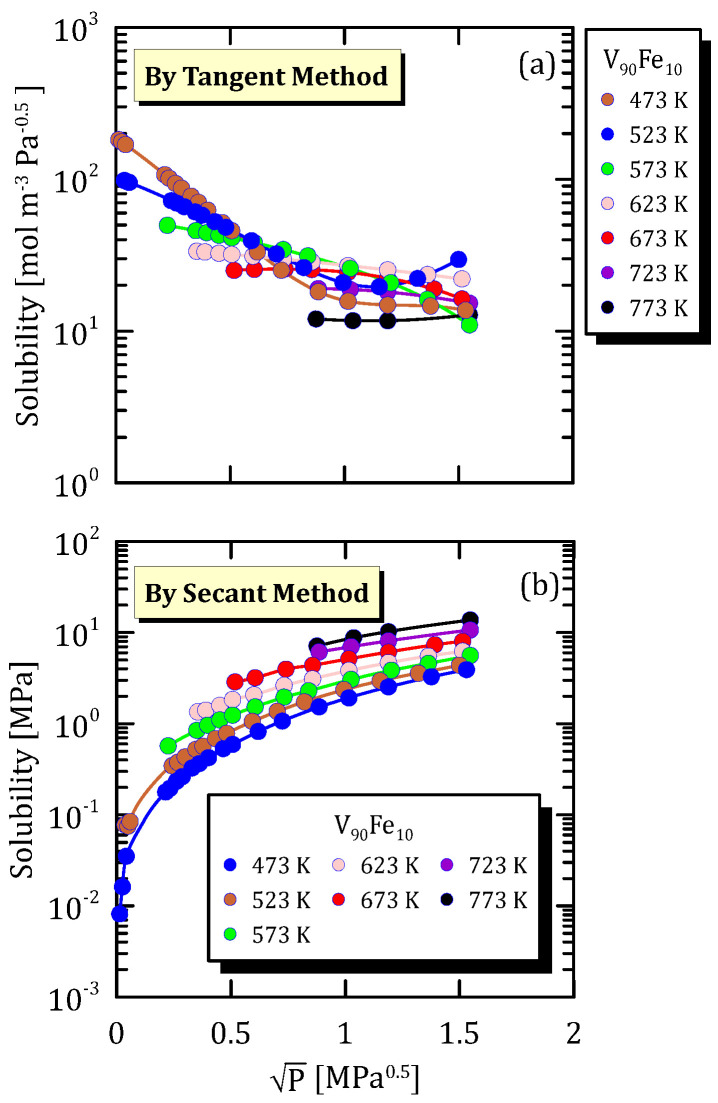
Solubility vs P^0.5^ of V_90_Fe_10_ at different temperatures [25] (see Table S5).

Comparing Figure 9a and Figure 10a, it is possible to notice that the hydrogen solubility maximum, that was evident at low temperature for V_97.5_Fe_2.5_ alloy, completely disappears when the iron content increases from 2.5 to 10. This occurs for the same reasons as those stated analysing the Pd-based alloys in the previous sections. Hydrogen solubility calculated though the secant method (Figure 9b and Figure 10b) increases with pressure.

**Figure 11:**
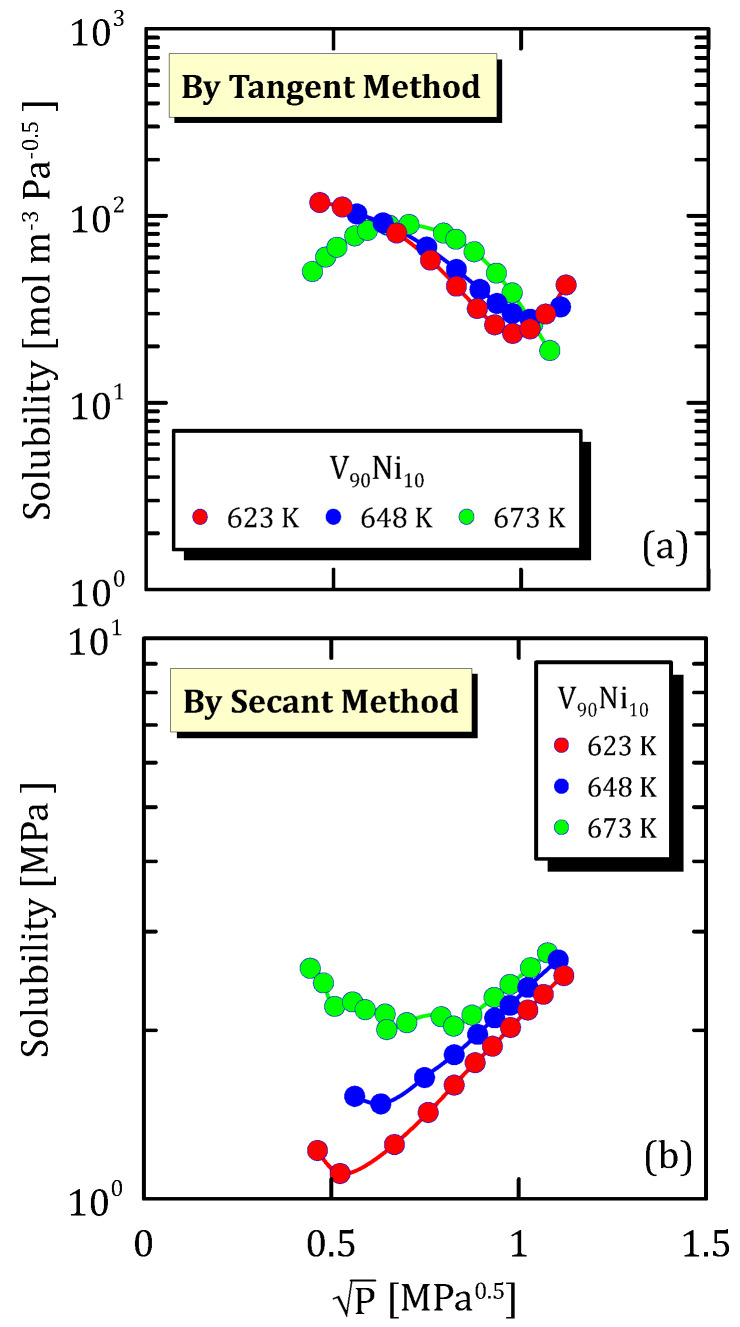
Solubility vs P^0.5^ of V_90_Ni_10_ at different temperatures [26] (see Table S7).

As for the behaviour depicted in Figure 11, also in this case a high temperature together with a high partial pressure hinders the dissociative adsorption of hydrogen onto the metal surface and, thus, hydrogen solubility, calculated by tangent method (Figure 11a), reaches a maximum and then decreases rapidly. For lower temperature, on the contrary, hydrogen solubility shows a minimum, meaning that a high value of the square root of the pressure alone favours the hydrogen sorption in the metal. 

**Figure 12:**
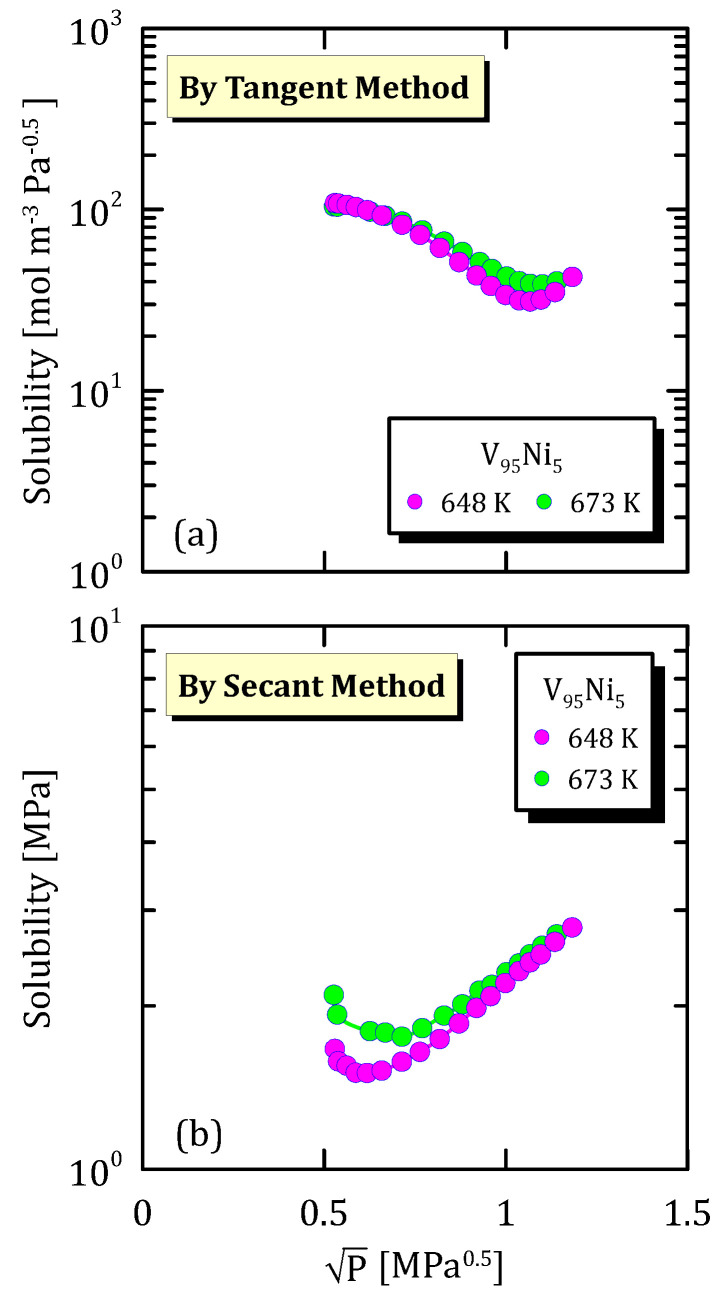
Solubility vs P^0.5^ of V_95_Ni_5_ at different temperatures [26] (see Table S8).

Figure 11a confirms the behaviour reported in Figure 12a, but, at the same time, states that a lower content of nickel allows preventing that the hydrogen dissociative desorption becomes the limiting step in the permeation mechanism. The solubility calculated with the secant method (Figure 11b and Figure 12b) shows a minimum at low pressures.

**Figure 13:**
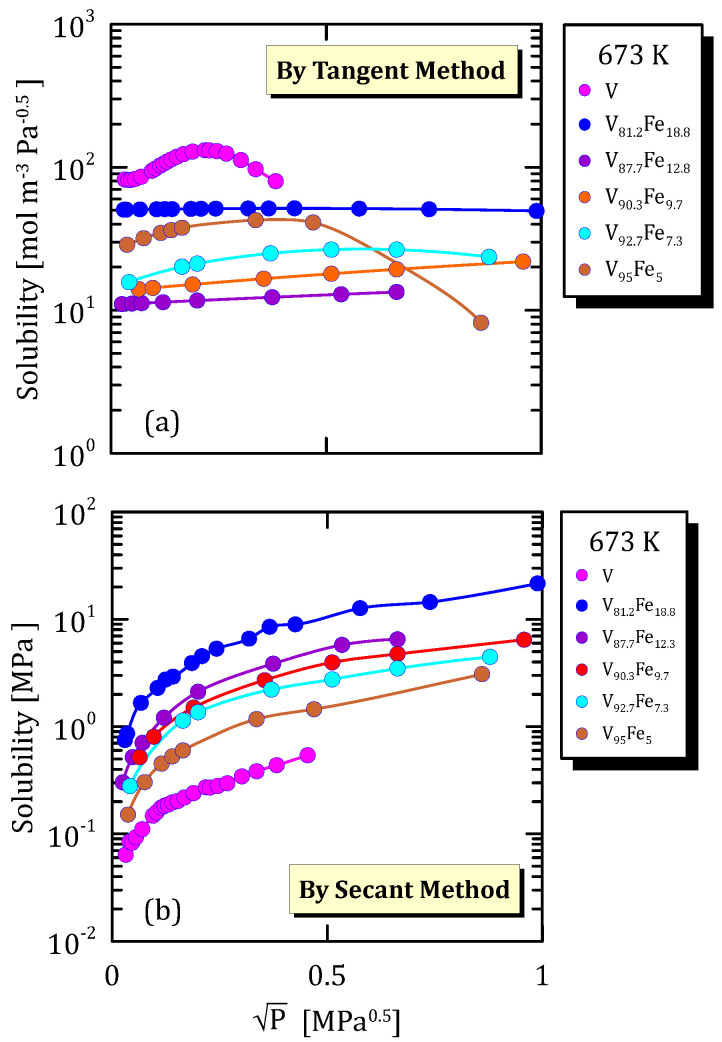
Solubility vs P^0.5^ of V alloys at 673 K [28,29] (See Table S8).

**Figure 14:**
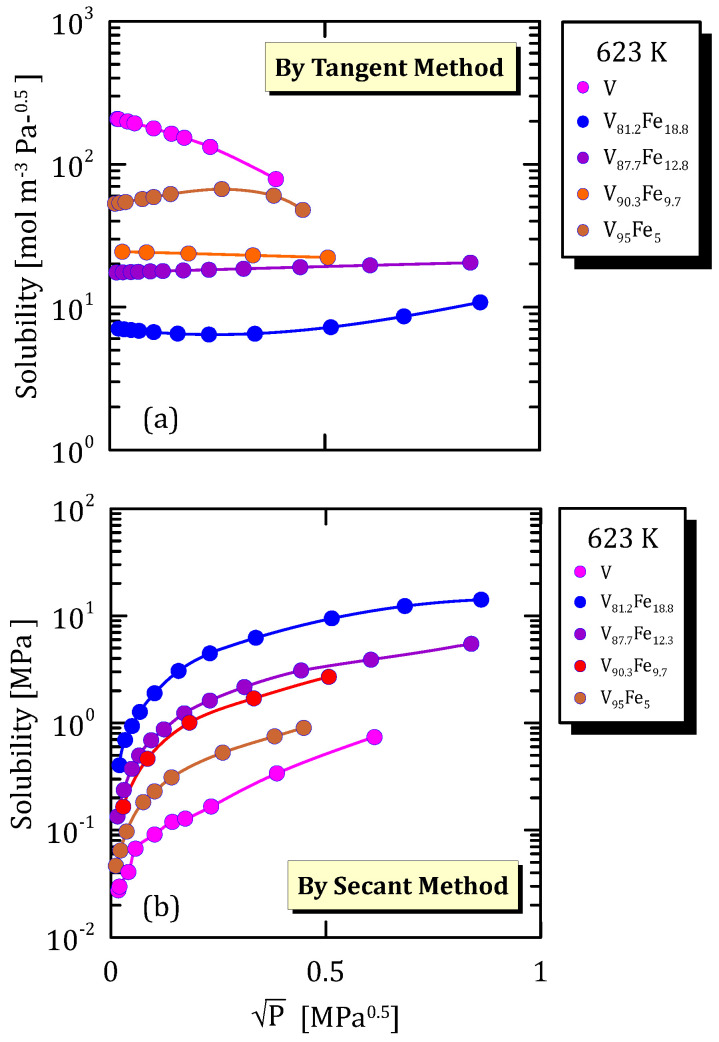
Solubility vs P^0.5^ of VFe alloys at 623 K [28,30] (see Table S11).

Figure 13a and Figure 14a highlights that, when vanadium is not alloyed or when its amount is greater than 95%, hydrogen solubility shows a maximum at 673 K. Solubility calculated through the secant method increases with pressure and reaches a plateau (Figure 13b and Figure 14b).

**Figure 15:**
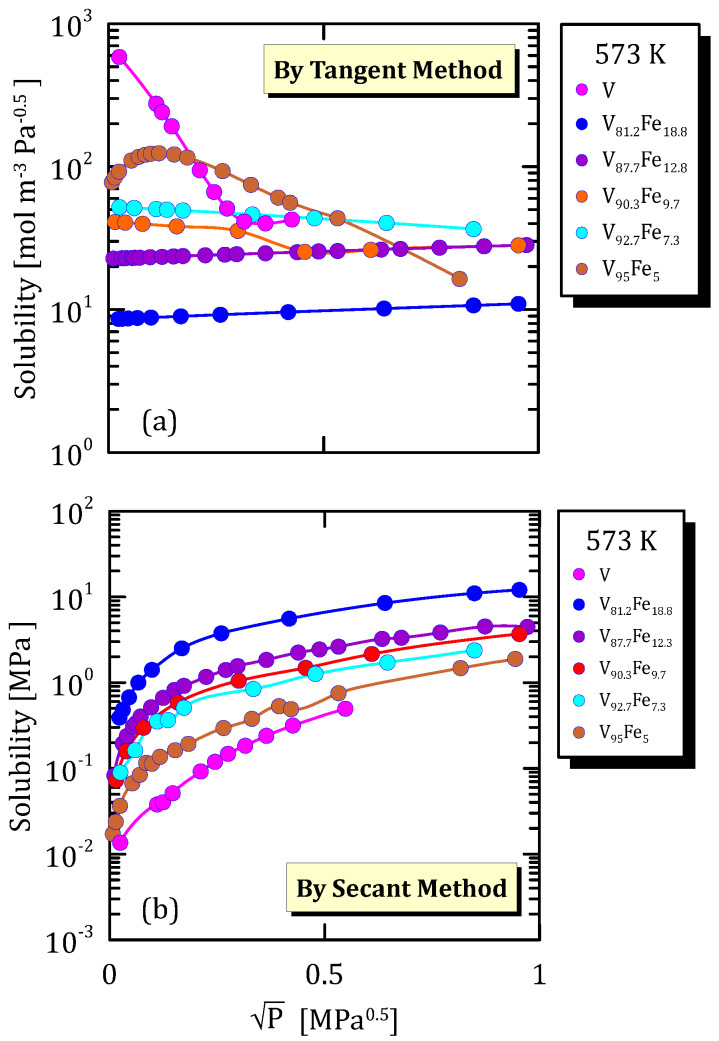
Solubility vs P^0.5^ of VFe alloys at 573 K [28,29] (see Table S12).

**Figure 16:**
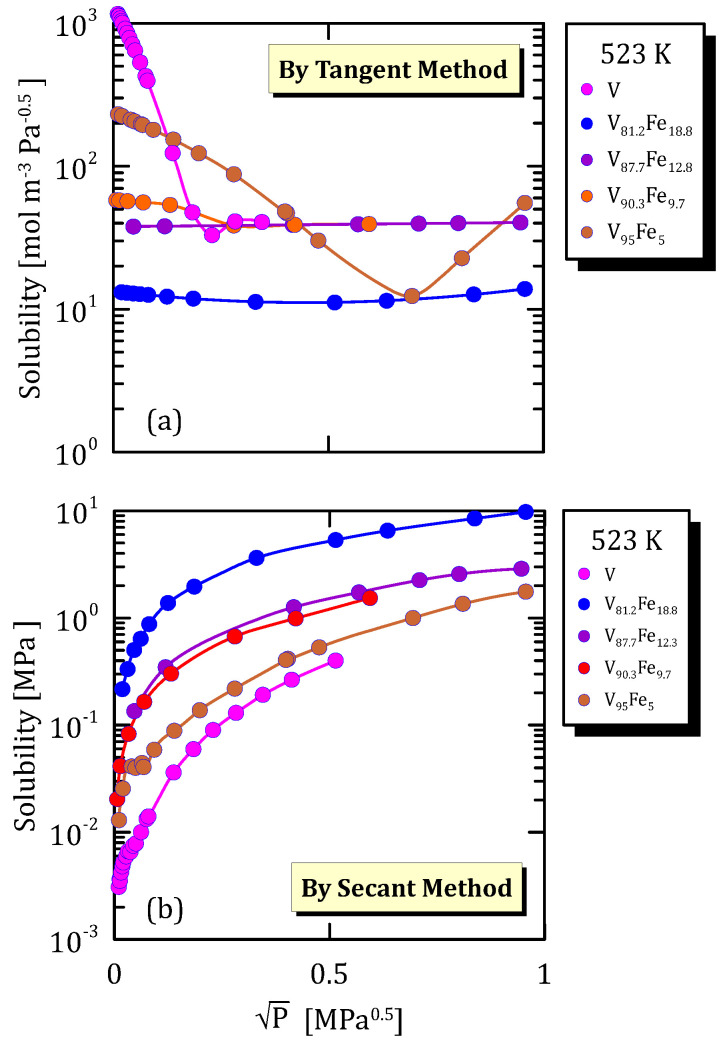
Solubility vs P^0.5^ of VFe alloys at 523 K [28,30]. (See Table S13).

**Figure 17:**
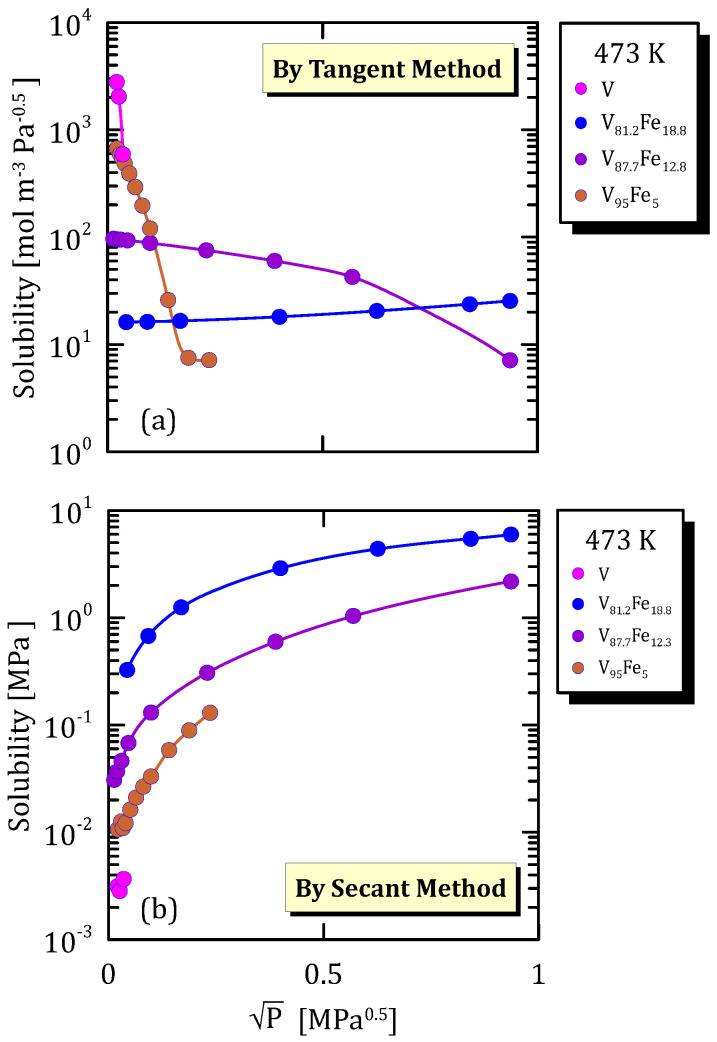
Solubility vs P^0.5^ of VFe alloys at 473 K [28,30] (see Table S14).

**Figure 18:**
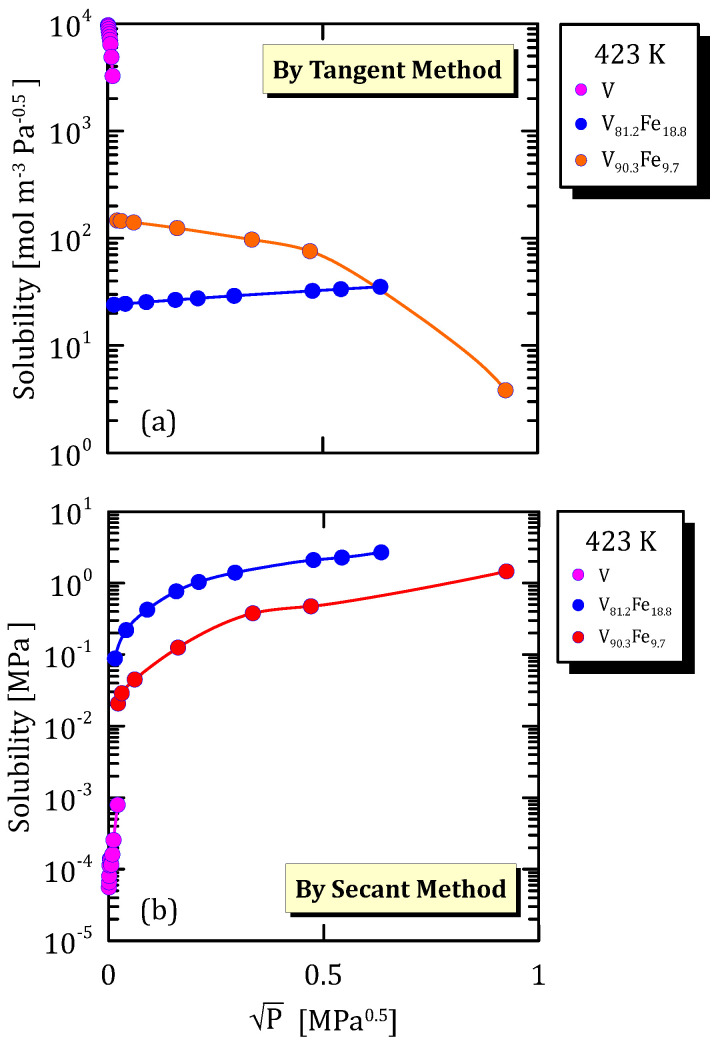
Solubility vs P^0.5^ of VFe alloys at 423 K [28,29] (see Table S15).

All the figures of the vanadium alloys at different temperature (Figure 15a, Figure 16a, Figure 17a and Figure 18a) show that hydrogen solubility (tangent method) in the alloy with the highest iron content has a monotone increasing trend. This indicates that a certain percentage of iron favours the hydrogen dissociative adsorption, thus increasing solubility with the square root partial pressure. Solubility calculated through the tangent method increases with pressure (Figure 15b, Figure 16b, Figure 17b and Figure 18b).

### 6.3. Nb-Based Membranes

In this section, the hydrogen solubility in Nb, NbRu, NbW and Nb_54_Ti_21_Co_25_ alloys is shown against the square root of hydrogen partial pressure at different temperature conditions and Ru contents. For the last alloy (Nb_54_Ti_21_Co_25_), we have converted the metal composition from the original at% to wt%.

**Figure 19:**
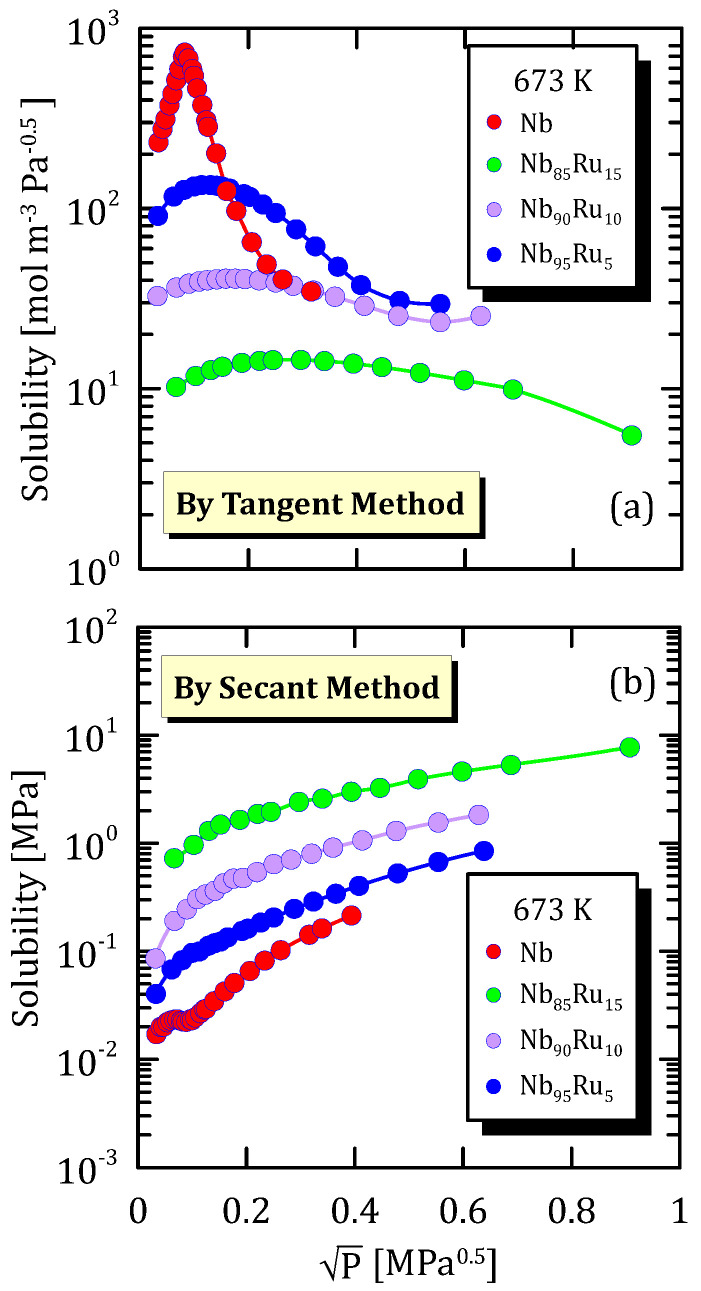
Solubility vs P^0.5^ of NbRu alloys at 673 K [31] (see Table S16).

Figure 19a shows a maximum, which is cause by two contrasting physical phenomena. On the one hand, the arise of pressure promotes hydrogen sorption, whereas, on the other hand, it leads to the reticular sites filling, which leads to a decrease of solubility.

**Figure 20:**
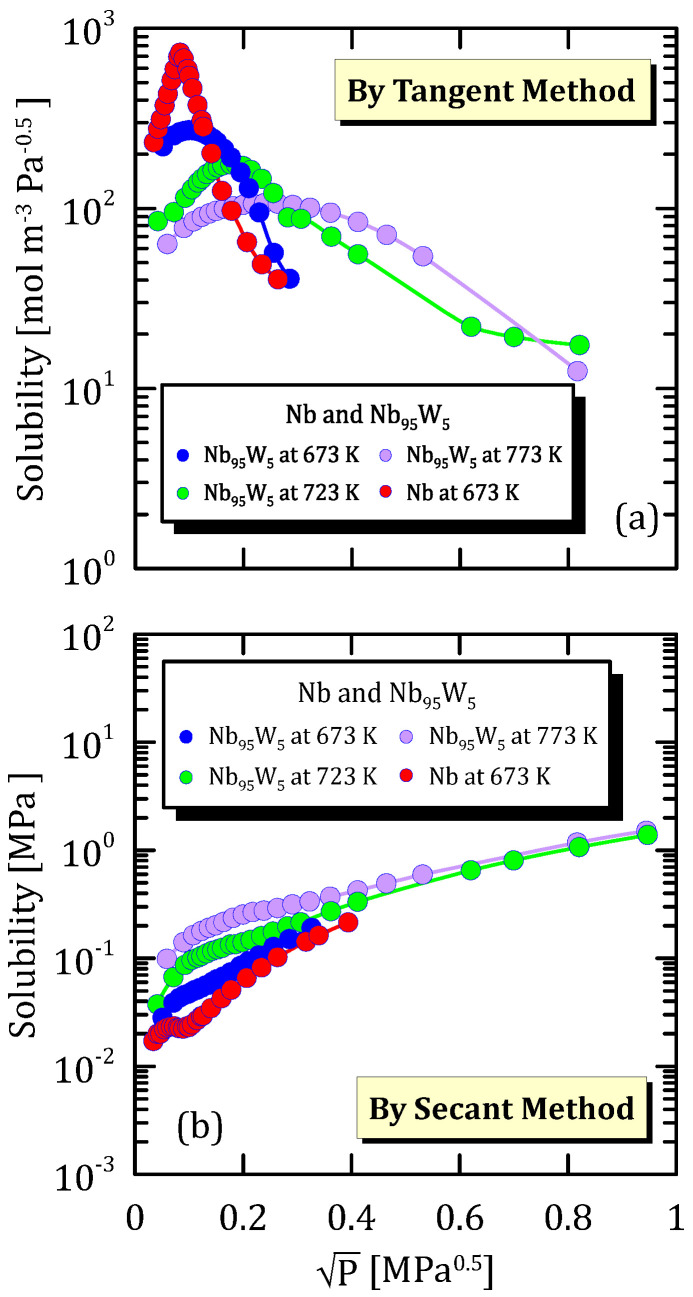
Solubility vs P^0.5^ of Nb and Nb_95_W_5_ at different temperatures [31] (see Table S17).

**Figure 21:**
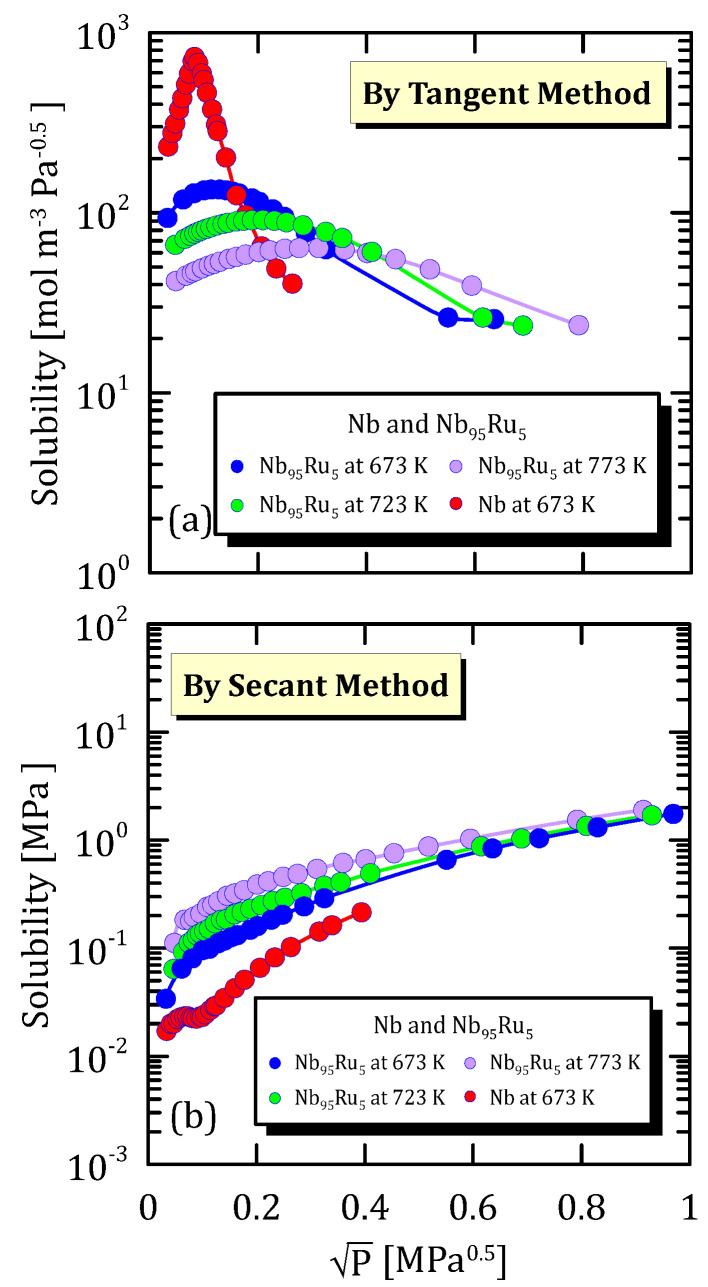
Solubility vs P^0.5^ of Nb and Nb_95_Ru_5_ at the different temperatures [31] (see Table S18).

The trend reported in Figure 19a is the same of the one in Figure 20a and Figure 21a. This confirms that the hydrogen solubility maximum depends not only on the alloy composition but also on temperature. However, this was to be expected since hydrogen shows a different solubility towards each metal and since the solubility is strongly affected by temperature.

Solubility calculated through the secant method increases with pressures and tends to reach a plateau (Figure 19b, Figure 20b and Figure 21b).

**Figure 22:**
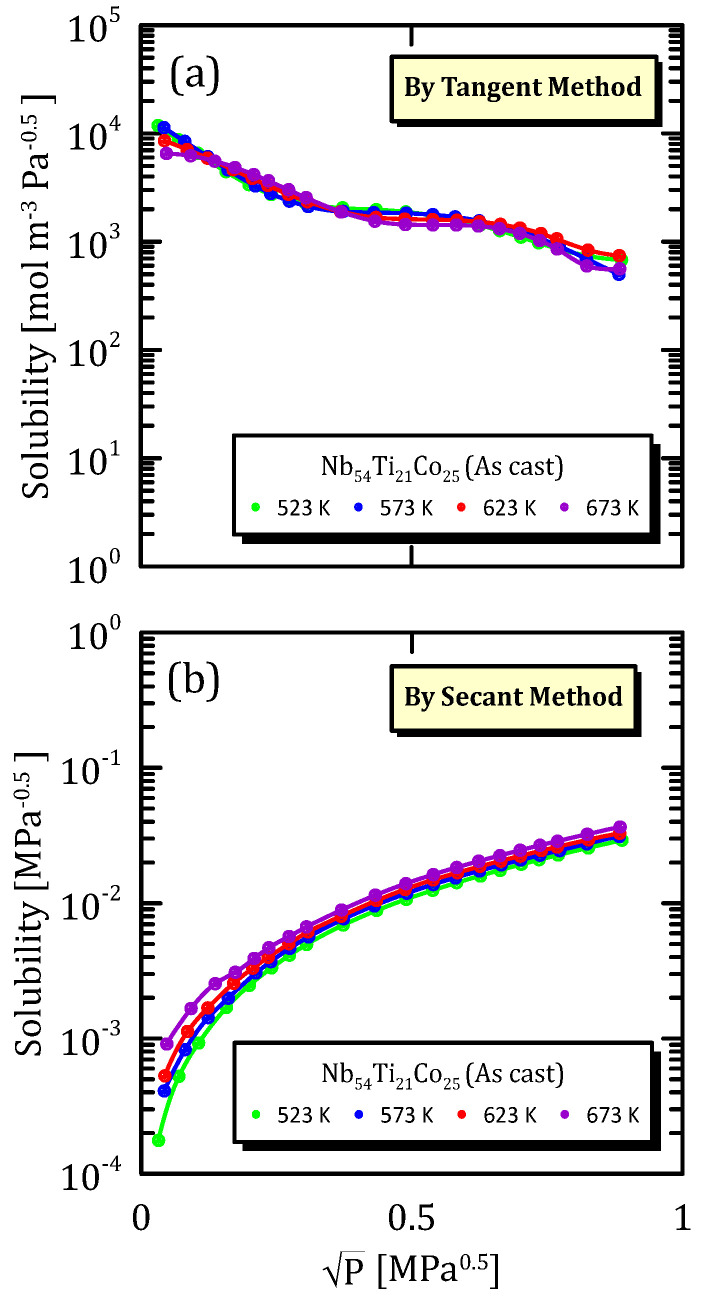
Solubility vs P^0.5^ of as-cast Nb_54_Ti_21_Co_25_ at the different temperatures [32].

**Figure 23:**
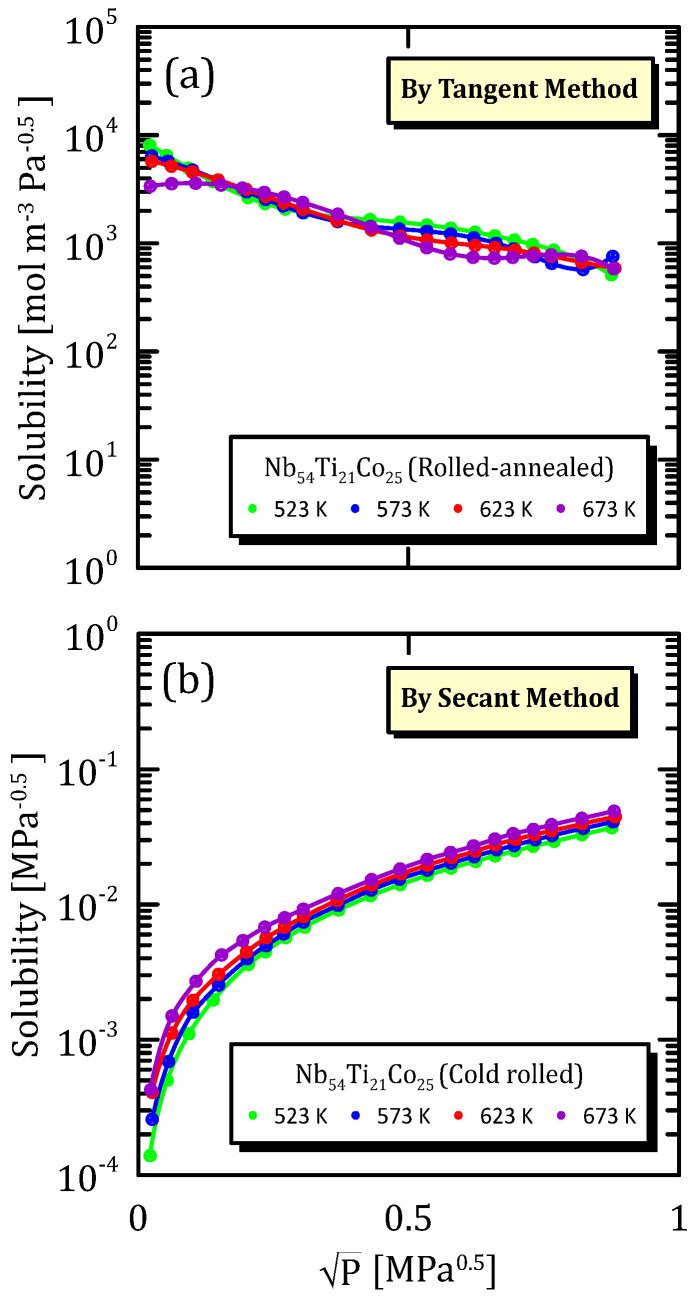
Solubility vs P^0.5^ of cold-rolled Nb_54_Ti_21_Co_25_ at the different temperatures [32].

**Figure 24:**
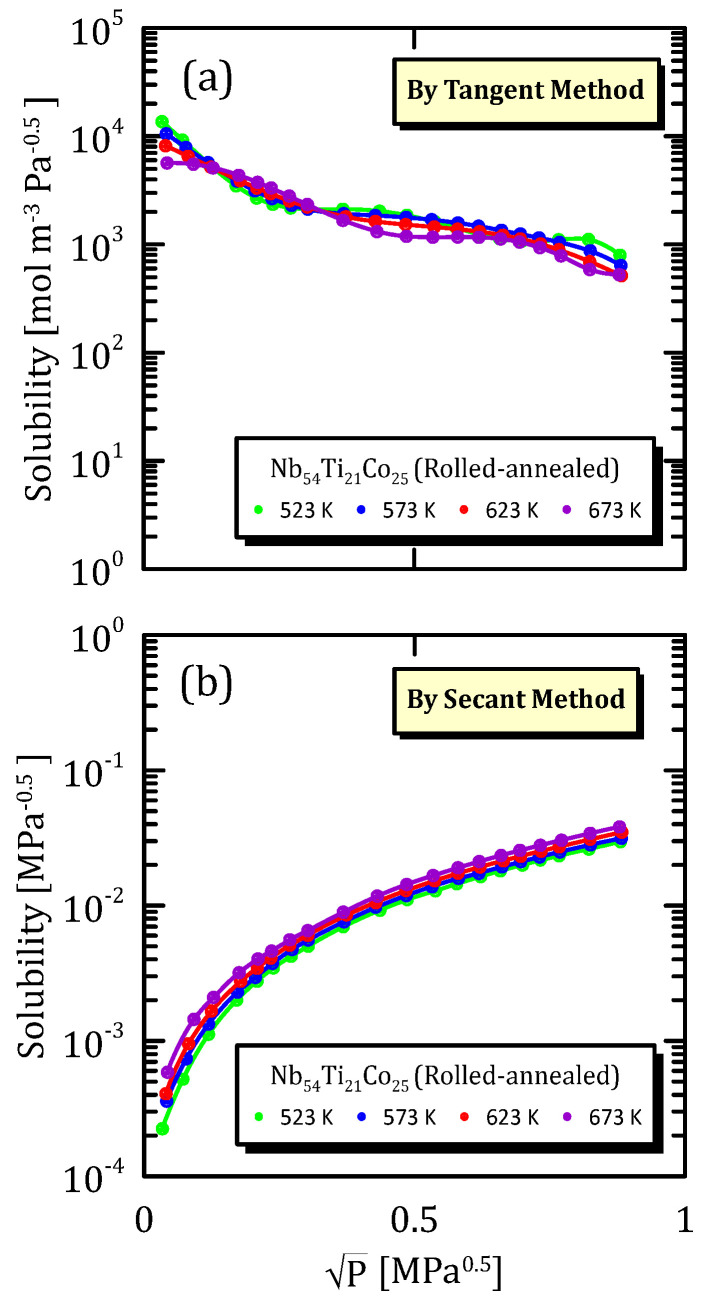
Solubility vs P^0.5^ of cold-rolled-annealed Nb_54_Ti_21_Co_25_ at the different temperatures [32].

Figure 22, Figure 23 and Figure 24 show the solubility data relative to a Nb alloy subjected to different mechanical modification techniques. In particular, Figure 22a,b show that the influence of temperature on solubility is not so important, and the same situation can be observed also in the other fabrication modifications (Figure 23a,b and Figure 24a,b).

The most interesting aspect to notice is that the cold rolled membranes show a higher solubility with respect to the other two cases, this indicating that the mechanical modification of the surface favour the hydrogen adsorption.

### 6.4. Ta-Based Membranes

The following data are organized according to the operating temperature and show how hydrogen solubility changes with the square root pressure for TaAl alloys with a different amount of Al.

**Figure 25:**
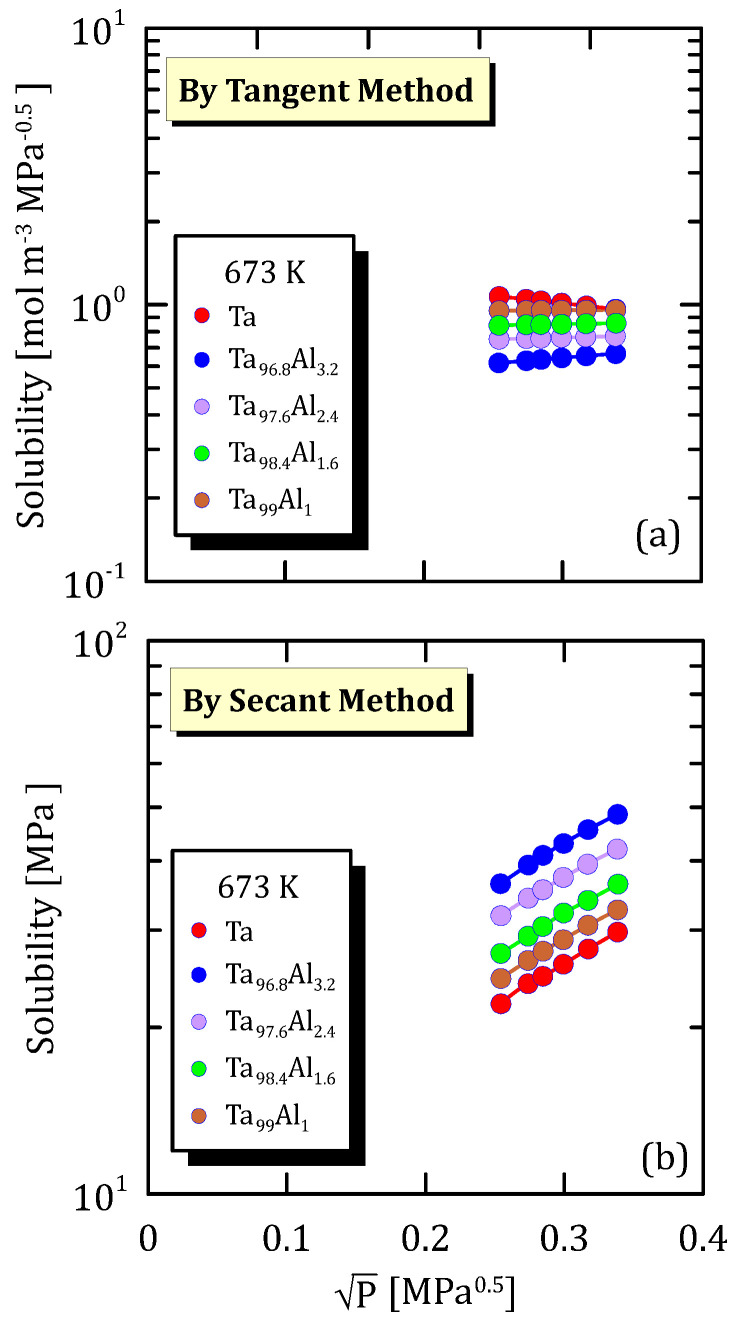
Solubility vs P^0.5^ of Ta and TaAl at 673 K [33] (see Table S19).

**Figure 26:**
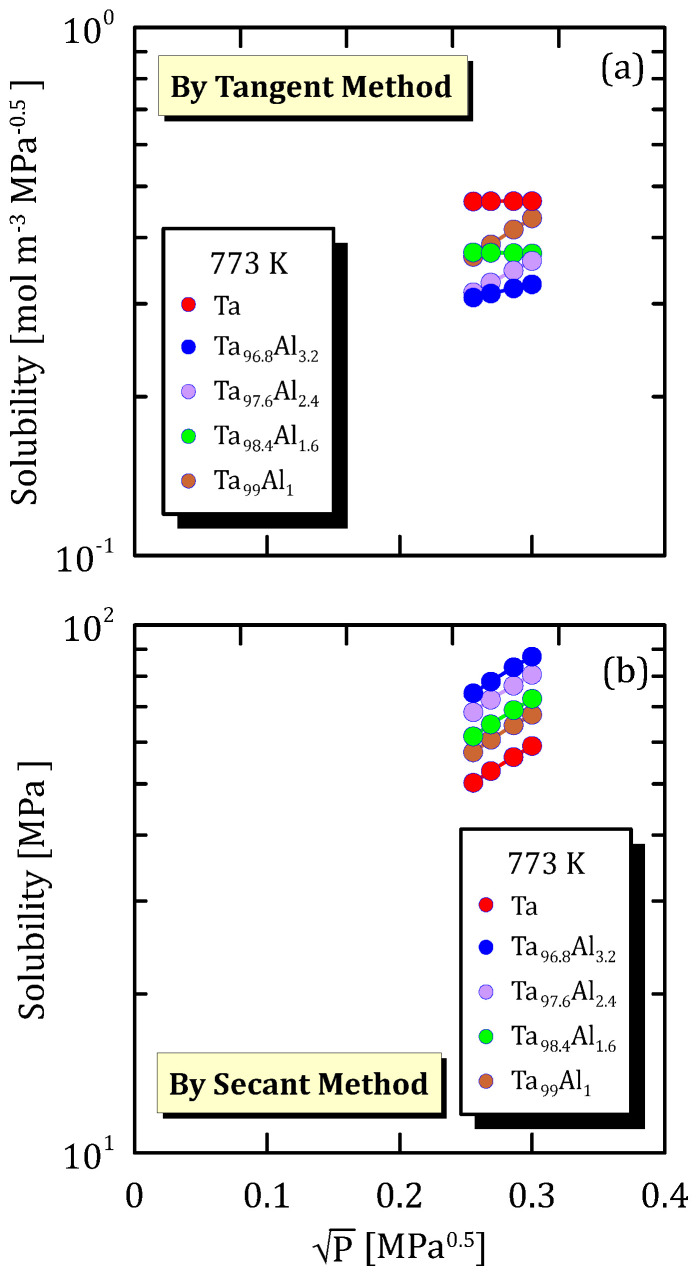
Solubility vs P^0.5^ of Ta and TaAl at 773 K [33] (see Table S20).

**Figure 27:**
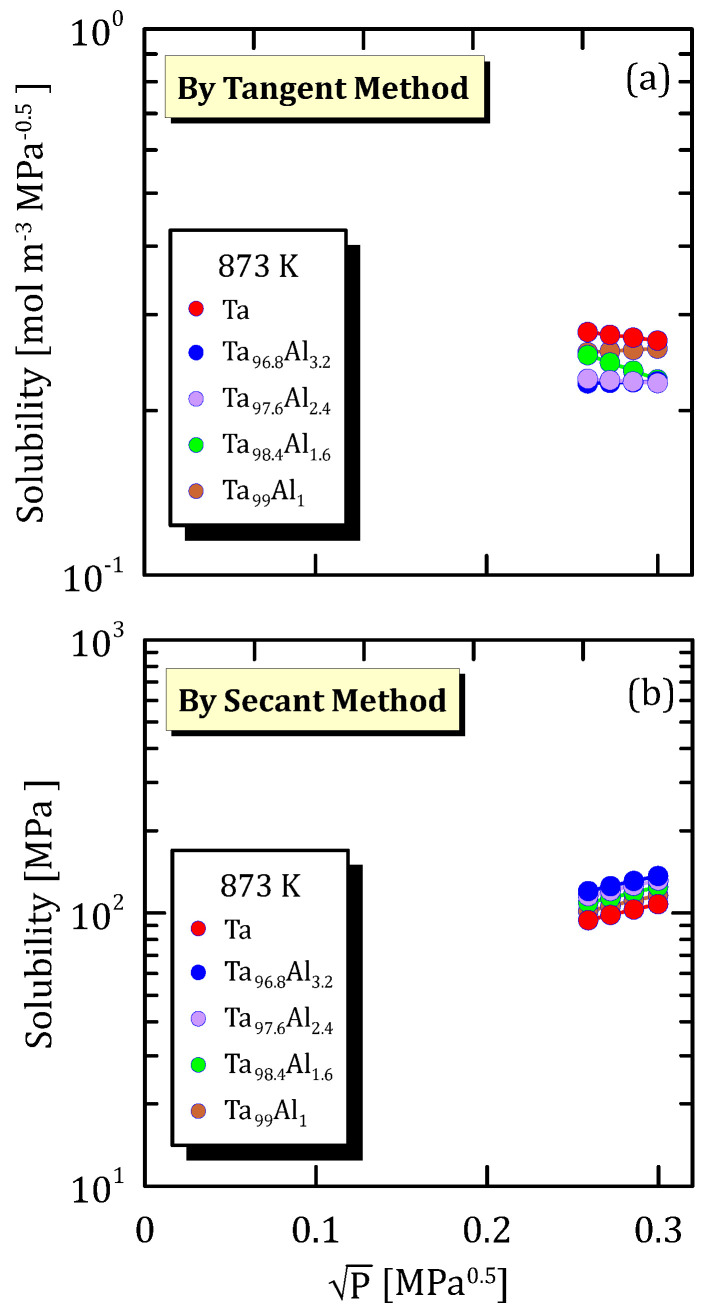
Solubility vs P^0.5^ of Ta and TaAl at 873 K [33] (see Table S21).

Figure 25a, Figure 26a and Figure 27a highlight that hydrogen solubility in Ta-based membranes calculated shows exactly an opposite trend than the one reported in Figure 25b, Figure 26b and Figure 27b, for the same reasons reported in Section 6.2. Moreover, it is clear that pure tantalum membranes show the highest hydrogen solubility (tangent method) compared to the alloyed ones.

### 6.5. Amorphous-Alloys Membranes

In the following figures, hydrogen solubility in amorphous alloys is represented against the square root of hydrogen partial pressure for different alloys compositions and temperatures.

**Figure 28:**
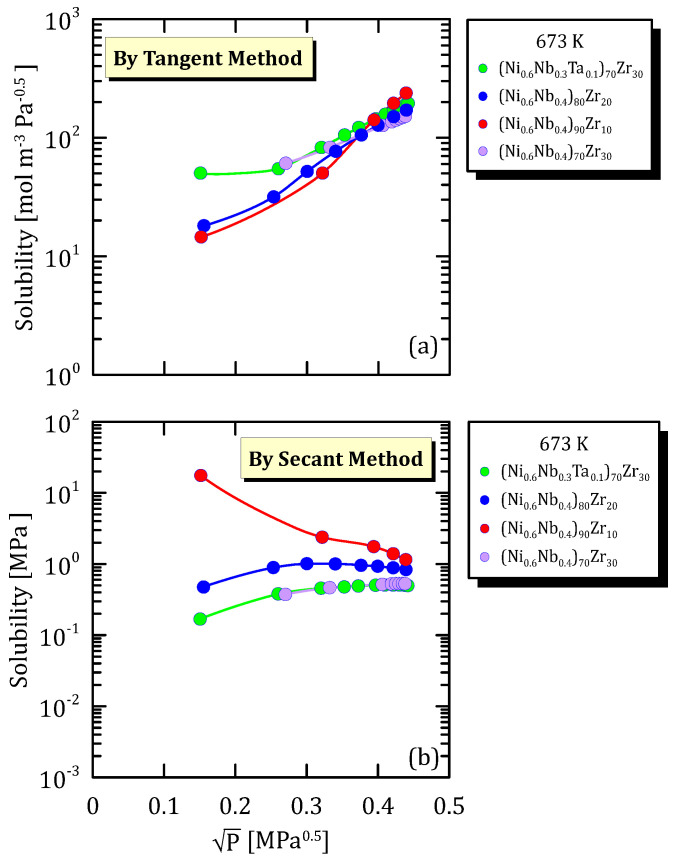
Solubility vs P^0.5^ of NiNbZr based alloys at 673.15 K [34] (see Table S23).

Figure 28a,b allows to understand that if solubility is calculated with tangent method, tantalum addition favours solubility, differently from what observed when calculating the solubility by secant method.

**Figure 29:**
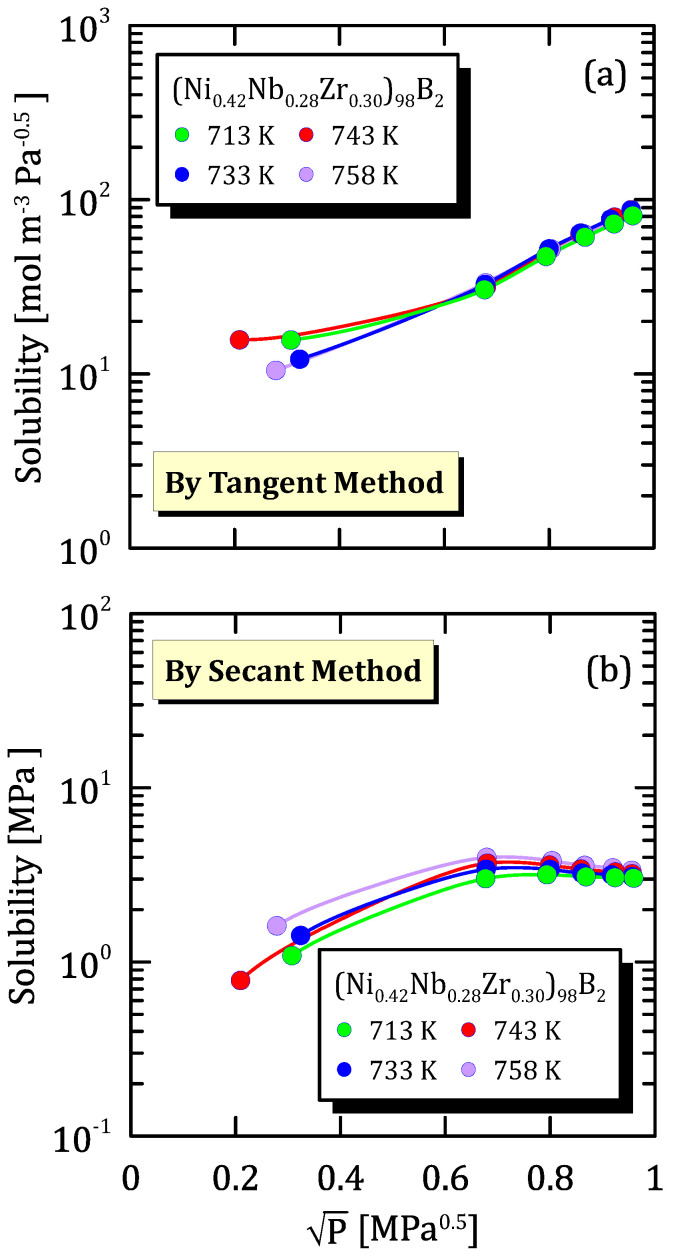
Solubility vs P^0.5^ of (Ni_0.42_Nb_0.28_Zr_0.30_)_98_B_2_ at different temperatures [34] (see Table S24).

In Figure 29a it is possible to notice an opposite behaviour of hydrogen solubility with temperature than the one showed in Figure 29b. However, both the figures point out that, at high pressure, solubility is almost independent of temperature. This can indicate that non-activated processes control the hydrogen dissolution in the metal lattice like, for example, morphology modifications due to high pressure.

**Figure 30:**
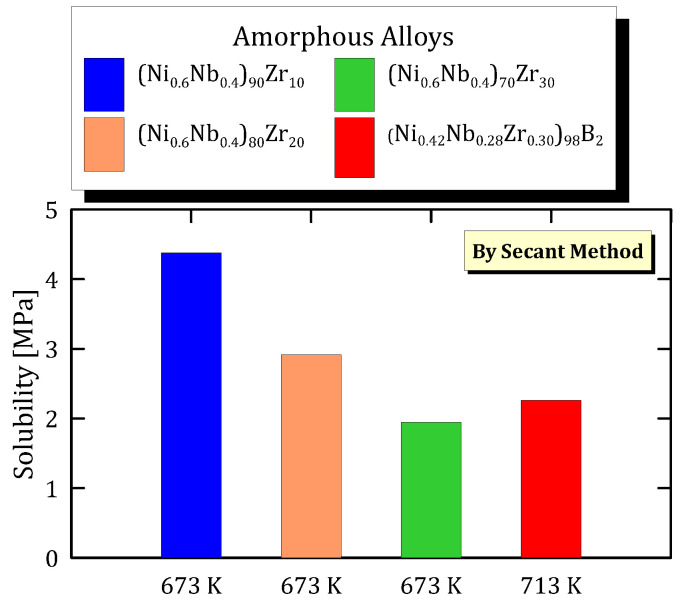
Solubility at 0.837 MPa^0.5^ of ZrNi based alloys at different temperatures [34,35] (see Table S25).

An increasing amount of Zr in the amorphous alloyed membranes disfavours hydrogen solubility. The presented data do not make it possible to analyse the effect of boron. This is due to the fact that the value of solubility for the B-containing alloy is available at a different temperature and, thus, is the combination of both temperature and composition effect (Figure 30).

### 6.6. Liquid-Gallium Membranes

**Figure 31:**
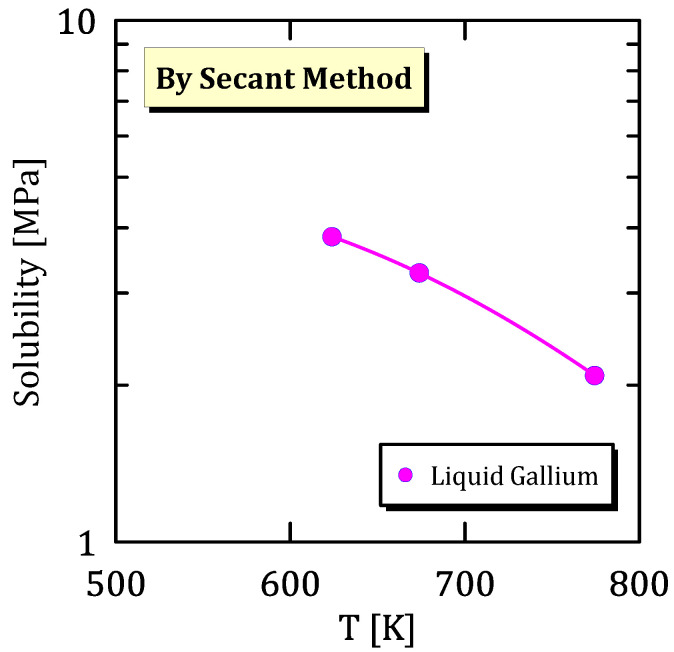
Solubility vs T of liquid gallium [36] (see Table S26).

This section contain data about hydrogen solubility in liquid gallium. In this case it was calculated only by secant method, because there is not a solid structure to take into account. Figure 31 shows that hydrogen solubility, in liquid gallium membranes, decreases as temperature increases.

Among all the materials investigated, the ternary alloy Nb-Ti-Co appears to exhibit the most favou able behaviour in terms of differential solubility under the tested operating conditions. However, solubility alone is not sufficient to determine the most suitable alloy for metal membrane fabrication. Furthermore, the choice of alloy should ultimately be guided by the specific requirements of the intended application.

## 7. Conclusions

This work was devoted to a comprehensive analysis of hydrogen solubility in the most common metal-alloys membranes: palladium-based, vanadium-based, niobium-based, tantalum-based, amorphous alloys and liquid gallium, highlighting advantages and disadvantages of each material. In particular, we started from the consideration that solubility in the literature is defined—and, thus, calculated—by two different ways, here referring to as: (i) “*secant method*”, consisting in calculating the solubility as the hydrogen pressure corresponding to a certain sorbed hydrogen loading, and (ii) “*tangent method*”, based on which differential solubility is evaluated as the derivative of the sorption isotherm in each point of the curve.

Subsequently, a number of experimental data were collected for several metal alloys, reprocessing them to evaluate the two types of solubilities. This enabled comparing and analysing the behaviour of the material investigated under different conditions of temperature, pressure and alloy composition.

The re-elaboration presented in this study helps identify the most suitable materials and membranes for hydrogen separation or storage, as well as helps recognise the maximum degree of embrittlement due to hydrogen content in the metal lattice.

Finally, the recent research has proven that promising membranes are represented by the “sandwich” one made of liquid gallium, which are not subjected to embrittlement and, at the same time, show higher performances.

## Supplementary Materials

The following supporting information can be downloaded at: https://www.mdpi.com/article/10.3390/membranes15090273/s1, Table S1: Pd77Ag33_T, Table S2: Pd70Ag30_T, Table S3: V97.5Fe2.5_T, Table S4: V92.5Fe7.5_T, Table S5: V90Fe10_T, Table S6: V85Ni15_T, Table S7: V90Ni10_T, Table S8: V95Ni5_T, Table S9: V95Ni5_T_Deu, Table S10: VFe_673K, Table S11: VFe_623K, Table S12: VFe_573K, Table S13: VFe_523K, Table S14: VFe_473K, Table S15: VFe_423K, Table S16: Nb_NbRu_673K, Table S17: Nb_NbW_T, Table S18: Nb_NbRu_T, Table S19: Ta_TaAl_673K, Table S20: Ta_TaAl_773K, Table S21: Ta_TaAl_873K, Table S22: Ta_TaAl_Sieverts,, Table S23: NiNbZr_673K, Table S24: (Ni0.42Nb0.28Zr0.30)98B2 _T, Table S25: ZrNi_T.

## List of Symbols

*J_H_*	Atomic hydrogen flux, mol_H_ m^−2^ s^−1^
*S_H_*	Solubility of H specie, mol_H_ m^−3^ MPa^−0.5^
*D_H_*	Diffusion coefficient of H specie, m^2^ s^−1^
*a*	Activity of species, -
${\overset{̿}{C}}_{H}$	Concentration of H specie in the metal phase, mol m_Pd_^−3^
${\overset{̿}{\xi }}_{H}$	H occupancy degree into the metal bulk, mol_H_ mol_Pd_^−1^
$P_{H_{2}}$	Partial pressure of H_2_ specie, Pa
${\bar{\theta }}_{H}$	Surface coverage degree of H, -
${\overset{̿}{H}}_{H}$	Sieverts’ (Henry’s) constant, Pa^−0.5^
γ	Activity coefficient, -
${\bar{C}}_{H}$	Concentration of H specie in the metal phase, mol_H_ m_Pd_^−2^
$\phi_{H_{2}}$	Fugacity coefficient of H_2_ specie, -
*x*	Spatial coordinate, m
*K_j_*	Equilibrium constant of the jth adsorption/absorption reaction, -

## References

[1] Say K. (2025). Moving beyond peak oil: The importance of renewable energy in the sustainability transition. The Routledge Handbook of Global Sustainability Education and Thinking for the 21st Century.

[2] Guo Y., Yang Y., Bradshaw M., Wang C., Blondeel M. (2023). Globalization and Decarbonization: Changing Strategies of Global Oil and Gas Companies.

[3] Ahad M.T., Bhuiyan M.M.H., Sakib A.N., Corral A.B., Siddique Z. (2023). An overview of challenges for the future of hydrogen. Materials.

[4] McCay M.H., Shafiee S. (2020). Hydrogen: An energy carrier. Future Energy: Improved, Sustainable and Clean Options for Our Planet.

[5] Agyekum E.B., Nutakor C., Agwa A.M., Kamel S. (2022). A critical review of renewable hydrogen production methods: Factors affecting their scale-up and its role in future energy generation. Membranes.

[6] Kim Y., Yang H. (2025). Hydrogen Purity: Influence of Production Methods, Purification Techniques, and Analytical Approaches. Energies.

[7] Dash S.K., Chakraborty S., Elangovan D. (2023). A brief review of hydrogen production methods and their challenges. Energies.

[8] Liu C., Zhang X., Zhai J., Li X., Guo X., He G. (2023). Research Progress and Prospects on Hydrogen Separation Membranes.

[9] Zito P.F., Prenesti G., Caravella A. (2024). H_2_ and CO purification by CO_2_ removal from syngas coupling membrane module and water-absorption unit: An economic analysis. Int. J. Hydrogen Energy.

[10] Caravella A., Prenesti G., Martinez-Diaz D., Alique D., Hara S. (2024). Optimal Permeance Ratio, Flux Direction and Layer Distribution in Composite Asymmetric Membranes composed of Sequences of Layers obeying Real-Power Flux Laws. J. Membr. Sci..

[11] Caravella A., Martinez-Diaz D., Prenesti GMichienzi V., Calles J.A., Sanz R., Alique D. (2023). Effect of Flux Direction through Supported Metal Membranes: Golden Ratio as Maximum Benefit in Pure Hydrogen and Concept of Swap Point in Mixture. J. Membr. Sci..

[12] Martinez-Diaz D., Michienzi V., Calles J.A., Sanz R., Caravella A., Alique D. (2022). Versatile and Resistant Electroless Pore-Plated Pd-Membranes for H2-Separation: Morphology and Performance of Internal Layers in PSS Tubes. Membranes.

[13] Zhao C., Caravella A., Xu H., Brunetti A., Barbieri G., Goldbach A. (2018). Support mass transfer resistance of Pd/ceramic composite membranes in the presence of sweep gas. J. Membr. Sci..

[14] Mulder M. (1996). Basic Principles of Membrane Technology.

[15] Mikolajick T., Galderisi G., Rai S., Simon M., Bockle R., Sistani M., Cakirlar C., Bhattacharjee N., Mauersberger T., Heinzig A. (2022). Reconfigurable field effect transistors: A technology enablers perspective. Solid-State Electron..

[16] Caravella A., Scura F., Barbieri G., Drioli E. (2010). Sieverts Law Empirical Exponent for Pd-Based Membranes: Critical Analysis in Pure H_2_ Permeation. J. Phys. Chem. B.

[17] Yun S., Oyama S.T. (2011). Correlations in palladium membranes for hydrogen separation: A review. J. Membr. Sci..

[18] Caravella A., Barbieri G., Drioli E. (2008). Modelling and simulation of hydrogen permeation through supported Pd-alloy membranes with a multicomponent approach. Chem. Eng. Sci..

[19] Yang B., Gu Y., Guo J., Shi X., Li Y., Zhang S., Song G. (2025). Significant improvement of cold-rolling formability and hydrogen embrittlement resistance of Y-doped V alloy membranes for hydrogen separation. J. Membr. Sci..

[20] Alique D., Martinez-Diaz D., Sanz R., Calles A. (2018). Review of Supported Pd-Based Membranes Preparation by Electroless Plating for Ultra-Pure Hydrogen Production. Membranes.

[21] Ward T., Dao T. (1999). Model of hydrogen permeation behavior in palladium membranes. J. Membr. Sci..

[22] Wijmans J.G., Baker R.W. (1995). The solution-diffusion model: A review. J. Membr. Sci..

[23] Hara S., Caravella A., Ishitsuka M., Suda H., Mukaida M., Haraya K., Shimano E., Tsuji T. (2012). Hydrogen diffusion coefficient and mobility in palladium as a function of equilibrium pressure evaluated by permeation measurement. J. Membr. Sci..

[24] Paolone A., Tosti S., Santucci A., Palumbo Trequattrini F. (2017). Hydrogen and deuterium solubility in commercial Pd-Ag alloys for hydrogen purification. Chemengineering.

[25] Suzuki A., Yukawa H., Nambu T., Matsumoto Y., Murata Y. (2016). Quantitative Evaluation of Hydrogen Solubility and Diffusivity of V-Fe Alloys toward the Design of Hydrogen Permeable Membrane for Low Operative Temperature. Mater. Trans..

[26] Dolan M.D., McLennan K.G., Way J.W. (2012). Diffusion of atomic hydrogen through V-Ni alloy membranes under nondilute conditions. J. Phys. Chem..

[27] Brutti S., Tosti S., Santucci A., Paolone A. (2019). Deuterium absorption properties of V_85_Ni_15_ and evidence of isotope effect. Int. J. Hydrogen Energy.

[28] Alimov V.N., Busnyuk A.O., Notkin M.E., Peredistov E.Y., Livshits A.I. (2004). Substitutional VePd alloys for the membranes permeable to hydrogen: Hydrogen solubility at 150 and 400 °C. Int. J. Hydrogen Energy.

[29] Fromm E., Gebhardt E. (1976). Gase und Kohlenstoff in Metallen.

[30] Schober T. (1996). Vanadium-, niobium- and tantalum-hydrogen. Solid State Phenom..

[31] Yukawa Y., Nambu T., Matsumoto Y., Watanabe N., Zhang G., Morinaga M. (2008). Alloy Design of Nb-Based Hydrogen Permeable Membrane with Strong Resistance to Hydrogen Embrittlement. Mater. Trans..

[32] Li X., Liu D., Liang X., Chen R., Rettenmayr M., Su Y., Guo J., Fu H. (2016). Hydrogen transport behaviour of as-cast, cold rolled and annealed Nb_40_Ti_30_Co_30_ alloy membranes. J. Membr. Sci..

[33] Taxak M., Kumar S., Krishnamurthy N. (2016). Thermodynamic Parameters for the Solubility of Hydrogen in Tantalum-Aluminium Alloys. Innov. Energy Res..

[34] Palumbo O., Trequattrini F., Sarker S., Hulyakar M., Pal N., Chandra D., Dolan M., Paolone A. (2017). New Studies of the Physical Properties of Metallic Amorphous Membranes for Hydrogen Purification. Challenges.

[35] Palumbo O., Trequattrini F., Pal N., Hulyalkar M., Sarker S., Chandra D., Flanagan t pnichk M., Paolone A. (2017). Hydrogen absorption properties of amorphous (Ni_0.6_Nb_0.4−y_Ta_y_)_100−x_Zr_x_ membranes. Prog. Nat. Sci. Mater. Int..

[36] Yen P.S., Deveau N.D., Datta R. (2017). Sandwiched liquid metal membrane (SLiMM) for hydrogen purification. AIChE.

