# Supplementary material for: Hydrogen Solubility in Metal Membranes: Critical Review and Re-Elaboration of Literature Data
Source: Membranes (Basel). 2025 Sep 9;15(9):273. doi: 10.3390/membranes15090273 (PMC12472185; doi:10.3390/membranes15090273)
Supplement: Supplementary file 1 [file membranes-15-00273-s001.zip › membranes-3792789-supplementary.pdf]

Hydrogen Solubility in Metal Membranes.
Re-elaboration and Critical Analysis of Literature Data

Giuseppe PRENESTI [1,2]*, Alessia ANOJA [1], Pierfrancesco PERRI [3], Abdulrahman Yaqoub ALRAEESI [4], Shigeki HARA [5] and Alessio CARAVELLA [1,2]

[1] Department of Computer Engineering, Modelling, Electronics and Systems Engineering (DIMES), University of Calabria (UNICAL), Via P. Bucci, Cubo 42C, Rende (CS), 87036, ITALY
[2] Institute on Membrane Technology, National Research Council (CNR-ITM), Via P. Bucci, Cubo 17C, Rende (CS), 87036, ITALY
[3] Department of Civil, Chemical, Environmental and Materials Engineering (DICAM), Alma Mater Studiorum University of Bologna, Via Zamboni 33, 40126 Bologna (BO), ITALY
[4] Chemical and Petroleum Engineering Department, College of Engineering, United Arab Emirates University, P.O. Box 15551, United Arab Emirates
[5] Nanomaterials Research Institute, National Institute of Advanced Industrial Science and Technology (AIST), Higashi 1-1-1, Tsukuba, 305-8565, JAPAN

* Correspondence: giuseppe.prenesti@unical.it

Supplemental Material: Tables of data

The data tables in the following sections report solubility data calculated by the tangent and the secant method. The former is expressed in mol·m^{-3}·MPa$^{-0.5}$, whereas the latter in MPa$^{0.5}$.

B.1 Solubility data

B.1.1 Pd based membranes

Table S1 $Pd_{77}Ag_{23}$ solubility at different temperatures.

Reference	Alloy composition (wt%)	Thickness (μm)	Preparation method	T (K)	Hydrogen content (H/M)	Solubility (MPa)	$P^{0.5}$ ($MPa^{0.5}$)	Solubility (mol·m^{-3}·MPa$^{-0.5}$)
[1]	$Pd_{77}Ag_{23}$	25	Metallurgical	298.15	0.620	0.091	0.237	48403.744
[1]	$Pd_{77}Ag_{23}$	25	Metallurgical	298.15	0.690	0.312	0.464	24754.670
[1]	$Pd_{77}Ag_{23}$	25	Metallurgical	298.15	0.752	0.913	0.828	13873.126
[1]	$Pd_{77}Ag_{23}$	25	Metallurgical	367.15	0.163	0.103	0.130	496392.681
[1]	$Pd_{77}Ag_{23}$	25	Metallurgical	367.15	0.247	0.086	0.146	482629.078
[1]	$Pd_{77}Ag_{23}$	25	Metallurgical	367.15	0.345	0.086	0.172	390377.701
[1]	$Pd_{77}Ag_{23}$	25	Metallurgical	367.15	0.437	0.098	0.207	139022.780

[1]	$Pd_{77}Ag_{23}$	25	Metallurgical	367.15	0.529	0.123	0.255	57263.777
[1]	$Pd_{77}Ag_{23}$	25	Metallurgical	367.15	0.584	0.200	0.341	42810.025
[1]	$Pd_{77}Ag_{23}$	25	Metallurgical	367.15	0.640	0.514	0.574	25477.684
[1]	$Pd_{77}Ag_{23}$	25	Metallurgical	393.15	0.127	0.219	0.167	182728.337
[1]	$Pd_{77}Ag_{23}$	25	Metallurgical	393.15	0.206	0.231	0.218	214824.995
[1]	$Pd_{77}Ag_{23}$	25	Metallurgical	393.15	0.328	0.220	0.269	226836.674
[1]	$Pd_{77}Ag_{23}$	25	Metallurgical	393.15	0.452	0.247	0.334	213673.420
[1]	$Pd_{77}Ag_{23}$	25	Metallurgical	393.15	0.538	0.355	0.437	66883.805
[1]	$Pd_{77}Ag_{23}$	25	Metallurgical	393.15	0.599	0.652	0.625	10137.063
[1]	$Pd_{77}Ag_{23}$	25	Metallurgical	423.15	0.047	0.309	0.120	59531.737
[1]	$Pd_{77}Ag_{23}$	25	Metallurgical	423.15	0.114	0.483	0.234	103553.812
[1]	$Pd_{77}Ag_{23}$	25	Metallurgical	423.15	0.184	0.539	0.315	119954.056
[1]	$Pd_{77}Ag_{23}$	25	Metallurgical	423.15	0.298	0.549	0.404	124247.010
[1]	$Pd_{77}Ag_{23}$	25	Metallurgical	423.15	0.428	0.605	0.509	110341.420
[1]	$Pd_{77}Ag_{23}$	25	Metallurgical	423.15	0.556	0.972	0.735	11129.148
[1]	$Pd_{77}Ag_{23}$	25	Metallurgical	439.15	0.038	0.516	0.140	41355.115
[1]	$Pd_{77}Ag_{23}$	25	Metallurgical	439.15	0.112	0.841	0.307	83348.150
[1]	$Pd_{77}Ag_{23}$	25	Metallurgical	439.15	0.167	0.876	0.383	93228.859

[1]	$Pd_{77}Ag_{23}$	25	Metallurgical	439.15	0.356	0.941	0.579	92022.002
[1]	$Pd_{77}Ag_{23}$	25	Metallurgical	439.15	0.440	1.053	0.680	76329.026
[1]	$Pd_{77}Ag_{23}$	25	Metallurgical	439.15	0.518	1.465	0.871	18888.257
[1]	$Pd_{77}Ag_{23}$	25	Metallurgical	457.15	0.033	0.593	0.139	20056.598
[1]	$Pd_{77}Ag_{23}$	25	Metallurgical	457.15	0.074	1.166	0.294	32807.956
[1]	$Pd_{77}Ag_{23}$	25	Metallurgical	457.15	0.138	1.452	0.448	62487.361
[1]	$Pd_{77}Ag_{23}$	25	Metallurgical	457.15	0.211	1.544	0.571	82294.165
[1]	$Pd_{77}Ag_{23}$	25	Metallurgical	457.15	0.293	1.465	0.655	86942.421
[1]	$Pd_{77}Ag_{23}$	25	Metallurgical	457.15	0.366	1.579	0.760	75972.598
[1]	$Pd_{77}Ag_{23}$	25	Metallurgical	457.15	0.431	1.826	0.887	27696.529
[1]	$Pd_{77}Ag_{23}$	25	Metallurgical	473.15	0.027	0.808	0.148	21702.822
[1]	$Pd_{77}Ag_{23}$	25	Metallurgical	473.15	0.059	1.441	0.291	27789.396
[1]	$Pd_{77}Ag_{23}$	25	Metallurgical	473.15	0.096	1.913	0.428	35104.133
[1]	$Pd_{77}Ag_{23}$	25	Metallurgical	473.15	0.155	2.145	0.577	44811.633
[1]	$Pd_{77}Ag_{23}$	25	Metallurgical	473.15	0.216	2.379	0.717	55539.663
[1]	$Pd_{77}Ag_{23}$	25	Metallurgical	473.15	0.285	2.466	0.838	66165.610
[1]	$Pd_{77}Ag_{23}$	25	Metallurgical	493.15	0.021	1.218	0.162	17399.134
[1]	$Pd_{77}Ag_{23}$	25	Metallurgical	493.15	0.051	2.003	0.320	23870.759

[1]	$Pd_{77}Ag_{23}$	25	Metallurgical	493.15	0.110	2.725	0.548	33161.685
[1]	$Pd_{77}Ag_{23}$	25	Metallurgical	493.15	0.179	3.114	0.748	41333.485
[1]	$Pd_{77}Ag_{23}$	25	Metallurgical	519.15	0.022	1.508	0.184	1586.992
[1]	$Pd_{77}Ag_{23}$	25	Metallurgical	519.15	0.051	2.546	0.359	1593.274
[1]	$Pd_{77}Ag_{23}$	25	Metallurgical	519.15	0.085	3.625	0.554	1620.307
[1]	$Pd_{77}Ag_{23}$	25	Metallurgical	519.15	0.131	4.458	0.764	1672.875
[1]	$Pd_{77}Ag_{23}$	25	Metallurgical	556.15	0.015	2.305	0.187	49.485
[1]	$Pd_{77}Ag_{23}$	25	Metallurgical	556.15	0.029	3.469	0.318	141.374
[1]	$Pd_{77}Ag_{23}$	25	Metallurgical	556.15	0.061	4.990	0.550	300.311
[1]	$Pd_{77}Ag_{23}$	25	Metallurgical	556.15	0.091	6.448	0.764	441.850
[1]	$Pd_{77}Ag_{23}$	25	Metallurgical	573.15	0.012	3.103	0.196	9211.146
[1]	$Pd_{77}Ag_{23}$	25	Metallurgical	573.15	0.023	4.028	0.307	11012.025
[1]	$Pd_{77}Ag_{23}$	25	Metallurgical	573.15	0.047	5.826	0.523	12777.345
[1]	$Pd_{77}Ag_{23}$	25	Metallurgical	573.15	0.079	8.104	0.800	11660.176
[1]	$Pd_{77}Ag_{23}$	25	Metallurgical	623.15	0.007	4.559	0.178	5106.666
[1]	$Pd_{77}Ag_{23}$	25	Metallurgical	623.15	0.016	6.600	0.324	6101.627
[1]	$Pd_{77}Ag_{23}$	25	Metallurgical	623.15	0.030	10.306	0.552	8175.948
[1]	$Pd_{77}Ag_{23}$	25	Metallurgical	623.15	0.048	12.023	0.757	10584.181

[1]	$Pd_{77}Ag_{23}$	25	Metallurgical	673.15	0.009	11.744	0.331	3667.007
[1]	$Pd_{77}Ag_{23}$	25	Metallurgical	673.15	0.017	16.262	0.531	4259.269
[1]	$Pd_{77}Ag_{23}$	25	Metallurgical	673.15	0.026	21.727	0.757	4926.668

Table S2 Pd30Ag70 solubility at different temperatures.

Reference	Alloy composition (wt%)	Thickness (μm)	Preparation method	T (K)	Hydrogen content (H/M)	Solubility (MPa)	$P^{0.5}$ ($MPa^{0.5}$)	Solubility ($mol \cdot m^{-3} \cdot MPa^{-0.5}$)
[1]	$Pd_{30}Ag_{70}$	150	Metallurgical	463.15	0.002	40.326	0.253	3.526
[1]	$Pd_{30}Ag_{70}$	150	Metallurgical	463.15	0.006	72.881	0.639	3.526
[1]	$Pd_{30}Ag_{70}$	150	Metallurgical	513.15	0.001	47.456	0.258	3.588
[1]	$Pd_{30}Ag_{70}$	150	Metallurgical	513.15	0.005	83.371	0.649	3.588
[1]	$Pd_{30}Ag_{70}$	150	Metallurgical	573.15	0.001	62.294	0.304	2.591
[1]	$Pd_{30}Ag_{70}$	150	Metallurgical	573.15	0.004	91.675	0.594	2.591

B.1.2 V based membranes

Table S3 $V_{97.5}Fe_{2.5}$ solubility at different temperatures.

Reference	Alloy composition (at%)	Thickness (mm)	Preparation method	T (K)	Hydrogen content (H/M)	Solubility (MPa)	$P^{0.5}$ ($MPa^{0.5}$)	Solubility ($mol\cdot m^{-3}\cdot MPa^{-0.5}$)
[2]	$V_{97.5}Fe_{2.5}$	-	Metallurgical	523	0.089	0.007	0.026	300614.338
[2]	$V_{97.5}Fe_{2.5}$	-	Metallurgical	523	0.136	0.010	0.038	381346.388
[2]	$V_{97.5}Fe_{2.5}$	-	Metallurgical	523	0.175	0.015	0.051	532788.686
[2]	$V_{97.5}Fe_{2.5}$	-	Metallurgical	523	0.217	0.017	0.061	612252.579
[2]	$V_{97.5}Fe_{2.5}$	-	Metallurgical	523	0.273	0.017	0.069	614400.484
[2]	$V_{97.5}Fe_{2.5}$	-	Metallurgical	523	0.345	0.025	0.092	132915.681
[2]	$V_{97.5}Fe_{2.5}$	-	Metallurgical	523	0.452	0.053	0.154	79470.971
[2]	$V_{97.5}Fe_{2.5}$	-	Metallurgical	523	0.527	0.129	0.261	46932.255
[2]	$V_{97.5}Fe_{2.5}$	-	Metallurgical	523	0.560	0.227	0.357	34326.159
[2]	$V_{97.5}Fe_{2.5}$	-	Metallurgical	523	0.578	0.329	0.436	28095.753
[2]	$V_{97.5}Fe_{2.5}$	-	Metallurgical	523	0.595	0.421	0.501	24462.634
[2]	$V_{97.5}Fe_{2.5}$	-	Metallurgical	523	0.613	0.610	0.612	20022.517
[2]	$V_{97.5}Fe_{2.5}$	-	Metallurgical	523	0.628	0.866	0.738	16592.184

[2]	$V_{97.5}Fe_{2.5}$	-	Metallurgical	523	0.649	1.286	0.914	13397.158
[2]	$V_{97.5}Fe_{2.5}$	-	Metallurgical	523	0.669	1.865	1.117	10965.492
[2]	$V_{97.5}Fe_{2.5}$	-	Metallurgical	523	0.683	2.595	1.331	9199.886
[2]	$V_{97.5}Fe_{2.5}$	-	Metallurgical	523	0.704	4.056	1.690	7246.907
[2]	$V_{97.5}Fe_{2.5}$	-	Metallurgical	573	0.057	0.027	0.039	218027.224
[2]	$V_{97.5}Fe_{2.5}$	-	Metallurgical	573	0.081	0.037	0.055	247479.651
[2]	$V_{97.5}Fe_{2.5}$	-	Metallurgical	573	0.106	0.042	0.067	264262.774
[2]	$V_{97.5}Fe_{2.5}$	-	Metallurgical	573	0.142	0.047	0.082	277739.014
[2]	$V_{97.5}Fe_{2.5}$	-	Metallurgical	573	0.182	0.055	0.100	283199.625
[2]	$V_{97.5}Fe_{2.5}$	-	Metallurgical	573	0.248	0.063	0.125	273872.915
[2]	$V_{97.5}Fe_{2.5}$	-	Metallurgical	573	0.340	0.088	0.173	211395.054
[2]	$V_{97.5}Fe_{2.5}$	-	Metallurgical	573	0.426	0.162	0.263	57098.851
[2]	$V_{97.5}Fe_{2.5}$	-	Metallurgical	573	0.478	0.262	0.354	42386.197
[2]	$V_{97.5}Fe_{2.5}$	-	Metallurgical	573	0.517	0.408	0.459	32653.503
[2]	$V_{97.5}Fe_{2.5}$	-	Metallurgical	573	0.548	0.649	0.597	25124.542
[2]	$V_{97.5}Fe_{2.5}$	-	Metallurgical	573	0.572	0.924	0.727	20615.198
[2]	$V_{97.5}Fe_{2.5}$	-	Metallurgical	573	0.601	1.432	0.928	16158.834
[2]	$V_{97.5}Fe_{2.5}$	-	Metallurgical	573	0.616	2.079	1.132	13242.270

[2]	$V_{97.5}Fe_{2.5}$	-	Metallurgical	573	0.641	2.761	1.331	11262.196
[2]	$V_{97.5}Fe_{2.5}$	-	Metallurgical	573	0.663	4.275	1.683	8904.390
[2]	$V_{97.5}Fe_{2.5}$	-	Metallurgical	623	0.032	0.017	0.023	162768.935
[2]	$V_{97.5}Fe_{2.5}$	-	Metallurgical	623	0.041	0.047	0.044	156673.111
[2]	$V_{97.5}Fe_{2.5}$	-	Metallurgical	623	0.051	0.064	0.057	152915.321
[2]	$V_{97.5}Fe_{2.5}$	-	Metallurgical	623	0.207	0.171	0.188	118417.211
[2]	$V_{97.5}Fe_{2.5}$	-	Metallurgical	623	0.222	0.184	0.202	115030.284
[2]	$V_{97.5}Fe_{2.5}$	-	Metallurgical	623	0.245	0.189	0.215	111896.587
[2]	$V_{97.5}Fe_{2.5}$	-	Metallurgical	623	0.265	0.202	0.231	108085.499
[2]	$V_{97.5}Fe_{2.5}$	-	Metallurgical	623	0.286	0.224	0.253	103088.998
[2]	$V_{97.5}Fe_{2.5}$	-	Metallurgical	623	0.310	0.239	0.272	98895.049
[2]	$V_{97.5}Fe_{2.5}$	-	Metallurgical	623	0.335	0.274	0.303	92232.145
[2]	$V_{97.5}Fe_{2.5}$	-	Metallurgical	623	0.358	0.313	0.335	85635.241
[2]	$V_{97.5}Fe_{2.5}$	-	Metallurgical	623	0.382	0.358	0.370	78765.777
[2]	$V_{97.5}Fe_{2.5}$	-	Metallurgical	623	0.410	0.422	0.416	70195.904
[2]	$V_{97.5}Fe_{2.5}$	-	Metallurgical	623	0.432	0.515	0.472	60589.989
[2]	$V_{97.5}Fe_{2.5}$	-	Metallurgical	623	0.452	0.611	0.526	52086.293
[2]	$V_{97.5}Fe_{2.5}$	-	Metallurgical	623	0.486	0.816	0.630	37976.829

[2]	$V_{97.5}Fe_{2.5}$	-	Metallurgical	623	0.514	1.067	0.741	26076.858
[2]	$V_{97.5}Fe_{2.5}$	-	Metallurgical	623	0.539	1.406	0.871	16080.648
[2]	$V_{97.5}Fe_{2.5}$	-	Metallurgical	623	0.561	1.838	1.015	9878.670
[2]	$V_{97.5}Fe_{2.5}$	-	Metallurgical	623	0.583	2.491	1.206	9206.447
[2]	$V_{97.5}Fe_{2.5}$	-	Metallurgical	623	0.617	3.831	1.538	27234.180*
[2]	$V_{97.5}Fe_{2.5}$	-	Metallurgical	673	0.126	0.347	0.209	97106.392
[2]	$V_{97.5}Fe_{2.5}$	-	Metallurgical	673	0.142	0.371	0.229	97710.694
[2]	$V_{97.5}Fe_{2.5}$	-	Metallurgical	673	0.159	0.388	0.249	97619.020
[2]	$V_{97.5}Fe_{2.5}$	-	Metallurgical	673	0.179	0.429	0.277	96415.800
[2]	$V_{97.5}Fe_{2.5}$	-	Metallurgical	673	0.201	0.467	0.306	94123.463
[2]	$V_{97.5}Fe_{2.5}$	-	Metallurgical	673	0.228	0.509	0.341	90221.914
[2]	$V_{97.5}Fe_{2.5}$	-	Metallurgical	673	0.255	0.546	0.373	85774.630
[2]	$V_{97.5}Fe_{2.5}$	-	Metallurgical	673	0.285	0.628	0.423	77812.423
[2]	$V_{97.5}Fe_{2.5}$	-	Metallurgical	673	0.313	0.686	0.463	70994.360
[2]	$V_{97.5}Fe_{2.5}$	-	Metallurgical	673	0.343	0.777	0.516	61834.753
[2]	$V_{97.5}Fe_{2.5}$	-	Metallurgical	673	0.387	0.971	0.613	46529.209
[2]	$V_{97.5}Fe_{2.5}$	-	Metallurgical	673	0.422	1.277	0.734	32145.271
[2]	$V_{97.5}Fe_{2.5}$	-	Metallurgical	673	0.454	1.705	0.880	22779.798

[2]	$V_{97.5}Fe_{2.5}$	-	Metallurgical	673	0.483	2.135	1.015	19448.909
[2]	$V_{97.5}Fe_{2.5}$	-	Metallurgical	673	0.503	2.737	1.173	17054.956
[2]	$V_{97.5}Fe_{2.5}$	-	Metallurgical	673	0.536	4.416	1.538	3543.944*
[2]	$V_{97.5}Fe_{2.5}$	-	Metallurgical	723	0.110	0.724	0.282	59149.366
[2]	$V_{97.5}Fe_{2.5}$	-	Metallurgical	723	0.125	0.775	0.311	60032.173
[2]	$V_{97.5}Fe_{2.5}$	-	Metallurgical	723	0.145	0.829	0.347	60665.215
[2]	$V_{97.5}Fe_{2.5}$	-	Metallurgical	723	0.167	0.899	0.387	60833.339
[2]	$V_{97.5}Fe_{2.5}$	-	Metallurgical	723	0.189	0.998	0.435	60352.674
[2]	$V_{97.5}Fe_{2.5}$	-	Metallurgical	723	0.216	1.070	0.481	59261.822
[2]	$V_{97.5}Fe_{2.5}$	-	Metallurgical	723	0.261	1.314	0.586	54806.495
[2]	$V_{97.5}Fe_{2.5}$	-	Metallurgical	723	0.308	1.572	0.695	47991.976
[2]	$V_{97.5}Fe_{2.5}$	-	Metallurgical	723	0.352	1.836	0.804	40036.359
[2]	$V_{97.5}Fe_{2.5}$	-	Metallurgical	723	0.388	2.340	0.953	28736.060
[2]	$V_{97.5}Fe_{2.5}$	-	Metallurgical	723	0.422	3.034	1.132	17848.596
[2]	$V_{97.5}Fe_{2.5}$	-	Metallurgical	723	0.450	3.727	1.295	13713.380
[2]	$V_{97.5}Fe_{2.5}$	-	Metallurgical	723	0.474	4.810	1.510	22172.534*
[2]	$V_{97.5}Fe_{2.5}$	-	Metallurgical	773	0.129	1.388	0.423	41512.559
[2]	$V_{97.5}Fe_{2.5}$	-	Metallurgical	773	0.148	1.506	0.472	41769.300

[2]	$V_{97.5}Fe_{2.5}$	-	Metallurgical	773	0.168	1.615	0.521	41859.198
[2]	$V_{97.5}Fe_{2.5}$	-	Metallurgical	773	0.206	1.891	0.624	41495.266
[2]	$V_{97.5}Fe_{2.5}$	-	Metallurgical	773	0.245	2.242	0.741	40174.194
[2]	$V_{97.5}Fe_{2.5}$	-	Metallurgical	773	0.287	2.638	0.871	37567.216
[2]	$V_{97.5}Fe_{2.5}$	-	Metallurgical	773	0.328	3.143	1.015	33280.565
[2]	$V_{97.5}Fe_{2.5}$	-	Metallurgical	773	0.367	3.817	1.183	26428.634
[2]	$V_{97.5}Fe_{2.5}$	-	Metallurgical	773	0.401	4.842	1.393	15084.408
[2]	$V_{97.5}Fe_{2.5}$	-	Metallurgical	773	0.421	5.414	1.510	7437.284

* data not fully reliable

Table S4 $V_{92.5}Fe_{7.5}$ solubility at different temperatures.

Reference	Alloy composition (at%)	Thickness (mm)	Preparation method	T (K)	Hydrogen content (H/M)	Solubility (MPa)	$P^{0.5}$ ($MPa^{0.5}$)	Solubility ($mol\cdot m^{-3}\cdot MPa^{-0.5}$)
[2]	$V_{92.5}Fe_{7.5}$	0.508	Metallurgical	473	0.039	0.008	0.017	346845.749
[2]	$V_{92.5}Fe_{7.5}$	0.508	Metallurgical	473	0.078	0.011	0.030	324861.343
[2]	$V_{92.5}Fe_{7.5}$	0.508	Metallurgical	473	0.116	0.020	0.048	296182.315
[2]	$V_{92.5}Fe_{7.5}$	0.508	Metallurgical	473	0.151	0.022	0.058	280319.771
[2]	$V_{92.5}Fe_{7.5}$	0.508	Metallurgical	473	0.198	0.029	0.075	255625.859
[2]	$V_{92.5}Fe_{7.5}$	0.508	Metallurgical	473	0.256	0.040	0.101	222430.394
[2]	$V_{92.5}Fe_{7.5}$	0.508	Metallurgical	473	0.326	0.065	0.146	171582.521
[2]	$V_{92.5}Fe_{7.5}$	0.508	Metallurgical	473	0.399	0.115	0.214	113517.449
[2]	$V_{92.5}Fe_{7.5}$	0.508	Metallurgical	473	0.455	0.192	0.295	67403.675
[2]	$V_{92.5}Fe_{7.5}$	0.508	Metallurgical	473	0.487	0.282	0.371	41566.256
[2]	$V_{92.5}Fe_{7.5}$	0.508	Metallurgical	473	0.510	0.416	0.461	25438.605
[2]	$V_{92.5}Fe_{7.5}$	0.508	Metallurgical	473	0.538	0.635	0.585	18585.588
[2]	$V_{92.5}Fe_{7.5}$	0.508	Metallurgical	473	0.558	0.928	0.720	18753.722
[2]	$V_{92.5}Fe_{7.5}$	0.508	Metallurgical	473	0.584	1.427	0.913	18193.531
[2]	$V_{92.5}Fe_{7.5}$	0.508	Metallurgical	473	0.616	2.003	1.111	21000.841

[2]	$V_{92.5}Fe_{7.5}$	0.508	Metallurgical	523	0.040	0.023	0.030	177191.078
[2]	$V_{92.5}Fe_{7.5}$	0.508	Metallurgical	523	0.053	0.037	0.044	171278.653
[2]	$V_{92.5}Fe_{7.5}$	0.508	Metallurgical	523	0.065	0.050	0.057	165852.036
[2]	$V_{92.5}Fe_{7.5}$	0.508	Metallurgical	523	0.091	0.067	0.078	157379.251
[2]	$V_{92.5}Fe_{7.5}$	0.508	Metallurgical	523	0.132	0.081	0.103	147550.846
[2]	$V_{92.5}Fe_{7.5}$	0.508	Metallurgical	523	0.159	0.099	0.126	139291.877
[2]	$V_{92.5}Fe_{7.5}$	0.508	Metallurgical	523	0.200	0.117	0.153	129631.378
[2]	$V_{92.5}Fe_{7.5}$	0.508	Metallurgical	523	0.230	0.137	0.177	121210.726
[2]	$V_{92.5}Fe_{7.5}$	0.508	Metallurgical	523	0.246	0.148	0.191	116633.679
[2]	$V_{92.5}Fe_{7.5}$	0.508	Metallurgical	523	0.266	0.167	0.211	110442.820
[2]	$V_{92.5}Fe_{7.5}$	0.508	Metallurgical	523	0.286	0.185	0.230	104628.610
[2]	$V_{92.5}Fe_{7.5}$	0.508	Metallurgical	523	0.304	0.215	0.256	96999.547
[2]	$V_{92.5}Fe_{7.5}$	0.508	Metallurgical	523	0.326	0.249	0.285	88893.528
[2]	$V_{92.5}Fe_{7.5}$	0.508	Metallurgical	523	0.349	0.295	0.321	79706.716
[2]	$V_{92.5}Fe_{7.5}$	0.508	Metallurgical	523	0.367	0.348	0.357	71102.593
[2]	$V_{92.5}Fe_{7.5}$	0.508	Metallurgical	523	0.391	0.423	0.407	60575.851
[2]	$V_{92.5}Fe_{7.5}$	0.508	Metallurgical	523	0.410	0.513	0.458	51126.032
[2]	$V_{92.5}Fe_{7.5}$	0.508	Metallurgical	523	0.432	0.615	0.516	42102.193

[2]	$V_{92.5}Fe_{7.5}$	0.508	Metallurgical	523	0.464	0.845	0.626	29293.852
[2]	$V_{92.5}Fe_{7.5}$	0.508	Metallurgical	523	0.491	1.127	0.744	21636.677
[2]	$V_{92.5}Fe_{7.5}$	0.508	Metallurgical	523	0.521	1.500	0.884	19745.898
[2]	$V_{92.5}Fe_{7.5}$	0.508	Metallurgical	523	0.551	1.960	1.040	25524.338*
[2]	$V_{92.5}Fe_{7.5}$	0.508	Metallurgical	523	0.588	2.489	1.209	39572.129*
[2]	$V_{92.5}Fe_{7.5}$	0.508	Metallurgical	573	0.015	0.041	0.025	68906.568*
[2]	$V_{92.5}Fe_{7.5}$	0.508	Metallurgical	573	0.027	0.100	0.052	81069.690*
[2]	$V_{92.5}Fe_{7.5}$	0.508	Metallurgical	573	0.133	0.263	0.187	100559.174
[2]	$V_{92.5}Fe_{7.5}$	0.508	Metallurgical	573	0.168	0.301	0.225	97986.894
[2]	$V_{92.5}Fe_{7.5}$	0.508	Metallurgical	573	0.189	0.325	0.248	95408.934
[2]	$V_{92.5}Fe_{7.5}$	0.508	Metallurgical	573	0.209	0.373	0.279	90997.306
[2]	$V_{92.5}Fe_{7.5}$	0.508	Metallurgical	573	0.233	0.415	0.311	85670.073
[2]	$V_{92.5}Fe_{7.5}$	0.508	Metallurgical	573	0.254	0.472	0.346	79249.590
[2]	$V_{92.5}Fe_{7.5}$	0.508	Metallurgical	573	0.283	0.537	0.390	71059.226
[2]	$V_{92.5}Fe_{7.5}$	0.508	Metallurgical	573	0.308	0.611	0.434	62889.626
[2]	$V_{92.5}Fe_{7.5}$	0.508	Metallurgical	573	0.332	0.704	0.484	54404.521
[2]	$V_{92.5}Fe_{7.5}$	0.508	Metallurgical	573	0.372	0.948	0.594	40028.304
[2]	$V_{92.5}Fe_{7.5}$	0.508	Metallurgical	573	0.410	1.241	0.713	31941.315

[2]	$V_{92.5}Fe_{7.5}$	0.508	Metallurgical	573	0.443	1.588	0.838	29774.585
[2]	$V_{92.5}Fe_{7.5}$	0.508	Metallurgical	573	0.476	2.085	0.996	29563.820
[2]	$V_{92.5}Fe_{7.5}$	0.508	Metallurgical	573	0.515	2.547	1.145	26057.941
[2]	$V_{92.5}Fe_{7.5}$	0.508	Metallurgical	573	0.548	3.238	1.332	20810.022
[2]	$V_{92.5}Fe_{7.5}$	0.508	Metallurgical	573	0.577	3.735	1.468	34729.852*
[2]	$V_{92.5}Fe_{7.5}$	0.508	Metallurgical	623	0.080	0.454	0.191	57958.404
[2]	$V_{92.5}Fe_{7.5}$	0.508	Metallurgical	623	0.104	0.568	0.243	59821.698
[2]	$V_{92.5}Fe_{7.5}$	0.508	Metallurgical	623	0.116	0.648	0.274	60318.904
[2]	$V_{92.5}Fe_{7.5}$	0.508	Metallurgical	623	0.132	0.691	0.302	60443.803
[2]	$V_{92.5}Fe_{7.5}$	0.508	Metallurgical	623	0.155	0.772	0.345	60063.953
[2]	$V_{92.5}Fe_{7.5}$	0.508	Metallurgical	623	0.175	0.863	0.388	59100.393
[2]	$V_{92.5}Fe_{7.5}$	0.508	Metallurgical	623	0.201	0.947	0.436	57437.161
[2]	$V_{92.5}Fe_{7.5}$	0.508	Metallurgical	623	0.226	1.102	0.499	54541.432
[2]	$V_{92.5}Fe_{7.5}$	0.508	Metallurgical	623	0.269	1.304	0.592	49290.027
[2]	$V_{92.5}Fe_{7.5}$	0.508	Metallurgical	623	0.309	1.597	0.703	42368.039
[2]	$V_{92.5}Fe_{7.5}$	0.508	Metallurgical	623	0.347	1.968	0.826	34972.314
[2]	$V_{92.5}Fe_{7.5}$	0.508	Metallurgical	623	0.386	2.441	0.971	28116.779
[2]	$V_{92.5}Fe_{7.5}$	0.508	Metallurgical	623	0.423	3.082	1.141	23745.130

[2]	$V_{92.5}Fe_{7.5}$	0.508	Metallurgical	623	0.458	3.861	1.329	24037.869
[2]	$V_{92.5}Fe_{7.5}$	0.508	Metallurgical	623	0.490	4.479	1.482	27365.722
[2]	$V_{92.5}Fe_{7.5}$	0.508	Metallurgical	673	0.073	0.990	0.268	38139.726
[2]	$V_{92.5}Fe_{7.5}$	0.508	Metallurgical	673	0.078	1.213	0.307	39192.801
[2]	$V_{92.5}Fe_{7.5}$	0.508	Metallurgical	673	0.094	1.267	0.345	39975.045
[2]	$V_{92.5}Fe_{7.5}$	0.508	Metallurgical	673	0.111	1.413	0.395	40640.799
[2]	$V_{92.5}Fe_{7.5}$	0.508	Metallurgical	673	0.128	1.487	0.436	40908.458
[2]	$V_{92.5}Fe_{7.5}$	0.508	Metallurgical	673	0.148	1.710	0.504	40846.240
[2]	$V_{92.5}Fe_{7.5}$	0.508	Metallurgical	673	0.181	1.975	0.598	39857.218
[2]	$V_{92.5}Fe_{7.5}$	0.508	Metallurgical	673	0.216	2.413	0.722	37281.030
[2]	$V_{92.5}Fe_{7.5}$	0.508	Metallurgical	673	0.255	2.819	0.848	33687.451
[2]	$V_{92.5}Fe_{7.5}$	0.508	Metallurgical	673	0.293	3.340	0.989	29148.825
[2]	$V_{92.5}Fe_{7.5}$	0.508	Metallurgical	673	0.329	4.030	1.152	24165.999
[2]	$V_{92.5}Fe_{7.5}$	0.508	Metallurgical	673	0.363	5.051	1.355	20042.294
[2]	$V_{92.5}Fe_{7.5}$	0.508	Metallurgical	673	0.390	5.838	1.509	19713.582
[2]	$V_{92.5}Fe_{7.5}$	0.508	Metallurgical	723	0.108	2.395	0.508	29527.487
[2]	$V_{92.5}Fe_{7.5}$	0.508	Metallurgical	723	0.134	2.755	0.608	30047.890
[2]	$V_{92.5}Fe_{7.5}$	0.508	Metallurgical	723	0.161	3.182	0.715	30187.153

[2]	$V_{92.5}Fe_{7.5}$	0.508	Metallurgical	723	0.195	3.693	0.848	29760.652
[2]	$V_{92.5}Fe_{7.5}$	0.508	Metallurgical	723	0.227	4.302	0.989	28581.915
[2]	$V_{92.5}Fe_{7.5}$	0.508	Metallurgical	723	0.267	5.064	1.162	26120.471
[2]	$V_{92.5}Fe_{7.5}$	0.508	Metallurgical	723	0.303	6.055	1.355	22039.135
[2]	$V_{92.5}Fe_{7.5}$	0.508	Metallurgical	723	0.329	6.780	1.494	18210.700
[2]	$V_{92.5}Fe_{7.5}$	0.508	Metallurgical	773	0.111	3.663	0.636	21797.731
[2]	$V_{92.5}Fe_{7.5}$	0.508	Metallurgical	773	0.133	4.360	0.762	22067.017
[2]	$V_{92.5}Fe_{7.5}$	0.508	Metallurgical	773	0.160	4.933	0.887	22351.395
[2]	$V_{92.5}Fe_{7.5}$	0.508	Metallurgical	773	0.185	5.891	1.043	22725.310
[2]	$V_{92.5}Fe_{7.5}$	0.508	Metallurgical	773	0.220	6.840	1.227	23193.935
[2]	$V_{92.5}Fe_{7.5}$	0.508	Metallurgical	773	0.273	8.187	1.494	23934.203

* data not fully reliable

Table S5 $V_{90}Fe_{10}$ solubility at different temperatures.

Reference	Alloy composition (at%)	Thickness (mm)	Preparation method	T (K)	Hydrogen content (H/M)	Solubility (MPa)	$P^{0.5}$ ($MPa^{0.5}$)	Solubility ($mol\cdot m^{-3}\cdot MPa^{-0.5}$)
[2]	$V_{90}Fe_{10}$	0.437	Metallurgical	473	0.020	0.008	0.013	182023.569
[2]	$V_{90}Fe_{10}$	0.437	Metallurgical	473	0.039	0.016	0.025	176453.168
[2]	$V_{90}Fe_{10}$	0.437	Metallurgical	473	0.051	0.035	0.042	169003.389
[2]	$V_{90}Fe_{10}$	0.437	Metallurgical	473	0.257	0.178	0.214	107122.902
[2]	$V_{90}Fe_{10}$	0.437	Metallurgical	473	0.278	0.195	0.233	101679.357
[2]	$V_{90}Fe_{10}$	0.437	Metallurgical	473	0.292	0.233	0.261	93876.821
[2]	$V_{90}Fe_{10}$	0.437	Metallurgical	473	0.314	0.262	0.287	87200.549
[2]	$V_{90}Fe_{10}$	0.437	Metallurgical	473	0.332	0.327	0.329	77256.472
[2]	$V_{90}Fe_{10}$	0.437	Metallurgical	473	0.358	0.365	0.362	70323.239
[2]	$V_{90}Fe_{10}$	0.437	Metallurgical	473	0.381	0.424	0.402	62478.781
[2]	$V_{90}Fe_{10}$	0.437	Metallurgical	473	0.409	0.531	0.466	51711.740
[2]	$V_{90}Fe_{10}$	0.437	Metallurgical	473	0.433	0.593	0.507	45827.242
[2]	$V_{90}Fe_{10}$	0.437	Metallurgical	473	0.466	0.823	0.619	33172.318
[2]	$V_{90}Fe_{10}$	0.437	Metallurgical	473	0.492	1.067	0.724	25109.885
[2]	$V_{90}Fe_{10}$	0.437	Metallurgical	473	0.512	1.531	0.885	18158.820

[2]	$V_{90}Fe_{10}$	0.437	Metallurgical	473	0.538	1.916	1.016	15736.253
[2]	$V_{90}Fe_{10}$	0.437	Metallurgical	473	0.557	2.536	1.189	14843.428
[2]	$V_{90}Fe_{10}$	0.437	Metallurgical	473	0.579	3.272	1.376	14636.418
[2]	$V_{90}Fe_{10}$	0.437	Metallurgical	473	0.598	3.916	1.530	13716.880
[2]	$V_{90}Fe_{10}$	0.437	Metallurgical	523	0.017	0.078	0.037	98166.547
[2]	$V_{90}Fe_{10}$	0.437	Metallurgical	523	0.032	0.075	0.049	96506.810
[2]	$V_{90}Fe_{10}$	0.437	Metallurgical	523	0.041	0.084	0.059	95204.861
[2]	$V_{90}Fe_{10}$	0.437	Metallurgical	523	0.170	0.346	0.243	72234.798
[2]	$V_{90}Fe_{10}$	0.437	Metallurgical	523	0.189	0.376	0.267	69501.374
[2]	$V_{90}Fe_{10}$	0.437	Metallurgical	523	0.208	0.431	0.299	65917.532
[2]	$V_{90}Fe_{10}$	0.437	Metallurgical	523	0.230	0.523	0.347	60943.751
[2]	$V_{90}Fe_{10}$	0.437	Metallurgical	523	0.252	0.566	0.377	57881.957
[2]	$V_{90}Fe_{10}$	0.437	Metallurgical	523	0.272	0.688	0.433	52592.947
[2]	$V_{90}Fe_{10}$	0.437	Metallurgical	523	0.295	0.783	0.481	48286.268
[2]	$V_{90}Fe_{10}$	0.437	Metallurgical	523	0.332	1.062	0.593	39354.526
[2]	$V_{90}Fe_{10}$	0.437	Metallurgical	523	0.361	1.367	0.702	32241.156
[2]	$V_{90}Fe_{10}$	0.437	Metallurgical	523	0.390	1.733	0.822	26183.151
[2]	$V_{90}Fe_{10}$	0.437	Metallurgical	523	0.418	2.365	0.994	20848.232

[2]	$V_{90}Fe_{10}$	0.437	Metallurgical	523	0.449	2.953	1.152	19570.263
[2]	$V_{90}Fe_{10}$	0.437	Metallurgical	523	0.487	3.574	1.320	22166.820
[2]	$V_{90}Fe_{10}$	0.437	Metallurgical	523	0.512	4.388	1.499	29615.843
[2]	$V_{90}Fe_{10}$	0.437	Metallurgical	573	0.089	0.572	0.225	49679.955
[2]	$V_{90}Fe_{10}$	0.437	Metallurgical	573	0.145	0.845	0.350	45857.720
[2]	$V_{90}Fe_{10}$	0.437	Metallurgical	573	0.164	0.962	0.398	44427.753
[2]	$V_{90}Fe_{10}$	0.437	Metallurgical	573	0.185	1.102	0.451	42810.991
[2]	$V_{90}Fe_{10}$	0.437	Metallurgical	573	0.207	1.244	0.507	41131.087
[2]	$V_{90}Fe_{10}$	0.437	Metallurgical	573	0.240	1.529	0.606	38174.852
[2]	$V_{90}Fe_{10}$	0.437	Metallurgical	573	0.273	1.961	0.732	34410.482
[2]	$V_{90}Fe_{10}$	0.437	Metallurgical	573	0.307	2.298	0.840	31240.410
[2]	$V_{90}Fe_{10}$	0.437	Metallurgical	573	0.343	3.063	1.025	25829.762
[2]	$V_{90}Fe_{10}$	0.437	Metallurgical	573	0.374	3.862	1.202	20752.528
[2]	$V_{90}Fe_{10}$	0.437	Metallurgical	573	0.404	4.596	1.363	16153.847
[2]	$V_{90}Fe_{10}$	0.437	Metallurgical	573	0.426	5.613	1.547	11001.976
[2]	$V_{90}Fe_{10}$	0.437	Metallurgical	623	0.093	1.349	0.354	33519.299
[2]	$V_{90}Fe_{10}$	0.437	Metallurgical	623	0.108	1.410	0.390	33171.027
[2]	$V_{90}Fe_{10}$	0.437	Metallurgical	623	0.128	1.591	0.451	32563.815

[2]	$V_{90}Fe_{10}$	0.437	Metallurgical	623	0.140	1.841	0.507	32013.298
[2]	$V_{90}Fe_{10}$	0.437	Metallurgical	623	0.173	2.078	0.600	31096.540
[2]	$V_{90}Fe_{10}$	0.437	Metallurgical	623	0.204	2.635	0.732	29789.427
[2]	$V_{90}Fe_{10}$	0.437	Metallurgical	623	0.237	3.099	0.857	28557.100
[2]	$V_{90}Fe_{10}$	0.437	Metallurgical	623	0.269	3.834	1.016	26994.885
[2]	$V_{90}Fe_{10}$	0.437	Metallurgical	623	0.303	4.670	1.189	25285.979
[2]	$V_{90}Fe_{10}$	0.437	Metallurgical	623	0.337	5.508	1.363	23563.351
[2]	$V_{90}Fe_{10}$	0.437	Metallurgical	623	0.367	6.250	1.514	22080.396
[2]	$V_{90}Fe_{10}$	0.437	Metallurgical	673	0.093	2.877	0.517	25064.532
[2]	$V_{90}Fe_{10}$	0.437	Metallurgical	673	0.115	3.195	0.606	25472.745
[2]	$V_{90}Fe_{10}$	0.437	Metallurgical	673	0.138	3.969	0.740	25651.059
[2]	$V_{90}Fe_{10}$	0.437	Metallurgical	673	0.167	4.392	0.857	25370.159
[2]	$V_{90}Fe_{10}$	0.437	Metallurgical	673	0.199	5.177	1.016	24344.219
[2]	$V_{90}Fe_{10}$	0.437	Metallurgical	673	0.231	6.109	1.189	22371.383
[2]	$V_{90}Fe_{10}$	0.437	Metallurgical	673	0.263	7.356	1.391	18933.134
[2]	$V_{90}Fe_{10}$	0.437	Metallurgical	673	0.285	8.035	1.514	16273.668
[2]	$V_{90}Fe_{10}$	0.437	Metallurgical	723	0.128	6.129	0.885	19046.225
[2]	$V_{90}Fe_{10}$	0.437	Metallurgical	723	0.150	7.023	1.025	18771.982

[2]	$V_{90}Fe_{10}$	0.437	Metallurgical	723	0.174	8.099	1.189	18095.887
[2]	$V_{90}Fe_{10}$	0.437	Metallurgical	723	0.224	10.681	1.547	15274.371
[2]	$V_{90}Fe_{10}$	0.437	Metallurgical	773	0.108	7.126	0.876	12040.684
[2]	$V_{90}Fe_{10}$	0.437	Metallurgical	773	0.122	8.797	1.036	11719.427
[2]	$V_{90}Fe_{10}$	0.437	Metallurgical	773	0.138	10.234	1.189	11706.675
[2]	$V_{90}Fe_{10}$	0.437	Metallurgical	773	0.173	13.817	1.547	12796.328

Table S6 $V_{85}Ni_{15}$ solubility at different temperatures.

Reference	Alloy composition (at%)	Thickness (mm)	Preparation method	T (K)	Hydrogen content (H/M)	Solubility (MPa)	$P^{0.5}$ ($MPa^{0.5}$)	Solubility ($mol\cdot m^{-3}\cdot MPa^{-0.5}$)
[3]	$V_{85}Ni_{15}$	1	Metallurgical	573	0.207	0.747	0.393	108823.662
[3]	$V_{85}Ni_{15}$	1	Metallurgical	573	0.249	0.732	0.427	102303.117
[3]	$V_{85}Ni_{15}$	1	Metallurgical	573	0.288	0.856	0.496	86265.291
[3]	$V_{85}Ni_{15}$	1	Metallurgical	573	0.302	0.833	0.501	84966.428
[3]	$V_{85}Ni_{15}$	1	Metallurgical	573	0.366	1.062	0.623	57139.404
[3]	$V_{85}Ni_{15}$	1	Metallurgical	573	0.382	1.121	0.654	51324.423
[3]	$V_{85}Ni_{15}$	1	Metallurgical	573	0.415	1.355	0.750	38038.602
[3]	$V_{85}Ni_{15}$	1	Metallurgical	573	0.425	1.506	0.800	33731.264
[3]	$V_{85}Ni_{15}$	1	Metallurgical	573	0.436	1.556	0.823	32207.446
[3]	$V_{85}Ni_{15}$	1	Metallurgical	573	0.450	1.719	0.880	29299.458
[3]	$V_{85}Ni_{15}$	1	Metallurgical	573	0.463	1.830	0.920	27301.256
[3]	$V_{85}Ni_{15}$	1	Metallurgical	573	0.475	2.132	1.007	19955.365*
[3]	$V_{85}Ni_{15}$	1	Metallurgical	573	0.478	2.371	1.065	13853.028*
[3]	$V_{85}Ni_{15}$	1	Metallurgical	573	0.486	2.547	1.113	27207.429*
[3]	$V_{85}Ni_{15}$	1	Metallurgical	598	0.173	1.050	0.427	100117.891

[3]	$V_{85}Ni_{15}$	1	Metallurgical	598	0.195	1.027	0.448	98876.009
[3]	$V_{85}Ni_{15}$	1	Metallurgical	598	0.225	0.997	0.474	96558.820
[3]	$V_{85}Ni_{15}$	1	Metallurgical	598	0.256	1.003	0.507	92688.766
[3]	$V_{85}Ni_{15}$	1	Metallurgical	598	0.300	1.124	0.581	80651.186
[3]	$V_{85}Ni_{15}$	1	Metallurgical	598	0.344	1.294	0.667	63435.094
[3]	$V_{85}Ni_{15}$	1	Metallurgical	598	0.381	1.499	0.755	45509.701
[3]	$V_{85}Ni_{15}$	1	Metallurgical	598	0.407	1.684	0.828	33142.256
[3]	$V_{85}Ni_{15}$	1	Metallurgical	598	0.428	1.948	0.913	24309.686
[3]	$V_{85}Ni_{15}$	1	Metallurgical	598	0.443	2.158	0.978	23568.826
[3]	$V_{85}Ni_{15}$	1	Metallurgical	598	0.451	2.238	1.005	25181.408*
[3]	$V_{85}Ni_{15}$	1	Metallurgical	598	0.456	2.443	1.056	31754.316*
[3]	$V_{85}Ni_{15}$	1	Metallurgical	598	0.465	2.569	1.093	39953.757*
[3]	$V_{85}Ni_{15}$	1	Metallurgical	623	0.235	1.343	0.562	92390.128
[3]	$V_{85}Ni_{15}$	1	Metallurgical	623	0.266	1.251	0.576	88689.040
[3]	$V_{85}Ni_{15}$	1	Metallurgical	623	0.312	1.407	0.662	66589.592
[3]	$V_{85}Ni_{15}$	1	Metallurgical	623	0.346	1.652	0.755	47239.097
[3]	$V_{85}Ni_{15}$	1	Metallurgical	623	0.368	1.835	0.822	37932.601
[3]	$V_{85}Ni_{15}$	1	Metallurgical	623	0.381	1.809	0.830	37048.982

[3]	$V_{85}Ni_{15}$	1	Metallurgical	623	0.401	1.984	0.892	32352.637
[3]	$V_{85}Ni_{15}$	1	Metallurgical	623	0.403	2.150	0.930	30925.355
[3]	$V_{85}Ni_{15}$	1	Metallurgical	623	0.416	2.325	0.984	30284.557
[3]	$V_{85}Ni_{15}$	1	Metallurgical	623	0.426	2.449	1.021	30304.201
[3]	$V_{85}Ni_{15}$	1	Metallurgical	623	0.431	2.585	1.056	30255.651
[3]	$V_{85}Ni_{15}$	1	Metallurgical	623	0.447	2.705	1.099	29476.233
[3]	$V_{85}Ni_{15}$	1	Metallurgical	648	0.217	1.703	0.608	68075.365
[3]	$V_{85}Ni_{15}$	1	Metallurgical	648	0.259	1.666	0.657	66424.597
[3]	$V_{85}Ni_{15}$	1	Metallurgical	648	0.305	1.852	0.752	59983.012
[3]	$V_{85}Ni_{15}$	1	Metallurgical	648	0.334	2.035	0.825	52162.492
[3]	$V_{85}Ni_{15}$	1	Metallurgical	648	0.357	2.169	0.880	44617.460
[3]	$V_{85}Ni_{15}$	1	Metallurgical	648	0.369	2.326	0.926	37213.843
[3]	$V_{85}Ni_{15}$	1	Metallurgical	648	0.380	2.478	0.970	29160.774
[3]	$V_{85}Ni_{15}$	1	Metallurgical	648	0.388	2.672	1.018	19316.088
[3]	$V_{85}Ni_{15}$	1	Metallurgical	648	0.397	2.807	1.056	10921.052
[3]	$V_{85}Ni_{15}$	1	Metallurgical	648	0.403	2.956	1.091	2449.031
[3]	$V_{85}Ni_{15}$	1	Metallurgical	673	0.109	2.383	0.510	65706.633
[3]	$V_{85}Ni_{15}$	1	Metallurgical	673	0.194	2.250	0.661	68765.465

[3]	$V_{85}Ni_{15}$	1	Metallurgical	673	0.246	2.319	0.755	64390.173
[3]	$V_{85}Ni_{15}$	1	Metallurgical	673	0.278	2.448	0.825	58100.189
[3]	$V_{85}Ni_{15}$	1	Metallurgical	673	0.304	2.555	0.882	51028.208
[3]	$V_{85}Ni_{15}$	1	Metallurgical	673	0.320	2.692	0.929	43825.209
[3]	$V_{85}Ni_{15}$	1	Metallurgical	673	0.333	2.814	0.968	36815.385
[3]	$V_{85}Ni_{15}$	1	Metallurgical	673	0.347	2.937	1.009	28725.568
[3]	$V_{85}Ni_{15}$	1	Metallurgical	673	0.356	3.104	1.051	19597.485
[3]	$V_{85}Ni_{15}$	1	Metallurgical	673	0.359	3.412	1.107	5743.043

* data not fully reliable

Table S7 $V_{90}Ni_{10}$ solubility at different temperatures.

Reference	Alloy composition (at%)	Thickness (mm)	Preparation method	T (K)	Hydrogen content (H/M)	Solubility (MPa)	$P^{0.5}$ ($MPa^{0.5}$)	Solubility ($mol \cdot m^{-3} \cdot MPa^{-0.5}$)
[3]	$V_{90}Ni_{10}$	1	Metallurgical	623	0.176	1.220	0.463	117760.673
[3]	$V_{90}Ni_{10}$	1	Metallurgical	623	0.247	1.110	0.523	111350.787
[3]	$V_{90}Ni_{10}$	1	Metallurgical	623	0.357	1.250	0.668	80810.382
[3]	$V_{90}Ni_{10}$	1	Metallurgical	623	0.403	1.426	0.758	57971.780
[3]	$V_{90}Ni_{10}$	1	Metallurgical	623	0.430	1.595	0.828	42007.418
[3]	$V_{90}Ni_{10}$	1	Metallurgical	623	0.446	1.749	0.884	31871.282
[3]	$V_{90}Ni_{10}$	1	Metallurgical	623	0.462	1.872	0.930	26133.306
[3]	$V_{90}Ni_{10}$	1	Metallurgical	623	0.473	2.022	0.978	23508.542
[3]	$V_{90}Ni_{10}$	1	Metallurgical	623	0.483	2.171	1.024	24852.420
[3]	$V_{90}Ni_{10}$	1	Metallurgical	623	0.490	2.316	1.066	29835.755
[3]	$V_{90}Ni_{10}$	1	Metallurgical	623	0.502	2.502	1.120	42628.092
[3]	$V_{90}Ni_{10}$	1	Metallurgical	648	0.208	1.523	0.562	102264.419
[3]	$V_{90}Ni_{10}$	1	Metallurgical	648	0.270	1.477	0.632	91599.856
[3]	$V_{90}Ni_{10}$	1	Metallurgical	648	0.341	1.645	0.749	68046.540
[3]	$V_{90}Ni_{10}$	1	Metallurgical	648	0.379	1.808	0.828	51586.748

[3]	$V_{90}Ni_{10}$	1	Metallurgical	648	0.403	1.965	0.890	40385.421
[3]	$V_{90}Ni_{10}$	1	Metallurgical	648	0.417	2.103	0.936	34005.778
[3]	$V_{90}Ni_{10}$	1	Metallurgical	648	0.431	2.214	0.977	30066.746
[3]	$V_{90}Ni_{10}$	1	Metallurgical	648	0.440	2.383	1.024	28032.596
[3]	$V_{90}Ni_{10}$	1	Metallurgical	648	0.458	2.667	1.105	32574.244
[3]	$V_{90}Ni_{10}$	1	Metallurgical	673	0.076	2.577	0.444	50318.956
[3]	$V_{90}Ni_{10}$	1	Metallurgical	673	0.095	2.427	0.479	60172.900
[3]	$V_{90}Ni_{10}$	1	Metallurgical	673	0.117	2.205	0.509	67840.265
[3]	$V_{90}Ni_{10}$	1	Metallurgical	673	0.138	2.246	0.557	78101.975
[3]	$V_{90}Ni_{10}$	1	Metallurgical	673	0.160	2.175	0.591	83644.399
[3]	$V_{90}Ni_{10}$	1	Metallurgical	673	0.194	2.137	0.644	89005.472
[3]	$V_{90}Ni_{10}$	1	Metallurgical	673	0.210	2.005	0.648	89253.969
[3]	$V_{90}Ni_{10}$	1	Metallurgical	673	0.239	2.061	0.701	89877.451
[3]	$V_{90}Ni_{10}$	1	Metallurgical	673	0.298	2.113	0.793	80959.908
[3]	$V_{90}Ni_{10}$	1	Metallurgical	673	0.336	2.033	0.826	75025.636
[3]	$V_{90}Ni_{10}$	1	Metallurgical	673	0.360	2.127	0.875	64291.028
[3]	$V_{90}Ni_{10}$	1	Metallurgical	673	0.382	2.288	0.934	49411.543
[3]	$V_{90}Ni_{10}$	1	Metallurgical	673	0.395	2.412	0.977	38702.546

[3]	$V_{90}Ni_{10}$	1	Metallurgical	673	0.412	2.585	1.031	26327.999
[3]	$V_{90}Ni_{10}$	1	Metallurgical	673	0.422	2.747	1.077	19018.056

Table S8 $V_{95}Ni_5$ solubility at different temperatures.

Reference	Alloy composition (at%)	Thickness (mm)	Preparation method	T (K)	Hydrogen content (H/M)	Solubility (MPa)	$P^{0.5}$ ($MPa^{0.5}$)	Solubility ($mol\cdot m^{-3}\cdot MPa^{-0.5}$)
[3]	$V_{95}Ni_5$	1	Metallurgical	648	0.168	1.666	0.529	108829.621
[3]	$V_{95}Ni_5$	1	Metallurgical	648	0.183	1.581	0.538	108220.068
[3]	$V_{95}Ni_5$	1	Metallurgical	648	0.203	1.552	0.562	106250.890
[3]	$V_{95}Ni_5$	1	Metallurgical	648	0.228	1.506	0.587	103527.443
[3]	$V_{95}Ni_5$	1	Metallurgical	648	0.254	1.505	0.618	99325.611
[3]	$V_{95}Ni_5$	1	Metallurgical	648	0.285	1.520	0.658	92874.104
[3]	$V_{95}Ni_5$	1	Metallurgical	648	0.323	1.578	0.714	82551.829
[3]	$V_{95}Ni_5$	1	Metallurgical	648	0.354	1.646	0.763	72576.794
[3]	$V_{95}Ni_5$	1	Metallurgical	648	0.385	1.738	0.818	61521.415
[3]	$V_{95}Ni_5$	1	Metallurgical	648	0.409	1.856	0.871	51339.472
[3]	$V_{95}Ni_5$	1	Metallurgical	648	0.426	1.983	0.919	43279.558
[3]	$V_{95}Ni_5$	1	Metallurgical	648	0.440	2.084	0.958	37921.971
[3]	$V_{95}Ni_5$	1	Metallurgical	648	0.452	2.204	0.998	33811.499
[3]	$V_{95}Ni_5$	1	Metallurgical	648	0.464	2.316	1.036	31590.804
[3]	$V_{95}Ni_5$	1	Metallurgical	648	0.473	2.404	1.066	31120.267

[3]	$V_{95}Ni_5$	1	Metallurgical	648	0.483	2.487	1.096	31922.041
[3]	$V_{95}Ni_5$	1	Metallurgical	648	0.491	2.623	1.135	35049.620
[3]	$V_{95}Ni_5$	1	Metallurgical	648	0.502	2.788	1.183	42600.012
[3]	$V_{95}Ni_5$	1	Metallurgical	673	0.132	2.095	0.526	104125.104
[3]	$V_{95}Ni_5$	1	Metallurgical	673	0.149	1.927	0.536	103934.495
[3]	$V_{95}Ni_5$	1	Metallurgical	673	0.218	1.797	0.626	97673.773
[3]	$V_{95}Ni_5$	1	Metallurgical	673	0.250	1.786	0.668	92581.804
[3]	$V_{95}Ni_5$	1	Metallurgical	673	0.290	1.755	0.714	85927.241
[3]	$V_{95}Ni_5$	1	Metallurgical	673	0.326	1.820	0.770	76827.550
[3]	$V_{95}Ni_5$	1	Metallurgical	673	0.359	1.918	0.830	66650.871
[3]	$V_{95}Ni_5$	1	Metallurgical	673	0.385	2.013	0.880	58389.263
[3]	$V_{95}Ni_5$	1	Metallurgical	673	0.403	2.133	0.928	51316.068
[3]	$V_{95}Ni_5$	1	Metallurgical	673	0.423	2.186	0.961	47005.659
[3]	$V_{95}Ni_5$	1	Metallurgical	673	0.435	2.306	1.002	42777.359
[3]	$V_{95}Ni_5$	1	Metallurgical	673	0.448	2.395	1.036	40255.867
[3]	$V_{95}Ni_5$	1	Metallurgical	673	0.457	2.488	1.066	38978.944
[3]	$V_{95}Ni_5$	1	Metallurgical	673	0.469	2.581	1.100	38725.341
[3]	$V_{95}Ni_5$	1	Metallurgical	673	0.480	2.709	1.140	40227.204

Table S9 $V_{95}Ni_5$ solubility at different temperatures (deuterium).

Reference	Alloy composition (at%)	Thickness (mm)	Preparation method	T (K)	Hydrogen content (D/M)	Solubility (MPa)	$P^{0.5}$ ($MPa^{0.5}$)	Solubility ($mol \cdot m^{-3} \cdot MPa^{-0.5}$)
[4]	$V_{85}Ni_{15}$	5	Metallurgical	360	0.066	0.003	0.013	614619.280
[4]	$V_{85}Ni_{15}$	5	Metallurgical	360	0.083	0.003	0.017	611979.570
[4]	$V_{85}Ni_{15}$	5	Metallurgical	360	0.104	0.004	0.021	608926.443
[4]	$V_{85}Ni_{15}$	5	Metallurgical	360	0.128	0.005	0.025	605334.404
[4]	$V_{85}Ni_{15}$	5	Metallurgical	360	0.164	0.007	0.033	599725.385
[4]	$V_{85}Ni_{15}$	5	Metallurgical	360	0.246	0.010	0.050	586659.949
[4]	$V_{85}Ni_{15}$	5	Metallurgical	375	0.051	0.003	0.012	523720.527
[4]	$V_{85}Ni_{15}$	5	Metallurgical	375	0.070	0.004	0.018	541997.078
[4]	$V_{85}Ni_{15}$	5	Metallurgical	375	0.099	0.006	0.024	551762.548
[4]	$V_{85}Ni_{15}$	5	Metallurgical	375	0.141	0.008	0.033	442858.070
[4]	$V_{85}Ni_{15}$	5	Metallurgical	375	0.223	0.013	0.054	339122.474
[4]	$V_{85}Ni_{15}$	5	Metallurgical	375	0.358	0.062	0.149	108418.571
[4]	$V_{85}Ni_{15}$	5	Metallurgical	375	0.449	0.155	0.263	93361.378
[4]	$V_{85}Ni_{15}$	5	Metallurgical	375	0.492	0.227	0.334	91869.872
[4]	$V_{85}Ni_{15}$	5	Metallurgical	375	0.519	0.265	0.371	104235.325

[4]	$V_{85}Ni_{15}$	5	Metallurgical	375	0.55	0.280	0.395	111115.663
[4]	$V_{85}Ni_{15}$	5	Metallurgical	375	0.594	0.342	0.451	118379.802
[4]	$V_{85}Ni_{15}$	5	Metallurgical	375	0.665	0.412	0.524	100461.059*
[4]	$V_{85}Ni_{15}$	5	Metallurgical	375	0.734	0.609	0.669	33152.606*
[4]	$V_{85}Ni_{15}$	5	Metallurgical	390	0.047	0.005	0.014	421502.775
[4]	$V_{85}Ni_{15}$	5	Metallurgical	390	0.063	0.006	0.020	407463.394
[4]	$V_{85}Ni_{15}$	5	Metallurgical	390	0.093	0.008	0.027	387564.881
[4]	$V_{85}Ni_{15}$	5	Metallurgical	390	0.139	0.011	0.039	356426.485
[4]	$V_{85}Ni_{15}$	5	Metallurgical	390	0.224	0.027	0.078	261179.571
[4]	$V_{85}Ni_{15}$	5	Metallurgical	390	0.356	0.112	0.200	147444.897
[4]	$V_{85}Ni_{15}$	5	Metallurgical	390	0.481	0.294	0.376	80808.763
[4]	$V_{85}Ni_{15}$	5	Metallurgical	390	0.508	0.408	0.455	62226.316
[4]	$V_{85}Ni_{15}$	5	Metallurgical	390	0.536	0.440	0.485	57035.328
[4]	$V_{85}Ni_{15}$	5	Metallurgical	390	0.584	0.542	0.563	48501.895
[4]	$V_{85}Ni_{15}$	5	Metallurgical	390	0.642	0.683	0.662	47496.038
[4]	$V_{85}Ni_{15}$	5	Metallurgical	390	0.751	1.121	0.918	96573.846*
[4]	$V_{85}Ni_{15}$	5	Metallurgical	416	0.030	0.004	0.011	321261.220
[4]	$V_{85}Ni_{15}$	5	Metallurgical	416	0.046	0.007	0.019	308484.912

[4]	$V_{85}Ni_{15}$	5	Metallurgical	416	0.066	0.009	0.025	298125.046
[4]	$V_{85}Ni_{15}$	5	Metallurgical	416	0.093	0.015	0.037	277513.477
[4]	$V_{85}Ni_{15}$	5	Metallurgical	416	0.140	0.026	0.060	238259.051
[4]	$V_{85}Ni_{15}$	5	Metallurgical	416	0.218	0.054	0.108	156720.994
[4]	$V_{85}Ni_{15}$	5	Metallurgical	416	0.347	0.223	0.278	91408.025
[4]	$V_{85}Ni_{15}$	5	Metallurgical	416	0.491	0.806	0.629	31366.820
[4]	$V_{85}Ni_{15}$	5	Metallurgical	416	0.539	1.158	0.790	46501.338*
[4]	$V_{85}Ni_{15}$	5	Metallurgical	416	0.610	1.408	0.927	80633.151*
[4]	$V_{85}Ni_{15}$	5	Metallurgical	443	0.024	0.008	0.014	270642.232
[4]	$V_{85}Ni_{15}$	5	Metallurgical	443	0.031	0.008	0.016	268787.818
[4]	$V_{85}Ni_{15}$	5	Metallurgical	443	0.038	0.011	0.021	263008.818
[4]	$V_{85}Ni_{15}$	5	Metallurgical	443	0.044	0.014	0.025	258766.065
[4]	$V_{85}Ni_{15}$	5	Metallurgical	443	0.053	0.017	0.030	252775.076
[4]	$V_{85}Ni_{15}$	5	Metallurgical	443	0.066	0.021	0.037	245570.482
[4]	$V_{85}Ni_{15}$	5	Metallurgical	443	0.082	0.026	0.046	236216.735
[4]	$V_{85}Ni_{15}$	5	Metallurgical	443	0.101	0.032	0.057	224254.944
[4]	$V_{85}Ni_{15}$	5	Metallurgical	443	0.117	0.038	0.067	214509.590
[4]	$V_{85}Ni_{15}$	5	Metallurgical	443	0.137	0.045	0.079	202955.907

[4]	$V_{85}Ni_{15}$	5	Metallurgical	443	0.163	0.057	0.096	186664.209
[4]	$V_{85}Ni_{15}$	5	Metallurgical	443	0.193	0.079	0.124	162577.719
[4]	$V_{85}Ni_{15}$	5	Metallurgical	443	0.230	0.125	0.170	126827.275
[4]	$V_{85}Ni_{15}$	5	Metallurgical	443	0.273	0.201	0.234	85788.078
[4]	$V_{85}Ni_{15}$	5	Metallurgical	443	0.333	0.395	0.362	37770.515
[4]	$V_{85}Ni_{15}$	5	Metallurgical	443	0.386	0.719	0.527	40023.075*
[4]	$V_{85}Ni_{15}$	5	Metallurgical	443	0.436	1.212	0.727	139768.829*
[4]	$V_{85}Ni_{15}$	5	Metallurgical	473	0.014	0.025	0.018	122301.093
[4]	$V_{85}Ni_{15}$	5	Metallurgical	473	0.023	0.048	0.033	118181.045
[4]	$V_{85}Ni_{15}$	5	Metallurgical	473	0.034	0.045	0.039	116700.110
[4]	$V_{85}Ni_{15}$	5	Metallurgical	473	0.049	0.057	0.053	112999.407
[4]	$V_{85}Ni_{15}$	5	Metallurgical	473	0.068	0.079	0.073	107740.802
[4]	$V_{85}Ni_{15}$	5	Metallurgical	473	0.092	0.119	0.105	99962.323
[4]	$V_{85}Ni_{15}$	5	Metallurgical	473	0.128	0.156	0.141	91296.840
[4]	$V_{85}Ni_{15}$	5	Metallurgical	473	0.171	0.227	0.197	79230.603
[4]	$V_{85}Ni_{15}$	5	Metallurgical	473	0.213	0.370	0.281	63148.564
[4]	$V_{85}Ni_{15}$	5	Metallurgical	473	0.289	0.832	0.490	34780.550
[4]	$V_{85}Ni_{15}$	5	Metallurgical	473	0.380	2.172	0.909	28229.294

[4]	$V_{85}Ni_{15}$	5	Metallurgical	523	0.023	0.111	0.051	67733.118
[4]	$V_{85}Ni_{15}$	5	Metallurgical	523	0.031	0.109	0.059	67350.902
[4]	$V_{85}Ni_{15}$	5	Metallurgical	523	0.039	0.124	0.069	66816.555
[4]	$V_{85}Ni_{15}$	5	Metallurgical	523	0.062	0.233	0.120	64287.494
[4]	$V_{85}Ni_{15}$	5	Metallurgical	523	0.134	0.520	0.264	57085.718
[4]	$V_{85}Ni_{15}$	5	Metallurgical	523	0.196	0.816	0.400	50301.167
[4]	$V_{85}Ni_{15}$	5	Metallurgical	523	0.274	1.305	0.598	40406.175
[4]	$V_{85}Ni_{15}$	5	Metallurgical	523	0.353	2.475	0.935	23534.565
[4]	$V_{85}Ni_{15}$	5	Metallurgical	573	0.015	0.142	0.046	42890.946
[4]	$V_{85}Ni_{15}$	5	Metallurgical	573	0.027	0.240	0.080	43350.014
[4]	$V_{85}Ni_{15}$	5	Metallurgical	573	0.033	0.288	0.097	43534.261
[4]	$V_{85}Ni_{15}$	5	Metallurgical	573	0.041	0.373	0.124	43757.133
[4]	$V_{85}Ni_{15}$	5	Metallurgical	573	0.057	0.450	0.160	43928.374
[4]	$V_{85}Ni_{15}$	5	Metallurgical	573	0.088	0.675	0.244	43769.839
[4]	$V_{85}Ni_{15}$	5	Metallurgical	573	0.146	1.171	0.414	41052.915
[4]	$V_{85}Ni_{15}$	5	Metallurgical	573	0.212	1.935	0.641	32457.176
[4]	$V_{85}Ni_{15}$	5	Metallurgical	573	0.266	3.077	0.904	15318.971
[4]	$V_{85}Ni_{15}$	5	Metallurgical	623	0.020	0.405	0.090	26462.391

[4]	$V_{85}Ni_{15}$	5	Metallurgical	623	0.027	0.565	0.124	27252.054
[4]	$V_{85}Ni_{15}$	5	Metallurgical	623	0.035	0.754	0.163	28044.029
[4]	$V_{85}Ni_{15}$	5	Metallurgical	623	0.045	1.008	0.212	28882.927
[4]	$V_{85}Ni_{15}$	5	Metallurgical	623	0.064	1.355	0.295	29898.512
[4]	$V_{85}Ni_{15}$	5	Metallurgical	623	0.099	1.832	0.425	30492.387
[4]	$V_{85}Ni_{15}$	5	Metallurgical	623	0.138	2.498	0.587	29495.507
[4]	$V_{85}Ni_{15}$	5	Metallurgical	623	0.176	3.244	0.756	26423.038
[4]	$V_{85}Ni_{15}$	5	Metallurgical	623	0.205	3.971	0.902	22083.815
[4]	$V_{85}Ni_{15}$	5	Metallurgical	673	0.008	0.345	0.054	17559.236
[4]	$V_{85}Ni_{15}$	5	Metallurgical	673	0.019	0.867	0.127	18032.633
[4]	$V_{85}Ni_{15}$	5	Metallurgical	673	0.034	1.695	0.239	18759.623
[4]	$V_{85}Ni_{15}$	5	Metallurgical	673	0.064	2.940	0.434	20022.345
[4]	$V_{85}Ni_{15}$	5	Metallurgical	673	0.118	4.406	0.722	21890.027
[4]	$V_{85}Ni_{15}$	5	Metallurgical	673	0.147	5.607	0.909	23097.178

* data not fully reliable

Table S10 VFe alloys solubility at 673 K.

Reference	Alloy composition (at%)	Thickness (μm)	Preparation method	T (K)	Hydrogen content (H/M)	Solubility (MPa)	$P^{0.5}$ ($MPa^{0.5}$)	Solubility ($mol\cdot m^{-3}\cdot MPa^{-0.5}$)
[5]	V	2	ELP	400	0.016	0.064	0.032	82175.747
[5]	V	2	ELP	400	0.019	0.085	0.041	81569.632
[5]	V	2	ELP	400	0.026	0.083	0.047	81716.575
[5]	V	2	ELP	400	0.033	0.093	0.055	82674.351
[5]	V	2	ELP	400	0.045	0.111	0.070	85924.084
[5]	V	2	ELP	400	0.061	0.148	0.095	94540.813
[5]	V	2	ELP	400	0.068	0.159	0.104	98063.474
[5]	V	2	ELP	400	0.074	0.176	0.114	102571.743
[5]	V	2	ELP	400	0.081	0.183	0.122	105876.265
[5]	V	2	ELP	400	0.090	0.188	0.130	109364.991
[5]	V	2	ELP	400	0.100	0.197	0.140	113580.138
[5]	V	2	ELP	400	0.115	0.203	0.153	118426.369
[5]	V	2	ELP	400	0.130	0.219	0.168	123589.533
[5]	V	2	ELP	400	0.150	0.240	0.190	128740.719
[5]	V	2	ELP	400	0.175	0.272	0.218	131568.653

[5]	V	2	ELP	400	0.192	0.271	0.228	131361.165
[5]	V	2	ELP	400	0.215	0.281	0.246	129421.594
[5]	V	2	ELP	400	0.242	0.297	0.268	124383.363
[5]	V	2	ELP	400	0.265	0.343	0.302	112199.685
[5]	V	2	ELP	400	0.294	0.384	0.336	96901.582
[5]	V	2	ELP	400	0.335	0.437	0.383	79923.448
[5]	V	2	ELP	400	0.382	0.542	0.455	99784.645*
[5, 6]	V	2	ELP	400	0.024	0.052	0.036	81297.747
[5, 6]	V	2	ELP	400	0.032	0.078	0.050	93396.003
[5, 6]	V	2	ELP	400	0.035	0.079	0.053	95590.857
[5, 6]	V	2	ELP	400	0.043	0.092	0.063	103446.716
[5, 6]	V	2	ELP	400	0.057	0.110	0.079	113879.700
[5, 6]	V	2	ELP	400	0.079	0.130	0.101	125504.843
[5, 6]	V	2	ELP	400	0.091	0.153	0.118	132121.269
[5, 6]	V	2	ELP	400	0.123	0.174	0.146	139784.193
[5, 6]	V	2	ELP	400	0.138	0.192	0.163	142310.980
[5, 6]	V	2	ELP	400	0.170	0.202	0.186	143591.728
[5, 6]	V	2	ELP	400	0.189	0.212	0.200	143241.417

[5, 6]	V	2	ELP	400	0.249	0.243	0.246	136996.387
[5, 6]	V	2	ELP	400	0.306	0.285	0.295	123378.969
[5, 6]	V	2	ELP	400	0.344	0.343	0.344	105807.933
[5, 6]	V	2	ELP	400	0.392	0.407	0.399	83550.795
[5, 6]	V	2	ELP	400	0.423	0.520	0.469	58464.513
[5, 6]	V	2	ELP	400	0.470	0.678	0.564	42821.612
[5]	$V_{95}Fe_5$	2	ELP	400	0.009	0.151	0.037	28703.548
[5]	$V_{95}Fe_5$	2	ELP	400	0.019	0.303	0.076	31891.398
[5]	$V_{95}Fe_5$	2	ELP	400	0.029	0.453	0.115	34741.077
[5]	$V_{95}Fe_5$	2	ELP	400	0.037	0.528	0.140	36305.039
[5]	$V_{95}Fe_5$	2	ELP	400	0.045	0.601	0.165	37691.277
[5]	$V_{95}Fe_5$	2	ELP	400	0.096	1.174	0.336	42717.782
[5]	$V_{95}Fe_5$	2	ELP	400	0.152	1.454	0.469	41058.571
[5]	$V_{95}Fe_5$	2	ELP	400	0.239	3.094	0.860	8168.994
[5]	$V_{92.7}Fe_{7.3}$	2	ELP	400	0.006	0.280	0.042	15740.039
[5]	$V_{92.7}Fe_{7.3}$	2	ELP	400	0.024	1.137	0.165	20143.958
[5]	$V_{92.7}Fe_{7.3}$	2	ELP	400	0.029	1.362	0.200	21201.190
[5]	$V_{92.7}Fe_{7.3}$	2	ELP	400	0.062	2.221	0.370	25007.153

[5]	$V_{92.7}Fe_{7.3}$	2	ELP	400	0.095	2.761	0.512	26537.861
[5]	$V_{92.7}Fe_{7.3}$	2	ELP	400	0.126	3.485	0.664	26538.022
[5]	$V_{92.7}Fe_{7.3}$	2	ELP	400	0.173	4.469	0.878	23630.672
[5]	$V_{90.3}Fe_{9.7}$	2	ELP	400	0.008	0.518	0.064	14050.875
[5]	$V_{90.3}Fe_{9.7}$	2	ELP	400	0.012	0.807	0.097	14338.667
[5]	$V_{90.3}Fe_{9.7}$	2	ELP	400	0.024	1.504	0.190	15153.370
[5]	$V_{90.3}Fe_{9.7}$	2	ELP	400	0.046	2.711	0.355	16607.435
[5]	$V_{90.3}Fe_{9.7}$	2	ELP	400	0.066	3.975	0.512	17991.067
[5]	$V_{90.3}Fe_{9.7}$	2	ELP	400	0.092	4.761	0.664	19327.496
[5]	$V_{90.3}Fe_{9.7}$	2	ELP	400	0.142	6.455	0.957	21915.803
[5]	$V_{87.7}Fe_{12.3}$	2	ELP	400	0.002	0.303	0.025	11037.560
[5]	$V_{87.7}Fe_{12.3}$	2	ELP	400	0.004	0.520	0.048	11123.533
[5]	$V_{87.7}Fe_{12.3}$	2	ELP	400	0.007	0.708	0.071	11209.217
[5]	$V_{87.7}Fe_{12.3}$	2	ELP	400	0.012	1.212	0.121	11394.498
[5]	$V_{87.7}Fe_{12.3}$	2	ELP	400	0.019	2.118	0.200	11692.617
[5]	$V_{87.7}Fe_{12.3}$	2	ELP	400	0.036	3.870	0.374	12345.106
[5]	$V_{87.7}Fe_{12.3}$	2	ELP	400	0.050	5.768	0.535	12944.461
[5]	$V_{87.7}Fe_{12.3}$	2	ELP	400	0.068	6.505	0.664	13427.304

[5]	$V_{81.2}Fe_{18.8}$	2	ELP	400	0.001	0.748	0.030	50486.263
[5]	$V_{81.2}Fe_{18.8}$	2	ELP	400	0.001	0.862	0.035	50514.749
[5]	$V_{81.2}Fe_{18.8}$	2	ELP	400	0.003	1.664	0.067	50680.192
[5]	$V_{81.2}Fe_{18.8}$	2	ELP	400	0.005	2.293	0.106	50860.437
[5]	$V_{81.2}Fe_{18.8}$	2	ELP	400	0.006	2.746	0.124	50940.157
[5]	$V_{81.2}Fe_{18.8}$	2	ELP	400	0.007	2.929	0.142	51009.861
[5]	$V_{81.2}Fe_{18.8}$	2	ELP	400	0.009	3.924	0.186	51168.761
[5]	$V_{81.2}Fe_{18.8}$	2	ELP	400	0.010	4.546	0.209	51243.031
[5]	$V_{81.2}Fe_{18.8}$	2	ELP	400	0.011	5.321	0.243	51337.147
[5]	$V_{81.2}Fe_{18.8}$	2	ELP	400	0.015	6.603	0.318	51489.931
[5]	$V_{81.2}Fe_{18.8}$	2	ELP	400	0.016	8.510	0.366	51546.541
[5]	$V_{81.2}Fe_{18.8}$	2	ELP	400	0.020	8.990	0.426	51573.493
[5]	$V_{81.2}Fe_{18.8}$	2	ELP	400	0.026	12.698	0.577	51425.507
[5]	$V_{81.2}Fe_{18.8}$	2	ELP	400	0.038	14.489	0.739	50919.021
[5]	$V_{81.2}Fe_{18.8}$	2	ELP	400	0.045	21.611	0.989	49439.018

* data not fully reliable

Table S11 VFe alloys solubility at 623 K.

Reference	Alloy composition (at%)	Thickness (μm)	Preparation method	T (K)	Hydrogen content (H/M)	Solubility (MPa)	$P^{0.5}$ ($MPa^{0.5}$)	Solubility ($mol \cdot m^{-3} \cdot MPa^{-0.5}$)
[5]	V	2	ELP	350	0.011	0.027	0.018	207561.856
[5]	V	2	ELP	350	0.014	0.030	0.020	206663.844
[5]	V	2	ELP	350	0.041	0.041	0.041	199388.946
[5]	V	2	ELP	350	0.049	0.067	0.058	193587.075
[5]	V	2	ELP	350	0.114	0.091	0.102	178093.970
[5]	V	2	ELP	350	0.172	0.120	0.143	163641.892
[5]	V	2	ELP	350	0.234	0.128	0.173	153190.802
[5]	V	2	ELP	350	0.328	0.167	0.234	132087.327
[5]	V	2	ELP	350	0.440	0.340	0.386	78734.846
[5]	V	2	ELP	350	0.509	0.740	0.614	-636.432*
[5, 7]	V	2	ELP	350	0.018	0.019	0.018	83319.559
[5, 7]	V	2	ELP	350	0.026	0.027	0.027	110340.744
[5, 7]	V	2	ELP	350	0.049	0.043	0.046	162517.036
[5, 7]	V	2	ELP	350	0.063	0.049	0.055	183814.958
[5, 7]	V	2	ELP	350	0.100	0.064	0.080	224896.438

[5, 7]	V	2	ELP	350	0.191	0.084	0.127	257192.398
[5, 7]	V	2	ELP	350	0.238	0.094	0.149	254840.416
[5, 7]	V	2	ELP	350	0.305	0.093	0.169	245354.086
[5, 7]	V	2	ELP	350	0.385	0.117	0.212	204559.452
[5, 7]	V	2	ELP	350	0.415	0.147	0.247	160375.182
[5, 7]	V	2	ELP	350	0.459	0.194	0.299	88382.753
[5, 7]	V	2	ELP	350	0.509	0.230	0.342	36591.101
[5, 7]	V	2	ELP	350	0.509	0.319	0.403	2134.853
[5, 7]	V	2	ELP	350	0.509	0.407	0.455	31536.365
[5]	$V_{95}Fe_5$	2	ELP	350	0.003	0.047	0.012	52965.425
[5]	$V_{95}Fe_5$	2	ELP	350	0.008	0.065	0.023	53551.697
[5]	$V_{95}Fe_5$	2	ELP	350	0.014	0.097	0.037	54416.884
[5]	$V_{95}Fe_5$	2	ELP	350	0.031	0.183	0.076	57064.474
[5]	$V_{95}Fe_5$	2	ELP	350	0.045	0.230	0.102	58979.459
[5]	$V_{95}Fe_5$	2	ELP	350	0.064	0.311	0.141	61805.789
[5]	$V_{95}Fe_5$	2	ELP	350	0.128	0.529	0.261	67017.183
[5]	$V_{95}Fe_5$	2	ELP	350	0.193	0.751	0.381	60285.670
[5]	$V_{95}Fe_5$	2	ELP	350	0.224	0.900	0.449	47858.798

[5]	$V_{90.3}Fe_{9.7}$	2	ELP	350	0.005	0.166	0.029	24412.115
[5]	$V_{90.3}Fe_{9.7}$	2	ELP	350	0.016	0.465	0.086	24152.578
[5]	$V_{90.3}Fe_{9.7}$	2	ELP	350	0.033	1.007	0.183	23703.399
[5]	$V_{90.3}Fe_{9.7}$	2	ELP	350	0.065	1.696	0.333	23013.103
[5]	$V_{90.3}Fe_{9.7}$	2	ELP	350	0.096	2.690	0.507	22209.988
[5]	$V_{87.7}Fe_{12.3}$	2	ELP	350	0.002	0.134	0.016	17486.20226
[5]	$V_{87.7}Fe_{12.4}$	2	ELP	350	0.004	0.237	0.030	17539.624
[5]	$V_{87.7}Fe_{12.5}$	2	ELP	350	0.006	0.373	0.049	17606.405
[5]	$V_{87.7}Fe_{12.6}$	2	ELP	350	0.009	0.498	0.066	17667.957
[5]	$V_{87.7}Fe_{12.7}$	2	ELP	350	0.013	0.690	0.094	17769.042
[5]	$V_{87.7}Fe_{12.8}$	2	ELP	350	0.017	0.873	0.123	17874.715
[5]	$V_{87.7}Fe_{12.9}$	2	ELP	350	0.024	1.231	0.171	18046.153
[5]	$V_{87.7}Fe_{12.9}$	2	ELP	350	0.033	1.621	0.231	18260.956
[5]	$V_{87.7}Fe_{12.9}$	2	ELP	350	0.045	2.166	0.311	18550.618
[5]	$V_{87.7}Fe_{12.9}$	2	ELP	350	0.063	3.091	0.443	19026.316
[5]	$V_{87.7}Fe_{12.9}$	2	ELP	350	0.094	3.888	0.605	19611.894
[5]	$V_{87.7}Fe_{12.9}$	2	ELP	350	0.128	5.482	0.839	20452.691
[5]	$V_{81.2}Fe_{18.8}$	2	ELP	350	0.001	0.404	0.021	7063.288

[5]	$V_{81.2}Fe_{18.8}$	2	ELP	350	0.002	0.693	0.034	6994.040
[5]	$V_{81.2}Fe_{18.8}$	2	ELP	350	0.003	0.937	0.050	6913.373
[5]	$V_{81.2}Fe_{18.8}$	2	ELP	350	0.004	1.268	0.068	6829.773
[5]	$V_{81.2}Fe_{18.8}$	2	ELP	350	0.005	1.900	0.102	6694.132
[5]	$V_{81.2}Fe_{18.8}$	2	ELP	350	0.008	3.057	0.158	6532.134
[5]	$V_{81.2}Fe_{18.8}$	2	ELP	350	0.012	4.459	0.231	6430.793
[5]	$V_{81.2}Fe_{18.8}$	2	ELP	350	0.018	6.239	0.337	6508.998
[5]	$V_{81.2}Fe_{18.8}$	2	ELP	350	0.028	9.474	0.514	7230.107
[5]	$V_{81.2}Fe_{18.8}$	2	ELP	350	0.038	12.321	0.684	8619.040
[5]	$V_{81.2}Fe_{18.8}$	2	ELP	350	0.052	14.155	0.862	10799.036

* data not fully reliable

Table S12 VFe alloys solubility at 573 K.

Reference	Alloy composition (at%)	Thickness (μm)	Preparation method	T (K)	Hydrogen content (H/M)	Solubility (MPa)	$P^{0.5}$ ($MPa^{0.5}$)	Solubility ($mol \cdot m^{-3} \cdot MPa^{-0.5}$)
[5]	V	2	ELP	300	0.046	0.014	0.025	584568.955
[5]	V	2	ELP	300	0.323	0.038	0.111	275315.133
[5]	V	2	ELP	300	0.385	0.040	0.124	240740.496

[5]	V	2	ELP	300	0.419	0.051	0.147	191639.248
[5]	V	2	ELP	300	0.488	0.092	0.212	94340.200
[5]	V	2	ELP	300	0.505	0.119	0.245	66516.568
[5]	V	2	ELP	300	0.516	0.147	0.276	51010.342
[5]	V	2	ELP	300	0.544	0.183	0.316	41321.988
[5]	V	2	ELP	300	0.556	0.239	0.365	40046.606
[5]	V	2	ELP	300	0.575	0.316	0.426	42662.943
[5]	V	2	ELP	300	0.607	0.496	0.548	-800.777*
[5, 6]	V	2	ELP	300	0.019	0.007	0.011	493394.264
[5, 6]	V	2	ELP	300	0.028	0.009	0.015	485846.148
[5, 6]	V	2	ELP	300	0.041	0.012	0.022	474397.677
[5, 6]	V	2	ELP	300	0.056	0.014	0.028	462532.622
[5, 6]	V	2	ELP	300	0.076	0.020	0.039	442323.990
[5, 6]	V	2	ELP	300	0.119	0.021	0.050	421745.230
[5, 6]	V	2	ELP	300	0.143	0.025	0.060	405148.660
[5, 6]	V	2	ELP	300	0.198	0.026	0.072	383176.576
[5, 6]	V	2	ELP	300	0.233	0.030	0.084	361213.945
[5, 6]	V	2	ELP	300	0.287	0.029	0.091	347202.003

[5, 6]	V	2	ELP	300	0.397	0.034	0.116	301347.145
[5, 6]	V	2	ELP	300	0.483	0.071	0.185	175345.008
[5, 6]	V	2	ELP	300	0.516	0.110	0.238	78286.923
[5, 6]	V	2	ELP	300	0.550	0.152	0.289	-14908.355
[5]	$V_{95}Fe_5$	2	ELP	300	0.003	0.017	0.008	77784.389
[5]	$V_{95}Fe_5$	2	ELP	300	0.009	0.024	0.014	84210.848
[5]	$V_{95}Fe_5$	2	ELP	300	0.016	0.037	0.024	92417.363
[5]	$V_{95}Fe_5$	2	ELP	300	0.042	0.067	0.053	110390.904
[5]	$V_{95}Fe_5$	3	ELP	300	0.059	0.084	0.070	117194.157
[5]	$V_{95}Fe_5$	4	ELP	300	0.063	0.116	0.085	121106.802
[5]	$V_{95}Fe_5$	5	ELP	300	0.086	0.113	0.099	123215.211
[5]	$V_{95}Fe_5$	6	ELP	300	0.101	0.136	0.117	124295.391
[5]	$V_{95}Fe_5$	7	ELP	300	0.143	0.163	0.153	121658.984
[5]	$V_{95}Fe_5$	8	ELP	300	0.174	0.193	0.183	115833.649
[5]	$V_{95}Fe_5$	9	ELP	300	0.239	0.295	0.265	93165.428
[5]	$V_{95}Fe_5$	10	ELP	300	0.290	0.378	0.331	74722.402
[5]	$V_{95}Fe_5$	11	ELP	300	0.293	0.530	0.394	60761.196
[5]	$V_{95}Fe_5$	12	ELP	300	0.364	0.489	0.422	55973.214

[5]	$V_{95}Fe_5$	13	ELP	300	0.376	0.753	0.532	43517.608
[5]	$V_{95}Fe_5$	14	ELP	300	0.452	1.473	0.816	16361.259
[5]	$V_{95}Fe_5$	2	ELP	300	0.472	1.885	0.944	32903.384*
[5]	$V_{92.7}Fe_{7.3}$	2	ELP	300	0.007	0.089	0.025	52068.648
[5]	$V_{92.7}Fe_{7.3}$	2	ELP	300	0.022	0.162	0.060	51422.894
[5]	$V_{92.7}Fe_{7.3}$	2	ELP	300	0.035	0.352	0.111	50463.800
[5]	$V_{92.7}Fe_{7.3}$	2	ELP	300	0.050	0.365	0.136	49994.313
[5]	$V_{92.7}Fe_{7.3}$	2	ELP	300	0.059	0.509	0.173	49298.309
[5]	$V_{92.7}Fe_{7.3}$	2	ELP	300	0.133	0.844	0.334	46270.287
[5]	$V_{92.7}Fe_{7.3}$	2	ELP	300	0.182	1.262	0.479	43563.326
[5]	$V_{92.7}Fe_{7.3}$	2	ELP	300	0.244	1.714	0.646	40424.476
[5]	$V_{92.7}Fe_{7.3}$	2	ELP	300	0.303	2.377	0.849	36634.411
[5]	$V_{90.3}Fe_{9.7}$	2	ELP	300	0.003	0.073	0.015	40998.486
[5]	$V_{90.3}Fe_{9.7}$	2	ELP	300	0.009	0.158	0.038	40550.482
[5]	$V_{90.3}Fe_{9.7}$	2	ELP	300	0.021	0.297	0.079	39775.405
[5]	$V_{90.3}Fe_{9.7}$	2	ELP	300	0.043	0.587	0.159	38244.656
[5]	$V_{90.3}Fe_{9.7}$	2	ELP	300	0.086	1.051	0.301	35517.907
[5]	$V_{90.3}Fe_{9.7}$	2	ELP	300	0.140	1.485	0.456	25304.81848

[5]	$V_{90.3}Fe_{9.7}$	2	ELP	300	0.172	2.161	0.610	26189.184
[5]	$V_{90.3}Fe_{9.7}$	2	ELP	300	0.246	3.689	0.953	28163.107
[5]	$V_{87.7}Fe_{12.6}$	2	ELP	300	0.002	0.083	0.011	22747.467
[5]	$V_{87.7}Fe_{12.7}$	2	ELP	300	0.005	0.194	0.031	22859.941
[5]	$V_{87.7}Fe_{12.8}$	2	ELP	300	0.007	0.239	0.040	22915.118
[5]	$V_{87.7}Fe_{12.8}$	2	ELP	300	0.010	0.308	0.055	22996.617
[5]	$V_{87.7}Fe_{12.8}$	2	ELP	300	0.011	0.334	0.061	23032.060
[5]	$V_{87.7}Fe_{12.8}$	2	ELP	300	0.013	0.403	0.072	23098.585
[5]	$V_{87.7}Fe_{12.8}$	2	ELP	300	0.018	0.515	0.097	23238.799
[5]	$V_{87.7}Fe_{12.8}$	2	ELP	300	0.023	0.663	0.124	23398.048
[5]	$V_{87.7}Fe_{12.8}$	2	ELP	300	0.028	0.821	0.151	23550.959
[5]	$V_{87.7}Fe_{12.8}$	2	ELP	300	0.033	0.915	0.173	23677.060
[5]	$V_{87.7}Fe_{12.8}$	2	ELP	300	0.043	1.165	0.225	23974.902
[5]	$V_{87.7}Fe_{12.8}$	2	ELP	300	0.052	1.402	0.270	24236.747
[5]	$V_{87.7}Fe_{12.8}$	2	ELP	300	0.057	1.559	0.298	24394.824
[5]	$V_{87.7}Fe_{12.8}$	2	ELP	300	0.072	1.843	0.365	24780.120
[5]	$V_{87.7}Fe_{12.8}$	2	ELP	300	0.086	2.240	0.439	25205.154
[5]	$V_{87.7}Fe_{12.8}$	2	ELP	300	0.098	2.436	0.488	25490.096

[5]	$V_{87.7}Fe_{12.8}$	2	ELP	300	0.108	2.629	0.532	25744.446
[5]	$V_{87.7}Fe_{12.8}$	2	ELP	300	0.124	3.235	0.634	26327.732
[5]	$V_{87.7}Fe_{12.8}$	2	ELP	300	0.138	3.326	0.679	26585.293
[5]	$V_{87.7}Fe_{12.8}$	2	ELP	300	0.154	3.836	0.769	27107.045
[5]	$V_{87.7}Fe_{12.8}$	2	ELP	300	0.168	4.528	0.873	27703.185
[5]	$V_{87.7}Fe_{12.8}$	2	ELP	300	0.212	4.464	0.972	28270.331
[5]	$V_{81.2}Fe_{18.8}$	2	ELP	300	0.001	0.391	0.023	8606.086
[5]	$V_{81.2}Fe_{18.8}$	2	ELP	300	0.002	0.483	0.031	8626.621
[5]	$V_{81.2}Fe_{18.8}$	2	ELP	300	0.003	0.673	0.045	8662.858
[5]	$V_{81.2}Fe_{18.8}$	2	ELP	300	0.004	1.000	0.067	8717.566
[5]	$V_{81.2}Fe_{18.8}$	2	ELP	300	0.007	1.406	0.099	8797.983
[5]	$V_{81.2}Fe_{18.8}$	2	ELP	300	0.011	2.506	0.168	8974.221
[5]	$V_{81.2}Fe_{18.8}$	2	ELP	300	0.018	3.759	0.260	9207.260
[5]	$V_{81.2}Fe_{18.8}$	2	ELP	300	0.031	5.577	0.418	9606.425
[5]	$V_{81.2}Fe_{18.8}$	2	ELP	300	0.048	8.491	0.640	10168.249
[5]	$V_{81.2}Fe_{18.8}$	2	ELP	300	0.065	11.000	0.849	10691.870
[5]	$V_{81.2}Fe_{18.8}$	2	ELP	300	0.075	12.047	0.953	10954.474

* data not fully reliable

Table S13 VFe alloys solubility at 523 K.

Reference	Alloy composition (at%)	Thickness (μm)	Preparation method	T (K)	Hydrogen content (H/M)	Solubility (MPa)	$P^{0.5}$ ($MPa^{0.5}$)	Solubility ($mol\cdot m^{-3}\cdot MPa^{-0.5}$)
[5, 7]	V	2	ELP	250	0.034	0.003	0.010	1162161.544
[5, 7]	V	2	ELP	250	0.042	0.004	0.012	1130597.721
[5, 7]	V	2	ELP	250	0.047	0.004	0.013	1121820.027
[5, 7]	V	2	ELP	250	0.059	0.004	0.016	1078380.198
[5, 7]	V	2	ELP	250	0.073	0.005	0.019	1033381.164
[5, 7]	V	2	ELP	250	0.082	0.005	0.021	1004862.954
[5, 7]	V	2	ELP	250	0.126	0.006	0.027	913726.693
[5, 7]	V	2	ELP	250	0.153	0.007	0.032	854062.143
[5, 7]	V	2	ELP	250	0.212	0.007	0.037	788508.592
[5, 7]	V	2	ELP	250	0.259	0.008	0.044	711337.686
[5, 7]	V	2	ELP	250	0.322	0.008	0.050	645001.513
[5, 7]	V	2	ELP	250	0.384	0.010	0.062	532553.414
[5, 7]	V	2	ELP	250	0.422	0.013	0.075	427866.830
[5, 7]	V	2	ELP	250	0.445	0.014	0.079	396869.719
[5, 7]	V	2	ELP	250	0.526	0.036	0.138	123468.822

[5, 7]	V	2	ELP	250	0.566	0.060	0.184	47576.833
[5, 7]	V	2	ELP	250	0.584	0.090	0.229	32925.344
[5, 7]	V	2	ELP	250	0.615	0.130	0.283	41121.677
[5, 7]	V	2	ELP	250	0.621	0.192	0.345	40663.311
[5, 7]	V	2	ELP	250	0.641	0.265	0.412	17676.076
[5, 7]	V	2	ELP	250	0.662	0.400	0.514	69180.318
[5]	$V_{95}Fe_5$	2	ELP	250	0.009	0.013	0.011	231255.183
[5]	$V_{95}Fe_5$	2	ELP	250	0.015	0.026	0.020	225149.471
[5]	$V_{95}Fe_5$	2	ELP	250	0.039	0.041	0.040	211833.754
[5]	$V_{95}Fe_5$	2	ELP	250	0.061	0.040	0.049	206182.210
[5]	$V_{95}Fe_5$	3	ELP	250	0.093	0.044	0.064	196833.615
[5]	$V_{95}Fe_5$	4	ELP	250	0.113	0.041	0.068	194592.366
[5]	$V_{95}Fe_5$	5	ELP	250	0.146	0.059	0.093	179817.255
[5]	$V_{95}Fe_5$	6	ELP	250	0.219	0.089	0.139	153502.056
[5]	$V_{95}Fe_5$	7	ELP	250	0.287	0.137	0.199	123358.314
[5]	$V_{95}Fe_5$	8	ELP	250	0.357	0.219	0.280	87876.231
[5]	$V_{95}Fe_5$	9	ELP	250	0.393	0.414	0.403	46990.987
[5]	$V_{95}Fe_5$	10	ELP	250	0.393	0.405	0.399	48228.705

[5]	$V_{95}Fe_5$	11	ELP	250	0.427	0.531	0.476	30192.679
[5]	$V_{95}Fe_5$	12	ELP	250	0.484	0.995	0.694	12377.158
[5]	$V_{95}Fe_5$	13	ELP	250	0.484	1.356	0.810	22726.878
[5]	$V_{95}Fe_5$	14	ELP	250	0.520	1.757	0.956	55416.035
[5]	$V_{90.3}Fe_{9.7}$	2	ELP	250	0.002	0.020	0.007	57877.326
[5]	$V_{90.3}Fe_{9.7}$	2	ELP	250	0.005	0.041	0.014	57631.053
[5]	$V_{90.3}Fe_{9.7}$	2	ELP	250	0.013	0.083	0.033	56991.717
[5]	$V_{90.3}Fe_{9.7}$	2	ELP	250	0.029	0.165	0.069	55765.851
[5]	$V_{90.3}Fe_{9.7}$	2	ELP	250	0.058	0.302	0.132	53659.680
[5]	$V_{90.3}Fe_{9.7}$	2	ELP	250	0.117	0.670	0.280	38496.10006
[5]	$V_{90.3}Fe_{9.7}$	2	ELP	250	0.179	0.990	0.421	38895.910
[5]	$V_{90.3}Fe_{9.7}$	2	ELP	250	0.231	1.531	0.594	39383.599
[5]	$V_{87.7}Fe_{12.6}$	2	ELP	250	0.016	0.135	0.047	37836.791
[5]	$V_{87.7}Fe_{12.7}$	2	ELP	250	0.041	0.346	0.119	38042.409
[5]	$V_{87.7}Fe_{12.8}$	2	ELP	250	0.138	1.258	0.417	38883.431
[5]	$V_{87.7}Fe_{12.8}$	2	ELP	250	0.187	1.729	0.569	39311.512
[5]	$V_{87.7}Fe_{12.8}$	2	ELP	250	0.223	2.252	0.709	39709.298
[5]	$V_{87.7}Fe_{12.8}$	2	ELP	250	0.251	2.562	0.802	39969.431

[5]	$V_{87.7}Fe_{12.8}$	2	ELP	250	0.312	2.869	0.946	40378.132
[5]	$V_{81.2}Fe_{18.8}$	2	ELP	250	0.002	0.216	0.019	13118.477
[5]	$V_{81.2}Fe_{18.8}$	2	ELP	250	0.003	0.333	0.031	13005.915
[5]	$V_{81.2}Fe_{18.8}$	2	ELP	250	0.004	0.503	0.046	12870.005
[5]	$V_{81.2}Fe_{18.8}$	2	ELP	250	0.006	0.639	0.061	12737.576
[5]	$V_{81.2}Fe_{18.8}$	2	ELP	250	0.008	0.875	0.081	12575.337
[5]	$V_{81.2}Fe_{18.8}$	2	ELP	250	0.011	1.379	0.125	12244.401
[5]	$V_{81.2}Fe_{18.8}$	2	ELP	250	0.018	1.953	0.186	11850.905
[5]	$V_{81.2}Fe_{18.8}$	2	ELP	250	0.030	3.629	0.330	11241.256
[5]	$V_{81.2}Fe_{18.8}$	2	ELP	250	0.050	5.328	0.514	11121.237
[5]	$V_{81.2}Fe_{18.8}$	2	ELP	250	0.062	6.521	0.635	11440.772
[5]	$V_{81.2}Fe_{18.8}$	2	ELP	250	0.083	8.464	0.837	12684.746
[5]	$V_{81.2}Fe_{18.8}$	2	ELP	250	0.094	9.737	0.956	13827.644

Table S14 VFe alloys solubility at 473 K.

Reference	Alloy composition (at%)	Thickness (μm)	Preparation method	T (K)	Hydrogen content (H/M)	Solubility (MPa)	$P^{0.5}$ ($MPa^{0.5}$)	Solubility ($mol\cdot m^{-3}\cdot MPa^{-0.5}$)
[5]	V	2	ELP	200	0.147	0.003	0.021	2786185.159
[5]	V	2	ELP	200	0.243	0.003	0.026	2045879.938
[5]	V	2	ELP	200	0.346	0.004	0.036	592740.587
[5, 7]	V	2	ELP	200	0.096	0.001	0.010	2921728.122
[5, 7]	V	2	ELP	200	0.128	0.001	0.012	2718539.106
[5, 7]	V	2	ELP	200	0.159	0.001	0.014	2524226.340
[5, 7]	V	2	ELP	200	0.212	0.001	0.016	2334972.527
[5, 7]	V	2	ELP	200	0.266	0.001	0.018	2145923.422
[5, 7]	V	2	ELP	200	0.323	0.001	0.020	1990181.084
[5, 7]	V	2	ELP	200	0.376	0.001	0.023	1808622.727
[5, 7]	V	2	ELP	200	0.405	0.002	0.026	1565441.238
[5, 7]	V	2	ELP	200	0.450	0.002	0.031	1178122.067
[5, 7]	V	2	ELP	200	0.468	0.003	0.036	894437.411
[5, 7]	V	2	ELP	200	0.489	0.004	0.043	574196.473
[5, 7]	V	2	ELP	200	0.582	0.015	0.093	-253739.080

[5, 7]	V	2	ELP	200	0.627	0.038	0.154	219785.001
[5, 7]	V	2	ELP	200	0.651	0.061	0.199	-759338.533
[5]	$V_{95}Fe_5$	2	ELP	200	0.045	0.011	0.022	671022.517
[5]	$V_{95}Fe_5$	2	ELP	200	0.069	0.013	0.029	588367.051
[5]	$V_{95}Fe_5$	2	ELP	200	0.102	0.011	0.033	548426.960
[5]	$V_{95}Fe_5$	2	ELP	200	0.131	0.012	0.040	484996.604
[5]	$V_{95}Fe_5$	2	ELP	200	0.158	0.016	0.051	392347.369
[5]	$V_{95}Fe_5$	2	ELP	200	0.197	0.021	0.064	292847.687
[5]	$V_{95}Fe_5$	2	ELP	200	0.247	0.027	0.081	197084.066
[5]	$V_{95}Fe_5$	2	ELP	200	0.296	0.033	0.099	120324.521
[5]	$V_{95}Fe_5$	2	ELP	200	0.341	0.058	0.141	25994.521
[5]	$V_{95}Fe_5$	2	ELP	200	0.396	0.089	0.188	7512.132
[5]	$V_{95}Fe_5$	2	ELP	200	0.430	0.130	0.236	7134.416
[5]	$V_{87.7}Fe_{12.3}$	2	ELP	200	0.006	0.031	0.013	96436.75986
[5]	$V_{87.7}Fe_{12.4}$	2	ELP	200	0.011	0.037	0.020	95792.890
[5]	$V_{87.7}Fe_{12.5}$	2	ELP	200	0.020	0.046	0.030	94803.910
[5]	$V_{87.7}Fe_{12.6}$	2	ELP	200	0.032	0.068	0.047	93230.900
[5]	$V_{87.7}Fe_{12.7}$	2	ELP	200	0.075	0.130	0.099	88150.376

[5]	$V_{87.7}Fe_{12.8}$	2	ELP	200	0.172	0.307	0.230	75502.866
[5]	$V_{87.7}Fe_{12.9}$	2	ELP	200	0.252	0.598	0.388	60126.615
[5]	$V_{87.7}Fe_{12.9}$	2	ELP	200	0.311	1.040	0.569	42632.265
[5]	$V_{87.7}Fe_{12.9}$	2	ELP	200	0.402	2.176	0.936	7116.313
[5]	$V_{81.2}Fe_{18.8}$	2	ELP	200	0.006	0.326	0.044	16146.821
[5]	$V_{81.2}Fe_{18.8}$	2	ELP	200	0.013	0.675	0.093	16276.867
[5]	$V_{81.2}Fe_{18.8}$	2	ELP	200	0.023	1.251	0.169	16567.561
[5]	$V_{81.2}Fe_{18.8}$	2	ELP	200	0.055	2.897	0.400	18093.329
[5]	$V_{81.2}Fe_{18.8}$	2	ELP	200	0.089	4.389	0.626	20536.023
[5]	$V_{81.2}Fe_{18.8}$	2	ELP	200	0.130	5.448	0.842	23744.312
[5]	$V_{81.2}Fe_{18.8}$	2	ELP	200	0.147	5.965	0.936	25401.389

Table S15 VFe alloys solubility at 423 K.

Reference	Alloy composition (at%)	Thickness (μm)	Preparation method	T (K)	Hydrogen content (H/M)	Solubility (MPa)	$P^{0.5}$ ($MPa^{0.5}$)	Solubility ($mol \cdot m^{-3} \cdot MPa^{-0.5}$)
[5, 6]	V	2	ELP	150	0.026	0.000	0.001	9680447.001
[5, 6]	V	2	ELP	150	0.032	0.000	0.001	9524908.996
[5, 6]	V	2	ELP	150	0.043	0.000	0.002	9277521.800
[5, 6]	V	2	ELP	150	0.057	0.000	0.003	8864544.578
[5, 6]	V	2	ELP	150	0.081	0.000	0.003	8379607.967
[5, 6]	V	2	ELP	150	0.117	0.000	0.004	7975220.328
[5, 6]	V	2	ELP	150	0.177	0.000	0.005	7490106.088
[5, 6]	V	2	ELP	150	0.261	0.000	0.006	7039838.651
[5, 6]	V	2	ELP	150	0.377	0.000	0.007	6422611.707
[5, 6]	V	2	ELP	150	0.518	0.000	0.009	4889607.282
[5, 6]	V	2	ELP	150	0.548	0.000	0.012	3251712.591
[5, 6]	V	2	ELP	150	0.569	0.001	0.021	-2443750.110
[5]	$V_{90.3}Fe_{9.7}$	2	ELP	150	0.024	0.021	0.022	146729.944
[5]	$V_{90.3}Fe_{9.8}$	2	ELP	150	0.033	0.029	0.031	145364.855
[5]	$V_{90.3}Fe_{9.9}$	2	ELP	150	0.083	0.045	0.061	140619.898

[5]	$V_{90.3}Fe_{9.10}$	2	ELP	150	0.208	0.126	0.162	124657.994
[5]	$V_{90.3}Fe_{9.11}$	2	ELP	150	0.298	0.378	0.336	97154.250
[5]	$V_{90.3}Fe_{9.12}$	2	ELP	150	0.467	0.474	0.470	75790.138
[5]	$V_{90.3}Fe_{9.13}$	2	ELP	150	0.586	1.461	0.925	3830.497
[5]	$V_{81.2}Fe_{18.8}$	2	ELP	150	0.003	0.088	0.015	24055.376
[5]	$V_{81.2}Fe_{18.8}$	2	ELP	150	0.008	0.220	0.041	24527.512
[5]	$V_{81.2}Fe_{18.8}$	2	ELP	150	0.019	0.424	0.090	25409.178
[5]	$V_{81.2}Fe_{18.8}$	2	ELP	150	0.032	0.768	0.158	26630.169
[5]	$V_{81.2}Fe_{18.8}$	2	ELP	150	0.043	1.035	0.210	27573.463
[5]	$V_{81.2}Fe_{18.8}$	2	ELP	150	0.062	1.398	0.294	29098.542
[5]	$V_{81.2}Fe_{18.8}$	2	ELP	150	0.108	2.104	0.476	32388.516
[5]	$V_{81.2}Fe_{18.8}$	2	ELP	150	0.129	2.282	0.542	33583.515
[5]	$V_{81.2}Fe_{18.8}$	2	ELP	150	0.150	2.682	0.634	35238.489

B.1.3 Nb and Nb based alloys membranes

Table S16 Nb and NbRu solubility at the same temperature.

Reference	Alloy composition (at%)	Thickness (μm)	Preparation method	T (K)	Hydrogen content (H/M)	$P^{0.5}$ (MPa)	Cage cm^3/mol	Solubility ($mol \cdot m^{-3} \cdot MPa^{-0.5}$)
[8]	Nb	4000	Metallurgical	673	0.068	0.034	10.841	233085.468
[8]	Nb	4000	Metallurgical	673	0.088	0.042	10.841	277069.655
[8]	Nb	4000	Metallurgical	673	0.110	0.047	10.841	312962.824
[8]	Nb	4000	Metallurgical	673	0.133	0.054	10.841	374423.177
[8]	Nb	4000	Metallurgical	673	0.158	0.060	10.841	432672.817
[8]	Nb	4000	Metallurgical	673	0.192	0.067	10.841	515555.952
[8]	Nb	4000	Metallurgical	673	0.225	0.073	10.841	592957.814
[8]	Nb	4000	Metallurgical	673	0.275	0.079	10.841	695159.528
[8]	Nb	4000	Metallurgical	673	0.303	0.083	10.841	732827.139
[8]	Nb	4000	Metallurgical	673	0.358	0.089	10.841	679167.647
[8]	Nb	4000	Metallurgical	673	0.395	0.096	10.841	594052.181
[8]	Nb	4000	Metallurgical	673	0.430	0.100	10.841	545574.538
[8]	Nb	4000	Metallurgical	673	0.454	0.106	10.841	463958.136

[8]	Nb	4000	Metallurgical	673	0.494	0.115	10.841	375277.320
[8]	Nb	4000	Metallurgical	673	0.516	0.122	10.841	308790.343
[8]	Nb	4000	Metallurgical	673	0.535	0.125	10.841	283891.145
[8]	Nb	4000	Metallurgical	673	0.572	0.141	10.841	202420.896
[8]	Nb	4000	Metallurgical	673	0.601	0.160	10.841	125141.040
[8]	Nb	4000	Metallurgical	673	0.619	0.178	10.841	97189.698
[8]	Nb	4000	Metallurgical	673	0.648	0.206	10.841	65092.540
[8]	Nb	4000	Metallurgical	673	0.665	0.234	10.841	49004.006
[8]	Nb	4000	Metallurgical	673	0.678	0.263	10.841	40368.932
[8]	Nb	4000	Metallurgical	673	0.701	0.316	10.841	34612.281*
[8]	Nb	4000	Metallurgical	673	0.708	0.339	10.841	31548.579*
[8]	Nb	4000	Metallurgical	673	0.722	0.394	10.841	10141.828*
[8]	$Nb_{85}Ru_{15}$	4000	Metallurigical	673	0.006	0.067	10.436	10207.456
[8]	$Nb_{85}Ru_{15}$	4000	Metallurigical	673	0.011	0.103	10.436	11764.183
[8]	$Nb_{85}Ru_{15}$	4000	Metallurigical	673	0.013	0.131	10.436	12685.375
[8]	$Nb_{85}Ru_{15}$	4000	Metallurigical	673	0.016	0.152	10.436	13235.579
[8]	$Nb_{85}Ru_{15}$	4000	Metallurigical	673	0.021	0.188	10.436	13904.847
[8]	$Nb_{85}Ru_{15}$	4000	Metallurigical	673	0.026	0.221	10.436	14275.075

[8]	$Nb_{85}Ru_{15}$	4000	Metallurigical	673	0.031	0.245	10.436	14424.611
[8]	$Nb_{85}Ru_{15}$	4000	Metallurigical	673	0.037	0.297	10.436	14446.577
[8]	$Nb_{85}Ru_{15}$	4000	Metallurigical	673	0.045	0.340	10.436	14229.848
[8]	$Nb_{85}Ru_{15}$	4000	Metallurigical	673	0.052	0.394	10.436	13757.249
[8]	$Nb_{85}Ru_{15}$	4000	Metallurigical	673	0.061	0.447	10.436	13153.427
[8]	$Nb_{85}Ru_{15}$	4000	Metallurigical	673	0.068	0.517	10.436	12242.128
[8]	$Nb_{85}Ru_{15}$	4000	Metallurigical	673	0.078	0.598	10.436	11141.527
[8]	$Nb_{85}Ru_{15}$	4000	Metallurigical	673	0.089	0.688	10.436	9889.804
[8]	$Nb_{85}Ru_{15}$	4000	Metallurigical	673	0.107	0.908	10.436	5496.934
[8]	$Nb_{90}Ru_{10}$	4000	Metallurigical	673	0.012	0.032	10.571	32602.716
[8]	$Nb_{90}Ru_{10}$	4000	Metallurigical	673	0.024	0.067	10.571	36470.498
[8]	$Nb_{90}Ru_{10}$	4000	Metallurigical	673	0.033	0.090	10.571	38261.392
[8]	$Nb_{90}Ru_{10}$	4000	Metallurigical	673	0.039	0.109	10.571	39364.032
[8]	$Nb_{90}Ru_{10}$	4000	Metallurigical	673	0.046	0.124	10.571	40006.412
[8]	$Nb_{90}Ru_{10}$	4000	Metallurigical	673	0.055	0.142	10.571	40500.118
[8]	$Nb_{90}Ru_{10}$	4000	Metallurigical	673	0.059	0.158	10.571	40745.388
[8]	$Nb_{90}Ru_{10}$	4000	Metallurigical	673	0.066	0.176	10.571	40785.114
[8]	$Nb_{90}Ru_{10}$	4000	Metallurigical	673	0.079	0.194	10.571	40613.594

[8]	$Nb_{90}Ru_{10}$	4000	Metallurigical	673	0.089	0.220	10.571	40023.984
[8]	$Nb_{90}Ru_{10}$	4000	Metallurigical	673	0.098	0.250	10.571	38878.334
[8]	$Nb_{90}Ru_{10}$	4000	Metallurigical	673	0.113	0.283	10.571	37271.756
[8]	$Nb_{90}Ru_{10}$	4000	Metallurigical	673	0.128	0.320	10.571	35005.854
[8]	$Nb_{90}Ru_{10}$	4000	Metallurigical	673	0.142	0.360	10.571	32407.762
[8]	$Nb_{90}Ru_{10}$	4000	Metallurigical	673	0.161	0.414	10.571	28829.906
[8]	$Nb_{90}Ru_{10}$	4000	Metallurigical	673	0.175	0.477	10.571	25397.180
[8]	$Nb_{90}Ru_{10}$	4000	Metallurigical	673	0.197	0.554	10.571	23415.215
[8]	$Nb_{90}Ru_{10}$	4000	Metallurigical	673	0.216	0.629	10.571	25345.918
[8]	$Nb_{95}Ru_{5}$	4000	Metallurigical	673	0.027	0.033	10.706	90907.320
[8]	$Nb_{95}Ru_{5}$	4000	Metallurigical	673	0.057	0.062	10.706	116503.976
[8]	$Nb_{95}Ru_{5}$	4000	Metallurigical	673	0.080	0.082	10.706	126961.010
[8]	$Nb_{95}Ru_{5}$	4000	Metallurigical	673	0.103	0.100	10.706	132794.102
[8]	$Nb_{95}Ru_{5}$	4000	Metallurigical	673	0.129	0.114	10.706	134753.901
[8]	$Nb_{95}Ru_{5}$	4000	Metallurigical	673	0.150	0.130	10.706	134972.588
[8]	$Nb_{95}Ru_{5}$	4000	Metallurigical	673	0.169	0.142	10.706	133857.162
[8]	$Nb_{95}Ru_{5}$	4000	Metallurigical	673	0.188	0.152	10.706	132095.624
[8]	$Nb_{95}Ru_{5}$	4000	Metallurigical	673	0.203	0.166	10.706	128869.204

[8]	$Nb_{95}Ru_5$	4000	Metallurigical	673	0.238	0.192	10.706	120245.562
[8]	$Nb_{95}Ru_5$	4000	Metallurigical	673	0.253	0.203	10.706	115902.715
[8]	$Nb_{95}Ru_5$	4000	Metallurigical	673	0.278	0.226	10.706	105578.589
[8]	$Nb_{95}Ru_5$	4000	Metallurigical	673	0.305	0.250	10.706	94360.430
[8]	$Nb_{95}Ru_5$	4000	Metallurigical	673	0.335	0.288	10.706	76723.196
[8]	$Nb_{95}Ru_5$	4000	Metallurigical	673	0.362	0.324	10.706	61702.770
[8]	$Nb_{95}Ru_5$	4000	Metallurigical	673	0.390	0.365	10.706	47472.292
[8]	$Nb_{95}Ru_5$	4000	Metallurigical	673	0.411	0.408	10.706	37516.525
[8]	$Nb_{95}Ru_5$	4000	Metallurigical	673	0.436	0.479	10.706	30735.794
[8]	$Nb_{95}Ru_5$	4000	Metallurigical	673	0.456	0.554	10.706	29513.954
[8]	$Nb_{95}Ru_5$	4000	Metallurigical	673	0.481	0.638	10.706	13084.229*

* data not fully reliable

Table S17 Nb and $Nb_{95}W_5$ solubility at different temperatures.

Reference	Alloy composition (at%)	Thickness (μm)	Preparation method	T (K)	Hydrogen content (H/M)	Solubility (MPa)	$P^{0.5}$ (MPa)	Solubility ($mol \cdot m^{-3} \cdot MPa^{-0.5}$)
[8]	Nb	4000	Metallurgical	673	0.068	0.017	0.034	233085.468
[8]	Nb	4000	Metallurgical	673	0.088	0.020	0.042	277069.655
[8]	Nb	4000	Metallurgical	673	0.110	0.020	0.047	312962.824
[8]	Nb	4000	Metallurgical	673	0.133	0.022	0.054	374423.177
[8]	Nb	4000	Metallurgical	673	0.158	0.023	0.060	432672.817
[8]	Nb	4000	Metallurgical	673	0.192	0.023	0.067	515555.952
[8]	Nb	4000	Metallurgical	673	0.225	0.023	0.073	592957.814
[8]	Nb	4000	Metallurgical	673	0.275	0.023	0.079	695159.528
[8]	Nb	4000	Metallurgical	673	0.303	0.023	0.083	732827.139
[8]	Nb	4000	Metallurgical	673	0.358	0.022	0.089	679167.647
[8]	Nb	4000	Metallurgical	673	0.395	0.023	0.096	594052.181
[8]	Nb	4000	Metallurgical	673	0.430	0.023	0.100	545574.538
[8]	Nb	4000	Metallurgical	673	0.454	0.025	0.106	463958.136
[8]	Nb	4000	Metallurgical	673	0.494	0.027	0.115	375277.320
[8]	Nb	4000	Metallurgical	673	0.516	0.029	0.122	308790.343

[8]	Nb	4000	Metallurgical	673	0.535	0.029	0.125	283891.145
[8]	Nb	4000	Metallurgical	673	0.572	0.035	0.141	202420.896
[8]	Nb	4000	Metallurgical	673	0.601	0.043	0.160	125141.040
[8]	Nb	4000	Metallurgical	673	0.619	0.051	0.178	97189.698
[8]	Nb	4000	Metallurgical	673	0.648	0.066	0.206	65092.540
[8]	Nb	4000	Metallurgical	673	0.665	0.082	0.234	49004.006
[8]	Nb	4000	Metallurgical	673	0.678	0.102	0.263	40368.932
[8]	Nb	4000	Metallurgical	673	0.701	0.142	0.316	34612.281*
[8]	Nb	4000	Metallurgical	673	0.708	0.163	0.339	31548.579*
[8]	Nb	4000	Metallurgical	673	0.722	0.215	0.394	10141.828*
[8]	$Nb_{95}W_5$	4000	Metallurgcal	673	0.092	0.028	0.051	220819.113
[8]	$Nb_{95}W_5$	4000	Metallurgcal	673	0.130	0.039	0.071	254193.014
[8]	$Nb_{95}W_5$	4000	Metallurgcal	673	0.157	0.043	0.082	265362.752
[8]	$Nb_{95}W_5$	4000	Metallurgcal	673	0.182	0.046	0.091	270354.722
[8]	$Nb_{95}W_5$	4000	Metallurgcal	673	0.207	0.047	0.099	272055.937
[8]	$Nb_{95}W_5$	4000	Metallurgcal	673	0.225	0.049	0.105	272001.981
[8]	$Nb_{95}W_5$	4000	Metallurgcal	673	0.242	0.051	0.111	270662.517
[8]	$Nb_{95}W_5$	4000	Metallurgcal	673	0.258	0.051	0.115	269057.353

[8]	$Nb_{95}W_5$	4000	Metallurgcal	673	0.272	0.053	0.120	266250.498
[8]	$Nb_{95}W_5$	4000	Metallurgcal	673	0.285	0.053	0.123	263989.805
[8]	$Nb_{95}W_5$	4000	Metallurgcal	673	0.304	0.056	0.131	257642.000
[8]	$Nb_{95}W_5$	4000	Metallurgcal	673	0.323	0.057	0.135	253351.191
[8]	$Nb_{95}W_5$	4000	Metallurgcal	673	0.341	0.061	0.144	242972.575
[8]	$Nb_{95}W_5$	4000	Metallurgcal	673	0.357	0.064	0.151	233581.138
[8]	$Nb_{95}W_5$	4000	Metallurgcal	673	0.393	0.068	0.164	214164.966
[8]	$Nb_{95}W_5$	4000	Metallurgcal	673	0.421	0.074	0.177	191933.784
[8]	$Nb_{95}W_5$	4000	Metallurgcal	673	0.445	0.085	0.194	158660.660
[8]	$Nb_{95}W_5$	4000	Metallurgcal	673	0.468	0.094	0.210	129561.998
[8]	$Nb_{95}W_5$	4000	Metallurgcal	673	0.492	0.106	0.229	95052.885
[8]	$Nb_{95}W_5$	4000	Metallurgcal	673	0.515	0.127	0.256	56711.288
[8]	$Nb_{95}W_5$	4000	Metallurgcal	673	0.540	0.151	0.285	40714.726
[8]	$Nb_{95}W_5$	4000	Metallurgcal	673	0.556	0.191	0.326	92526.073*
[8]	$Nb_{95}W_5$	4000	Metallurgcal	723	0.046	0.037	0.041	85018.022
[8]	$Nb_{95}W_5$	4000	Metallurgcal	723	0.076	0.067	0.071	95380.877
[8]	$Nb_{95}W_5$	4000	Metallurgcal	723	0.098	0.087	0.092	114695.650
[8]	$Nb_{95}W_5$	4000	Metallurgcal	723	0.114	0.096	0.105	127843.295

[8]	$Nb_{95}W_5$	4000	Metallurgcal	723	0.132	0.101	0.116	139287.933
[8]	$Nb_{95}W_5$	4000	Metallurgcal	723	0.143	0.105	0.123	146329.632
[8]	$Nb_{95}W_5$	4000	Metallurgcal	723	0.158	0.111	0.132	155062.558
[8]	$Nb_{95}W_5$	4000	Metallurgcal	723	0.174	0.117	0.143	163128.366
[8]	$Nb_{95}W_5$	4000	Metallurgcal	723	0.192	0.121	0.152	169004.861
[8]	$Nb_{95}W_5$	4000	Metallurgcal	723	0.208	0.125	0.161	172904.405
[8]	$Nb_{95}W_5$	4000	Metallurgcal	723	0.231	0.134	0.176	176122.902
[8]	$Nb_{95}W_5$	4000	Metallurgcal	723	0.256	0.135	0.186	175659.350
[8]	$Nb_{95}W_5$	4000	Metallurgcal	723	0.281	0.141	0.199	171856.977
[8]	$Nb_{95}W_5$	4000	Metallurgcal	723	0.309	0.148	0.214	163356.302
[8]	$Nb_{95}W_5$	4000	Metallurgcal	723	0.341	0.160	0.234	145991.968
[8]	$Nb_{95}W_5$	4000	Metallurgcal	723	0.367	0.177	0.255	121717.320
[8]	$Nb_{95}W_5$	4000	Metallurgcal	723	0.402	0.198	0.282	89195.010
[8]	$Nb_{95}W_5$	4000	Metallurgcal	723	0.434	0.216	0.306	87554.638
[8]	$Nb_{95}W_5$	4000	Metallurgcal	723	0.480	0.273	0.362	69731.026
[8]	$Nb_{95}W_5$	4000	Metallurgcal	723	0.507	0.334	0.411	55667.501
[8]	$Nb_{95}W_5$	4000	Metallurgcal	723	0.589	0.654	0.621	21991.059
[8]	$Nb_{95}W_5$	4000	Metallurgcal	723	0.609	0.803	0.699	19341.269

[8]	$Nb_{95}W_5$	4000	Metallurgcal	723	0.630	1.068	0.821	17442.748
[8]	$Nb_{95}W_5$	4000	Metallurgcal	723	0.647	1.386	0.947	527.995*
[8]	$Nb_{95}W_5$	4000	Metallurgcal	773	0.035	0.099	0.059	63467.195
[8]	$Nb_{95}W_5$	4000	Metallurgcal	773	0.057	0.141	0.090	77904.984
[8]	$Nb_{95}W_5$	4000	Metallurgcal	773	0.071	0.164	0.108	84856.496
[8]	$Nb_{95}W_5$	4000	Metallurgcal	773	0.084	0.179	0.123	89856.275
[8]	$Nb_{95}W_5$	4000	Metallurgcal	773	0.097	0.192	0.137	93809.116
[8]	$Nb_{95}W_5$	4000	Metallurgcal	773	0.110	0.202	0.149	96841.791
[8]	$Nb_{95}W_5$	4000	Metallurgcal	773	0.124	0.217	0.164	99862.334
[8]	$Nb_{95}W_5$	4000	Metallurgcal	773	0.139	0.236	0.181	102681.542
[8]	$Nb_{95}W_5$	4000	Metallurgcal	773	0.157	0.254	0.199	104820.302
[8]	$Nb_{95}W_5$	4000	Metallurgcal	773	0.177	0.268	0.218	106120.153
[8]	$Nb_{95}W_5$	4000	Metallurgcal	773	0.206	0.275	0.238	106707.634
[8]	$Nb_{95}W_5$	4000	Metallurgcal	773	0.238	0.291	0.263	106246.547
[8]	$Nb_{95}W_5$	4000	Metallurgcal	773	0.268	0.315	0.291	104388.399
[8]	$Nb_{95}W_5$	4000	Metallurgcal	773	0.310	0.336	0.323	100726.508
[8]	$Nb_{95}W_5$	4000	Metallurgcal	773	0.350	0.371	0.361	94603.551
[8]	$Nb_{95}W_5$	4000	Metallurgcal	773	0.398	0.425	0.411	84133.752

[8]	$Nb_{95}W_5$	4000	Metallurgcal	773	0.436	0.494	0.464	71378.389
[8]	$Nb_{95}W_5$	4000	Metallurgcal	773	0.475	0.594	0.531	54304.625
[8]	$Nb_{95}W_5$	4000	Metallurgcal	773	0.566	1.179	0.817	12491.159
[8]	$Nb_{95}W_5$	4000	Metallurgcal	773	0.591	1.512	0.945	32602.416*

* data not fully reliable

Table S18 Nb and $Nb_{95}Ru_5$ solubility at different temperatures.

Reference	Alloy composition (at%)	Thickness (μm)	Preparation method	T (K)	Hydrogen content (H/M)	Solubility (MPa)	$P^{0.5}$ (MPa)	Solubility ($mol \cdot m^{-3} \cdot MPa^{-0.5}$)
[8]	Nb	4000	Metallurgical	673	0.068	0.017	0.034	233085.468
[8]	Nb	4000	Metallurgical	673	0.088	0.020	0.042	277069.655
[8]	Nb	4000	Metallurgical	673	0.110	0.020	0.047	312962.824
[8]	Nb	4000	Metallurgical	673	0.133	0.022	0.054	374423.177
[8]	Nb	4000	Metallurgical	673	0.158	0.023	0.060	432672.817
[8]	Nb	4000	Metallurgical	673	0.192	0.023	0.067	515555.952
[8]	Nb	4000	Metallurgical	673	0.225	0.023	0.073	592957.814
[8]	Nb	4000	Metallurgical	673	0.275	0.023	0.079	695159.528
[8]	Nb	4000	Metallurgical	673	0.303	0.023	0.083	732827.139
[8]	Nb	4000	Metallurgical	673	0.358	0.022	0.089	679167.647
[8]	Nb	4000	Metallurgical	673	0.395	0.023	0.096	594052.181
[8]	Nb	4000	Metallurgical	673	0.430	0.023	0.100	545574.538
[8]	Nb	4000	Metallurgical	673	0.454	0.025	0.106	463958.136
[8]	Nb	4000	Metallurgical	673	0.494	0.027	0.115	375277.320
[8]	Nb	4000	Metallurgical	673	0.516	0.029	0.122	308790.343

[8]	Nb	4000	Metallurgical	673	0.535	0.029	0.125	283891.145
[8]	Nb	4000	Metallurgical	673	0.572	0.035	0.141	202420.896
[8]	Nb	4000	Metallurgical	673	0.601	0.043	0.160	125141.040
[8]	Nb	4000	Metallurgical	673	0.619	0.051	0.178	97189.698
[8]	Nb	4000	Metallurgical	673	0.648	0.066	0.206	65092.540
[8]	Nb	4000	Metallurgical	673	0.665	0.082	0.234	49004.006
[8]	Nb	4000	Metallurgical	673	0.678	0.102	0.263	40368.932
[8]	Nb	4000	Metallurgical	673	0.701	0.142	0.316	34612.281*
[8]	Nb	4000	Metallurgical	673	0.708	0.163	0.339	31548.579*
[8]	Nb	4000	Metallurgical	673	0.722	0.215	0.394	10141.828*
[8]	$Nb_{95}Ru_5$	4000	Metallurgical	673	0.032	0.034	0.033	93607.884
[8]	$Nb_{95}Ru_5$	4000	Metallurgical	673	0.059	0.065	0.062	118269.567
[8]	$Nb_{95}Ru_5$	4000	Metallurgical	673	0.082	0.081	0.081	128125.103
[8]	$Nb_{95}Ru_5$	4000	Metallurgical	673	0.106	0.096	0.101	133254.117
[8]	$Nb_{95}Ru_5$	4000	Metallurgical	673	0.130	0.099	0.113	134623.501
[8]	$Nb_{95}Ru_5$	4000	Metallurgical	673	0.150	0.111	0.129	134425.082
[8]	$Nb_{95}Ru_5$	4000	Metallurgical	673	0.169	0.118	0.141	133057.886
[8]	$Nb_{95}Ru_5$	4000	Metallurgical	673	0.187	0.126	0.153	130721.417

[8]	$Nb_{95}Ru_5$	4000	Metallurgical	673	0.205	0.133	0.165	127851.799
[8]	$Nb_{95}Ru_5$	4000	Metallurgical	673	0.240	0.148	0.189	120101.518
[8]	$Nb_{95}Ru_5$	4000	Metallurgical	673	0.255	0.160	0.202	115053.028
[8]	$Nb_{95}Ru_5$	4000	Metallurgical	673	0.282	0.183	0.227	104419.269
[8]	$Nb_{95}Ru_5$	4000	Metallurgical	673	0.302	0.204	0.248	95063.863
[8]	$Nb_{95}Ru_5$	4000	Metallurgical	673	0.339	0.244	0.288	77687.299
[8]	$Nb_{95}Ru_5$	4000	Metallurgical	673	0.364	0.291	0.325	62679.840
[8]	$Nb_{95}Ru_5$	4000	Metallurgical	673	0.460	0.659	0.551	26156.763
[8]	$Nb_{95}Ru_5$	4000	Metallurgical	673	0.483	0.838	0.636	25623.166
[8]	$Nb_{95}Ru_5$	4000	Metallurgical	673	0.500	1.042	0.722	21087.138*
[8]	$Nb_{95}Ru_5$	4000	Metallurgical	673	0.522	1.318	0.830	8891.873*
[8]	$Nb_{95}Ru_5$	4000	Metallurgical	673	0.537	1.750	0.970	28658.305*
[8]	$Nb_{95}Ru_5$	4000	Metallurgical	723	0.035	0.064	0.047	66376.132
[8]	$Nb_{95}Ru_5$	4000	Metallurgical	723	0.045	0.092	0.065	71709.966
[8]	$Nb_{95}Ru_5$	4000	Metallurgical	723	0.050	0.112	0.075	74543.395
[8]	$Nb_{95}Ru_5$	4000	Metallurgical	723	0.057	0.120	0.083	76506.152
[8]	$Nb_{95}Ru_5$	4000	Metallurgical	723	0.061	0.132	0.090	78252.789
[8]	$Nb_{95}Ru_5$	4000	Metallurgical	723	0.067	0.137	0.096	79594.412

[8]	$Nb_{95}Ru_5$	4000	Metallurgical	723	0.074	0.144	0.104	81304.775
[8]	$Nb_{95}Ru_5$	4000	Metallurgical	723	0.083	0.151	0.112	82984.384
[8]	$Nb_{95}Ru_5$	4000	Metallurgical	723	0.089	0.170	0.123	84882.831
[8]	$Nb_{95}Ru_5$	4000	Metallurgical	723	0.099	0.183	0.135	86592.356
[8]	$Nb_{95}Ru_5$	4000	Metallurgical	723	0.112	0.186	0.144	87773.094
[8]	$Nb_{95}Ru_5$	4000	Metallurgical	723	0.123	0.206	0.159	89321.549
[8]	$Nb_{95}Ru_5$	4000	Metallurgical	723	0.138	0.216	0.173	90248.345
[8]	$Nb_{95}Ru_5$	4000	Metallurgical	723	0.153	0.232	0.189	90884.825
[8]	$Nb_{95}Ru_5$	4000	Metallurgical	723	0.175	0.249	0.209	90960.056
[8]	$Nb_{95}Ru_5$	4000	Metallurgical	723	0.193	0.274	0.230	90212.444
[8]	$Nb_{95}Ru_5$	4000	Metallurgical	723	0.218	0.293	0.252	88573.747
[8]	$Nb_{95}Ru_5$	4000	Metallurgical	723	0.247	0.324	0.283	85091.306
[8]	$Nb_{95}Ru_5$	4000	Metallurgical	723	0.279	0.379	0.325	78306.908
[8]	$Nb_{95}Ru_5$	4000	Metallurgical	723	0.309	0.409	0.355	72434.190
[8]	$Nb_{95}Ru_5$	4000	Metallurgical	723	0.341	0.493	0.410	60595.521
[8]	$Nb_{95}Ru_5$	4000	Metallurgical	723	0.430	0.879	0.615	26180.564
[8]	$Nb_{95}Ru_5$	4000	Metallurgical	723	0.458	1.036	0.689	23600.233
[8]	$Nb_{95}Ru_5$	4000	Metallurgical	723	0.483	1.357	0.809	25256.339*

[8]	$Nb_{95}Ru_5$	4000	Metallurgical	723	0.510	1.697	0.930	5504.336*
[8]	$Nb_{95}Ru_5$	4000	Metallurgical	773	0.021	0.112	0.048	41702.022
[8]	$Nb_{95}Ru_5$	4000	Metallurgical	773	0.025	0.183	0.067	44729.745
[8]	$Nb_{95}Ru_5$	4000	Metallurgical	773	0.034	0.180	0.078	46321.442
[8]	$Nb_{95}Ru_5$	4000	Metallurgical	773	0.036	0.195	0.084	47298.949
[8]	$Nb_{95}Ru_5$	4000	Metallurgical	773	0.044	0.210	0.097	49072.279
[8]	$Nb_{95}Ru_5$	4000	Metallurgical	773	0.049	0.241	0.109	50815.158
[8]	$Nb_{95}Ru_5$	4000	Metallurgical	773	0.056	0.251	0.119	52082.791
[8]	$Nb_{95}Ru_5$	4000	Metallurgical	773	0.062	0.272	0.130	53477.947
[8]	$Nb_{95}Ru_5$	4000	Metallurgical	773	0.070	0.306	0.146	55396.320
[8]	$Nb_{95}Ru_5$	4000	Metallurgical	773	0.079	0.320	0.159	56859.726
[8]	$Nb_{95}Ru_5$	4000	Metallurgical	773	0.091	0.345	0.177	58572.963
[8]	$Nb_{95}Ru_5$	4000	Metallurgical	773	0.104	0.390	0.201	60529.443
[8]	$Nb_{95}Ru_5$	4000	Metallurgical	773	0.118	0.415	0.222	61848.270
[8]	$Nb_{95}Ru_5$	4000	Metallurgical	773	0.136	0.458	0.249	63110.683
[8]	$Nb_{95}Ru_5$	4000	Metallurgical	773	0.156	0.487	0.276	63746.532
[8]	$Nb_{95}Ru_5$	4000	Metallurgical	773	0.180	0.541	0.312	63739.136
[8]	$Nb_{95}Ru_5$	4000	Metallurgical	773	0.210	0.617	0.360	62251.537

[8]	$Nb_{95}Ru_5$	4000	Metallurgical	773	0.242	0.666	0.402	59724.599
[8]	$Nb_{95}Ru_5$	4000	Metallurgical	773	0.271	0.759	0.454	55253.686
[8]	$Nb_{95}Ru_5$	4000	Metallurgical	773	0.306	0.874	0.517	48430.821
[8]	$Nb_{95}Ru_5$	4000	Metallurgical	773	0.343	1.030	0.595	39349.015
[8]	$Nb_{95}Ru_5$	4001	Metallurgical	773	0.407	1.543	0.792	23734.576
[8]	$Nb_{95}Ru_5$	4002	Metallurgical	773	0.439	1.905	0.914	27721.227*

* data not reliable

B.1.4 Ta and Ta based alloys membranes

Table S19 Ta and TaAl alloys solubility at 673 K.

Reference	Alloy composition (at%)	Thickness (μm)	Preparation method	T (K)	Hydrogen content (H/M)	Solubility (MPa)	$P^{0.5}$ ($MPa^{0.5}$)	Solubility ($mol \cdot m^{-3} \cdot MPa^{-0.5}$)
[9]	Ta	4000	Metallurgical	673	0.003	22.065	0.254	1071.557
[9]	Ta	4000	Metallurgical	673	0.003	23.994	0.274	1046.562
[9]	Ta	4000	Metallurgical	673	0.003	24.749	0.284	1032.869
[9]	Ta	4000	Metallurgical	673	0.003	26.019	0.299	1013.956
[9]	Ta	4000	Metallurgical	673	0.004	27.732	0.317	990.988
[9]	Ta	4000	Metallurgical	673	0.004	29.749	0.338	963.964
[9]	$Ta_{96.8}Al_{3.2}$	4000	Metallurgical	673	0.002	36.387	0.254	615.894
[9]	$Ta_{96.8}Al_{3.2}$	4000	Metallurgical	673	0.002	39.310	0.274	627.646
[9]	$Ta_{96.8}Al_{3.2}$	4000	Metallurgical	673	0.002	40.916	0.284	633.881
[9]	$Ta_{96.8}Al_{3.2}$	4000	Metallurgical	673	0.002	42.998	0.299	642.676
[9]	$Ta_{96.8}Al_{3.2}$	4000	Metallurgical	673	0.002	45.555	0.317	653.033
[9]	$Ta_{96.8}Al_{3.2}$	4000	Metallurgical	673	0.002	48.514	0.338	665.644
[9]	$Ta_{97.6}Al_{2.4}$	4000	Metallurgical	673	0.002	31.859	0.254	750.845

[9]	$Ta_{97.6}Al_{2.4}$	4000	Metallurgical	673	0.002	34.250	0.274	755.281
[9]	$Ta_{97.6}Al_{2.4}$	4000	Metallurgical	673	0.002	35.485	0.284	757.636
[9]	$Ta_{97.6}Al_{2.4}$	4000	Metallurgical	673	0.002	37.345	0.299	760.971
[9]	$Ta_{97.6}Al_{2.4}$	4000	Metallurgical	673	0.003	39.448	0.317	764.932
[9]	$Ta_{97.6}Al_{2.4}$	4000	Metallurgical	673	0.003	41.990	0.338	769.755
[9]	$Ta_{98.4}Al_{1.6}$	4000	Metallurgical	673	0.002	27.206	0.254	842.836
[9]	$Ta_{98.4}Al_{1.6}$	4000	Metallurgical	673	0.003	29.235	0.274	846.514
[9]	$Ta_{98.4}Al_{1.6}$	4000	Metallurgical	673	0.003	30.449	0.284	848.491
[9]	$Ta_{98.4}Al_{1.6}$	4000	Metallurgical	673	0.003	32.201	0.299	851.304
[9]	$Ta_{98.4}Al_{1.6}$	4000	Metallurgical	673	0.003	33.945	0.317	854.589
[9]	$Ta_{98.4}Al_{1.6}$	4000	Metallurgical	673	0.003	36.317	0.339	858.636
[9]	$Ta_{99}Al_1$	4000	Metallurgical	673	0.003	24.512	0.254	952.667
[9]	$Ta_{99}Al_1$	4000	Metallurgical	673	0.003	26.443	0.274	953.865
[9]	$Ta_{99}Al_1$	4000	Metallurgical	673	0.003	27.474	0.285	954.529
[9]	$Ta_{99}Al_1$	4000	Metallurgical	673	0.003	28.814	0.299	955.420
[9]	$Ta_{99}Al_1$	4000	Metallurgical	673	0.003	30.584	0.317	956.513
[9]	$Ta_{99}Al_1$	4000	Metallurgical	673	0.004	32.633	0.338	957.824

Table S20 Ta and TaAl alloys solubility at 773 K.

Reference	Alloy composition (at%)	Thickness (μm)	Preparation method	T (K)	Hydrogen content (H/M)	Solubility (MPa)	$P^{0.5}$ ($MPa^{0.5}$)	Solubility ($mol \cdot m^{-3} \cdot MPa^{-0.5}$)
[9]	Ta	4000	Metallurgical	773	0.001	50.275	0.256	468.294
[9]	Ta	4000	Metallurgical	773	0.001	52.915	0.269	468.546
[9]	Ta	4000	Metallurgical	773	0.001	56.228	0.286	468.870
[9]	Ta	4000	Metallurgical	773	0.002	58.998	0.300	469.135
[9]	$Ta_{96.8}Al_{3.2}$	4000	Metallurgical	773	0.001	74.272	0.256	308.125
[9]	$Ta_{96.8}Al_{3.2}$	4000	Metallurgical	773	0.001	78.154	0.269	313.560
[9]	$Ta_{96.8}Al_{3.2}$	4000	Metallurgical	773	0.001	83.236	0.286	320.590
[9]	$Ta_{96.8}Al_{3.2}$	4000	Metallurgical	773	0.001	87.115	0.300	326.239
[9]	$Ta_{97.6}Al_{2.4}$	4000	Metallurgical	773	0.001	68.386	0.256	314.926
[9]	$Ta_{97.6}Al_{2.4}$	4000	Metallurgical	773	0.001	72.255	0.269	328.634
[9]	$Ta_{97.6}Al_{2.4}$	4000	Metallurgical	773	0.001	76.791	0.286	346.670
[9]	$Ta_{97.6}Al_{2.4}$	4000	Metallurgical	773	0.001	80.524	0.300	361.178
[9]	$Ta_{98.4}Al_{1.6}$	4000	Metallurgical	773	0.001	61.515	0.255	375.212
[9]	$Ta_{98.4}Al_{1.6}$	4000	Metallurgical	773	0.001	64.853	0.269	374.620
[9]	$Ta_{98.4}Al_{1.6}$	4000	Metallurgical	773	0.001	69.000	0.286	373.850

[9]	$Ta_{98.4}Al_{1.6}$	4000	Metallurgical	773	0.001	72.512	0.300	373.228
[9]	$Ta_{99}Al_1$	4000	Metallurgical	773	0.001	57.421	0.255	367.994
[9]	$Ta_{99}Al_1$	4000	Metallurgical	773	0.001	60.670	0.269	388.393
[9]	$Ta_{99}Al_1$	4000	Metallurgical	773	0.001	64.605	0.286	414.315
[9]	$Ta_{99}Al_1$	4000	Metallurgical	773	0.001	67.601	0.300	435.366

Table S21 Ta and TaAl alloys solubility at 873 K.

Reference	Alloy composition (at%)	Thickness (μm)	Preparation method	T (K)	Hydrogen content (H/M)	Solubility (MPa)	$P^{0.5}$ ($MPa^{0.5}$)	Solubility ($mol \cdot m^{-3} \cdot MPa^{-0.5}$)
[9]	Ta	4000	Metallurgical	873	0.001	94.100	0.259	278.557
[9]	Ta	4000	Metallurgical	873	0.001	98.354	0.272	275.452
[9]	Ta	4000	Metallurgical	873	0.001	102.942	0.286	272.210
[9]	Ta	4000	Metallurgical	873	0.001	107.713	0.300	268.831
[9]	$Ta_{96.8}Al_{3.2}$	4000	Metallurgical	873	0.001	120.225	0.259	224.421
[9]	$Ta_{96.8}Al_{3.2}$	4000	Metallurgical	873	0.001	125.352	0.272	224.983
[9]	$Ta_{96.8}Al_{3.2}$	4000	Metallurgical	873	0.001	131.019	0.286	225.570
[9]	$Ta_{96.8}Al_{3.2}$	4000	Metallurgical	873	0.001	136.602	0.300	226.182
[9]	$Ta_{97.6}Al_{2.4}$	4000	Metallurgical	873	0.001	115.279	0.259	228.813
[9]	$Ta_{97.6}Al_{2.4}$	4000	Metallurgical	873	0.001	120.467	0.272	227.501
[9]	$Ta_{97.6}Al_{2.4}$	4000	Metallurgical	873	0.001	125.907	0.285	226.149
[9]	$Ta_{97.6}Al_{2.4}$	4000	Metallurgical	873	0.001	131.750	0.300	224.696
[9]	$Ta_{98.4}Al_{1.6}$	4000	Metallurgical	873	0.001	109.014	0.259	252.788
[9]	$Ta_{98.4}Al_{1.6}$	4000	Metallurgical	873	0.001	113.714	0.272	244.910

[9]	$Ta_{98.4}Al_{1.6}$	4000	Metallurgical	873	0.001	118.917	0.286	236.684
[9]	$Ta_{98.4}Al_{1.6}$	4000	Metallurgical	873	0.001	124.489	0.300	228.109
[9]	$Ta_{99}Al_{1}$	4000	Metallurgical	873	0.001	101.510	0.259	255.861
[9]	$Ta_{99}Al_{1}$	4000	Metallurgical	873	0.001	106.110	0.272	257.132
[9]	$Ta_{99}Al_{1}$	4000	Metallurgical	873	0.001	110.975	0.286	258.460
[9]	$Ta_{99}Al_{1}$	4000	Metallurgical	873	0.001	115.934	0.300	259.844

Table S22 Ta and TaAl alloys Sieverts' costant at different temperatures.

Reference	Alloy composition (at%)	Support	Thickness (μm)	Preparation method	T (K)	P (MPa)	Ks*10^{-2} (kPa)$^{0.5}$
[9]	Ta	-	4000	Metallurgical	673	101.325	36.8
[9]	Ta	-	4000	Metallurgical	773	101.325	16.1
[9]	Ta	-	4000	Metallurgical	873	101.325	8.5
[9]	$Ta_{99}Al_1$	-	4000	Metallurgical	673	101.325	33.1
[9]	$Ta_{99}Al_1$	-	4000	Metallurgical	773	101.325	14.1
[9]	$Ta_{99}Al_1$	-	4000	Metallurgical	873	101.325	7.9
[9]	$T_{98.4}Al_{1.6}$	-	4000	Metallurgical	673	101.325	29.8
[9]	$T_{98.4}Al_{1.6}$	-	4000	Metallurgical	773	101.325	13.1
[9]	$T_{98.4}Al_{1.6}$	-	4000	Metallurgical	873	101.325	7.4
[9]	$Ta_{97.6}Al_{2.4}$	-	4000	Metallurgical	673	101.325	25.6
[9]	$Ta_{97.6}Al_{2.4}$	-	4000	Metallurgical	773	101.325	11.8
[9]	$Ta_{97.6}Al_{2.4}$	-	4000	Metallurgical	873	101.325	7.1
[9]	$Ta_{96.8}Al_{3.2}$	-	4000	Metallurgical	673	101.325	22.2
[9]	$Ta_{96.8}Al_{3.2}$	-	4000	Metallurgical	773	101.325	10.9
[9]	$Ta_{96.8}Al_{3.2}$	-	4000	Metallurgical	873	101.325	6.8

B.1.5 Amorphous alloys membranes

Table S23 NiNbZr based alloys solubility at 673 K.

Reference	Alloy composition (at%)	Thickness (μm)	Preparation method	T (K)	Hydrogen content (H/M)	Solubility (MPa)	$P^{0.5}$ ($MPa^{0.5}$)	Solubility ($mol \cdot m^{-3} \cdot MPa^{-0.5}$)
[10]	$(Ni_{0.6}Nb_{0.3}Ta_{0.1})_{70}Zr_{30}$	~30–70	Metallurgical	673	0.134	0.169	0.151	50276.538
[10]	$(Ni_{0.6}Nb_{0.3}Ta_{0.1})_{70}Zr_{30}$	~30–70	Metallurgical	673	0.177	0.381	0.260	54702.040
[10]	$(Ni_{0.6}Nb_{0.3}Ta_{0.1})_{70}Zr_{30}$	~30–70	Metallurgical	673	0.224	0.457	0.320	82628.872
[10]	$(Ni_{0.6}Nb_{0.3}Ta_{0.1})_{70}Zr_{30}$	~30–70	Metallurgical	673	0.261	0.477	0.353	105278.885
[10]	$(Ni_{0.6}Nb_{0.3}Ta_{0.1})_{70}Zr_{30}$	~30–70	Metallurgical	673	0.282	0.492	0.372	121644.551
[10]	$(Ni_{0.6}Nb_{0.3}Ta_{0.1})_{70}Zr_{30}$	~30–70	Metallurgical	673	0.311	0.505	0.397	144195.715
[10]	$(Ni_{0.6}Nb_{0.3}Ta_{0.1})_{70}Zr_{30}$	~30–70	Metallurgical	673	0.331	0.506	0.410	157708.045
[10]	$(Ni_{0.6}Nb_{0.3}Ta_{0.1})_{70}Zr_{30}$	~30–70	Metallurgical	673	0.350	0.506	0.421	170348.426
[10]	$(Ni_{0.6}Nb_{0.3}Ta_{0.1})_{70}Zr_{30}$	~30–70	Metallurgical	673	0.366	0.506	0.430	180392.371
[10]	$(Ni_{0.6}Nb_{0.3}Ta_{0.1})_{70}Zr_{30}$	~30–70	Metallurgical	673	0.382	0.503	0.438	190222.897
[10]	$(Ni_{0.6}Nb_{0.3}Ta_{0.1})_{70}Zr_{30}$	~30–70	Metallurgical	673	0.392	0.498	0.442	194494.097
[10]	$(Ni_{0.6}Nb_{0.4})_{70}Zr_{30}$	~30–70	Metallurgical	673	0.195	0.375	0.270	60778.617
[10]	$(Ni_{0.6}Nb_{0.4})_{70}Zr_{30}$	~30–70	Metallurgical	673	0.237	0.466	0.332	82670.720
[10]	$(Ni_{0.6}Nb_{0.4})_{70}Zr_{30}$	~30–70	Metallurgical	673	0.319	0.516	0.406	126562.379

[10]	$(Ni_{0.6}Nb_{0.4})_{70}Zr_{30}$	~30–70	Metallurgical	673	0.333	0.526	0.419	136118.183
[10]	$(Ni_{0.6}Nb_{0.4})_{70}Zr_{30}$	~30–70	Metallurgical	673	0.341	0.529	0.425	140605.474
[10]	$(Ni_{0.6}Nb_{0.4})_{70}Zr_{30}$	~30–70	Metallurgical	673	0.348	0.530	0.430	144616.926
[10]	$(Ni_{0.6}Nb_{0.4})_{70}Zr_{30}$	~30–70	Metallurgical	673	0.355	0.531	0.434	148402.032
[10]	$(Ni_{0.6}Nb_{0.4})_{70}Zr_{30}$	~30–70	Metallurgical	673	0.362	0.529	0.438	151466.228
[10]	$(Ni_{0.6}Nb_{0.4})_{80}Zr_{20}$	~30–70	Metallurgical	673	0.051	0.475	0.155	18089.102
[10]	$(Ni_{0.6}Nb_{0.4})_{80}Zr_{20}$	~30–70	Metallurgical	673	0.072	0.895	0.253	31699.534
[10]	$(Ni_{0.6}Nb_{0.4})_{80}Zr_{20}$	~30–70	Metallurgical	673	0.089	1.012	0.300	51881.898
[10]	$(Ni_{0.6}Nb_{0.4})_{80}Zr_{20}$	~30–70	Metallurgical	673	0.115	1.005	0.340	76630.840
[10]	$(Ni_{0.6}Nb_{0.4})_{80}Zr_{20}$	~30–70	Metallurgical	673	0.148	0.959	0.376	105353.802
[10]	$(Ni_{0.6}Nb_{0.4})_{80}Zr_{20}$	~30–70	Metallurgical	673	0.172	0.930	0.400	127321.196
[10]	$(Ni_{0.6}Nb_{0.4})_{80}Zr_{20}$	~30–70	Metallurgical	673	0.201	0.884	0.421	150213.659
[10]	$(Ni_{0.6}Nb_{0.4})_{80}Zr_{20}$	~30–70	Metallurgical	673	0.230	0.837	0.439	170914.198
[10]	$(Ni_{0.6}Nb_{0.4})_{90}Zr_{10}$	~30–70	Metallurgical	673	0.001	17.559	0.151	14560.566
[10]	$(Ni_{0.6}Nb_{0.4})_{90}Zr_{10}$	~30–70	Metallurgical	673	0.043	2.386	0.322	50277.675
[10]	$(Ni_{0.6}Nb_{0.4})_{90}Zr_{10}$	~30–70	Metallurgical	673	0.088	1.757	0.394	141022.395
[10]	$(Ni_{0.6}Nb_{0.4})_{90}Zr_{10}$	~30–70	Metallurgical	673	0.127	1.399	0.421	195125.010
[10]	$(Ni_{0.6}Nb_{0.4})_{90}Zr_{10}$	~30–70	Metallurgical	673	0.167	1.154	0.439	237869.672

Table S24 $(Ni_{0.42}Nb_{0.28}Zr_{0.30})_{98}B_2$ solubility at different temperatures.

Reference	Alloy composition (at%)	Thickness (μm)	Preparation method	T (K)	Hydrogen content (H/M)	Solubility (MPa)	$P^{0.5}$ ($MPa^{0.5}$)	Solubility ($mol \cdot m^{-3} \cdot MPa^{-0.5}$)
[10]	$(Ni_{0.42}Nb_{0.28}Zr_{0.30})_{98}B_2$	~30-70	Metallurgical	713	0.087	1.088	0.307	15650.899
[10]	$(Ni_{0.42}Nb_{0.28}Zr_{0.30})_{98}B_2$	~30-70	Metallurgical	713	0.152	3.010	0.677	30505.934
[10]	$(Ni_{0.42}Nb_{0.28}Zr_{0.30})_{98}B_2$	~30-70	Metallurgical	713	0.198	3.176	0.794	47328.455
[10]	$(Ni_{0.42}Nb_{0.28}Zr_{0.30})_{98}B_2$	~30-70	Metallurgical	713	0.244	3.087	0.869	61054.240
[10]	$(Ni_{0.42}Nb_{0.28}Zr_{0.30})_{98}B_2$	~30-70	Metallurgical	713	0.279	3.061	0.924	72770.236
[10]	$(Ni_{0.42}Nb_{0.28}Zr_{0.30})_{98}B_2$	~30-70	Metallurgical	713	0.303	3.036	0.960	81017.086
[10]	$(Ni_{0.42}Nb_{0.28}Zr_{0.30})_{98}B_2$	~30-70	Metallurgical	733	0.074	1.418	0.324	12140.392
[10]	$(Ni_{0.42}Nb_{0.28}Zr_{0.30})_{98}B_2$	~30-70	Metallurgical	733	0.135	3.407	0.678	32592.451
[10]	$(Ni_{0.42}Nb_{0.28}Zr_{0.30})_{98}B_2$	~30-70	Metallurgical	733	0.188	3.395	0.800	52221.478
[10]	$(Ni_{0.42}Nb_{0.28}Zr_{0.30})_{98}B_2$	~30-70	Metallurgical	733	0.228	3.242	0.860	64378.582
[10]	$(Ni_{0.42}Nb_{0.28}Zr_{0.30})_{98}B_2$	~30-70	Metallurgical	733	0.266	3.167	0.917	77212.068
[10]	$(Ni_{0.42}Nb_{0.28}Zr_{0.30})_{98}B_2$	~30-70	Metallurgical	733	0.296	3.090	0.956	86857.033
[10]	$(Ni_{0.42}Nb_{0.28}Zr_{0.30})_{98}B_2$	~30-70	Metallurgical	743	0.056	0.783	0.209	15676.866
[10]	$(Ni_{0.42}Nb_{0.28}Zr_{0.30})_{98}B_2$	~30-70	Metallurgical	743	0.125	3.689	0.680	31659.694
[10]	$(Ni_{0.42}Nb_{0.28}Zr_{0.30})_{98}B_2$	~30-70	Metallurgical	743	0.178	3.594	0.799	51484.183

[10]	$(Ni_{0.42}Nb_{0.28}Zr_{0.30})_{98}B_2$	~30-70	Metallurgical	743	0.216	3.412	0.859	63874.633
[10]	$(Ni_{0.42}Nb_{0.28}Zr_{0.30})_{98}B_2$	~30-70	Metallurgical	743	0.260	3.286	0.925	79394.839
[10]	$(Ni_{0.42}Nb_{0.28}Zr_{0.30})_{98}B_2$	~30-70	Metallurgical	743	0.285	3.207	0.956	87563.538
[10]	$(Ni_{0.42}Nb_{0.28}Zr_{0.30})_{98}B_2$	~30-70	Metallurgical	758	0.048	1.611	0.278	10483.654
[10]	$(Ni_{0.42}Nb_{0.28}Zr_{0.30})_{98}B_2$	~30-70	Metallurgical	758	0.116	3.979	0.679	33282.820
[10]	$(Ni_{0.42}Nb_{0.28}Zr_{0.30})_{98}B_2$	~30-70	Metallurgical	758	0.169	3.813	0.804	52178.879
[10]	$(Ni_{0.42}Nb_{0.28}Zr_{0.30})_{98}B_2$	~30-70	Metallurgical	758	0.209	3.582	0.866	63722.304
[10]	$(Ni_{0.42}Nb_{0.28}Zr_{0.30})_{98}B_2$	~30-70	Metallurgical	758	0.244	3.462	0.920	74829.179
[10]	$(Ni_{0.42}Nb_{0.28}Zr_{0.30})_{98}B_2$	~30-70	Metallurgical	758	0.272	3.357	0.956	82801.840

Table S25 ZrNi based alloys solubility at different temperatures.

Reference	Alloy composition (at%)	Thickness (μm)	Preparation method	T (K)	P (MPa)	P ($MPa^{0.5}$)	Hydrogen content (H/M)	Solubility (MPa)
[10]	$(Ni_{0.6}Nb_{0.4})_{90}Zr_{10}$	~30-70	Metallurgical	673	0.700	0.837	0.160	4.375
[11]	$(Ni_{0.6}Nb_{0.4})_{80}Zr_{20}$	~30-70	Metallurgical	673	0.700	0.837	0.240	2.917
[11]	$(Ni_{0.6}Nb_{0.4})_{70}Zr_{30}$	~30-70	Metallurgical	673	0.700	0.837	0.360	1.944
[10]	$(Ni_{0.42}Nb_{0.28}Zr_{0.30})_{98}B_2$	~30-70	Metallurgical	713	0.700	0.837	0.310	2.258

B.1.6 Liquid Ga membranes

Table S26 Liquid gallium solubility.

Reference	Alloy composition (at%)	Thickness (μm)	Preparation method	P (MPa)	10000/T (K^{-1})	T (K)	Hydrogen content (H/M)	Solubility (MPa)
[12]	Liquid gallium	200	-	0.100	12.9146	774.319	0.048	2.084
[12]	Liquid gallium	200	-	0.100	14.8397	673.869	0.030	3.279
[12]	Liquid gallium	200	-	0.100	16.0297	623.842	0.026	3.846

REFERENCES

[1] Paolone A., Tosti S., Santucci A., Palumbo., Trequattrini F., 2017. Hydrogen and deuterium solubility in commercial Pd-Ag alloys for hydrogen purification. *Chemengineering.* **1**: 1-9.

[2] Suzuki A., Yukawa H., Nambu T., Matsumoto Y., Murata Y., 2016. Quantitative Evaluation of Hydrogen Solubility and Diffusivity of V-Fe Alloys toward the Design of Hydrogen Permeable Membrane for Low Operative Temperature. *Materials Transactions.* **57**: 1823-1831.

[3] Dolan M. D., McLennan K. G., Way J. W., 2012. Diffusion of atomic hydrogen through V-Ni alloy membranes under nondilute conditions. *The journal of physical chemistry.* **116**: 1512-1518.

[4] Brutti S., Tosti S., Santucci A., Paolone A., 2019. Deuterium absorption properties of $V_{85}Ni_{15}$ and evidence of isotope effect. *International journal of hydrogen energy.* **44**: 20145-20149.

[5] Alimov V.N., Busnyuk A.O., Notkin M.E., Peredistov E.Yu., Livshits A.I., 2004. Substitutional VePd alloys for the membranes permeable to hydrogen: Hydrogen solubility at 150 and 400 °C. *International journal of hydrogen energy*. **39**: 19682-19690.

[6] Fromm E, Gebhardt E. Gase und Kohlenstoff in Metallen. Berlin: Springer; 1976. ISBN: 978-3-642-80944-6.

[7] Schober T. 1996. Vanadium-, niobium- and tantalum-hydrogen. *Solid State Phenom*. **49-50**: 357-422.

[8] Yukawa Y., Nambu T., Matsumoto Y., Watanabe N., Zhang G., Morinaga M., 2008. Alloy Design of Nb-Based Hydrogen Permeable Membrane with Strong Resistance to Hydrogen Embrittlement. *Materials Transactions.* **49**: 2202-2207

[9] Taxak M., Kumar S., Krishnamurthy N., 2016. Thermodynamic Parameters for the Solubility of Hydrogen in Tantalum-Aluminium Alloys. *Innov Ener Res.* **5**: 144.

[10] Palumbo O., Trequattrini F., Sarker S., Hulyakar M., Pal N., Chandra D., Dolan M., Paolone A., 2017. New Studies of the Physical Properties of Metallic Amorphous Membranes for Hydrogen Purification. *Challenges.* **8**: 1-12.

[11] Palumbo O., Trequattrini F., Pal N., Hulyalkar M., Sarker S., Chandra D., Flanagan t., pnichk M., Paolone A., 2017. Hydrogen absorption properties of amorphous $(Ni_{0.6}Nb_{0.4-y}Ta_{y})_{100-x}Zr_{x}$ membranes. *Progress in Natural Science: Materials International.* **27**: 126-131.

[12] Yen P. S., Deveau N: D., Datta R., 2017. Sandwiched liquid metal membrane (SLiMM) for hydrogen purification. *AIChE.* **63**: 1483-1488.
